# A Radical-Cationic Covalent Organic Framework to Accelerate Polysulfide Conversion for Long-Durable Lithium–Sulfur Batteries

**DOI:** 10.1021/jacs.5c09421

**Published:** 2025-08-19

**Authors:** Sijia Cao, Pouya Partovi-Azar, Jin Yang, Dongjiu Xie, Timo Held, Gianluca Marcozzi, Joseph E. McPeak, Wei Zhang, Xia Zhang, Markus Osenberg, Zdravko Kochovski, Changxia Li, Daniel Sebastiani, Johannes Schmidt, Moritz Exner, Ingo Manke, Arne Thomas, Wenxi Wang, Yan Lu

**Affiliations:** † 28340 Helmholtz-Zentrum Berlin für Materialien und Energie, Hahn-Meitner-Platz 1, 14109 Berlin, Germany; ‡ Institute of Chemistry, 9176 Martin Luther University Halle-Wittenberg, Von-Danckelmann-Platz 4, 06120 Halle (Saale), Germany; § Department of Chemistry, Functional Materials, 26524 Technische Universität Berlin, Hardenbergstraße 40, 10623 Berlin, Germany; ∥ Fachbereich Physik, Freie Universität Berlin, Arnimallee 14, 14195 Berlin, Germany; ⊥ Department of Chemistry, University of Copenhagen, Universitetsparken 5, 2100 Copenhagen, Denmark; # Department of Chemistry, School of Science, 557712 Westlake University, 600 Dunyu Road, Hangzhou, Zhejiang 310024, China; ¶ Institute of Chemistry, Humboldt Universität zu Berlin, Brook-Taylor-Straße 2, 12489 Berlin, Germany; ■ Institute for Technical and Environmental Chemistry, Friedrich-Schiller-Universität Jena, 07743 Jena, Germany; △ Helmholtz Institute for Polymers in Energy Applications, Jena, Lessingstrasse 12–14, 07743 Jena, Germany

## Abstract

Covalent organic frameworks (COFs) have emerged as promising metal-free sulfur hosts to facilitate the conversion kinetics and suppress the shuttling effect of lithium polysulfides (LiPSs) in lithium–sulfur (Li–S) batteries. However, constructing COFs with stable and high electrocatalytic functionality for LiPS conversion remains unexplored. Herein, we develop a radical-cationic COF (R-TTF^•+^-COF) with superior electrical conductivity of 3.9 S m^–1^ at room temperature, which features both nucleophilic and electrophilic sites for effective LiPS chemisorption and conversion. With this novel radical-based catalyst, the Li–S battery achieves superior longevity of 1500 cycles with a capacity fading of 0.027% per cycle at a current density of 0.5 C. The capacity retention of the Li–S battery based on R-TTF^•+^-COF at the current density of 2.0 C is nearly twice as high compared to a COF without radicals. The crucial role of radical cations in catalyzing LiPS conversion has been systematically elucidated through solid-state nuclear magnetic resonance spectroscopy, electron paramagnetic resonance spectroscopy, and theoretical simulations, which verify the reversible interactions between LiPSs and [TTF]_2_^•+^ moieties. This intriguing radical-assisted mechanism opens a new avenue for designing efficient catalytic sulfur hosts using organic molecules, offering a significant step toward the practical application of Li–S batteries.

## Graphical abstract

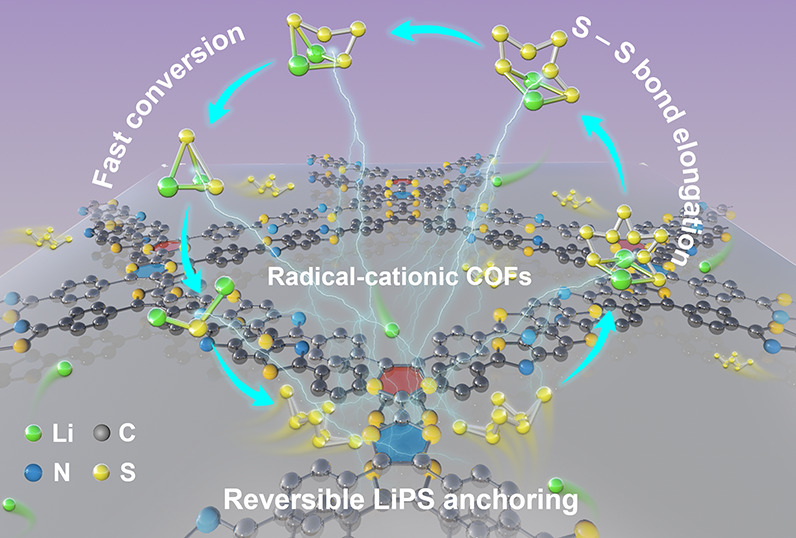

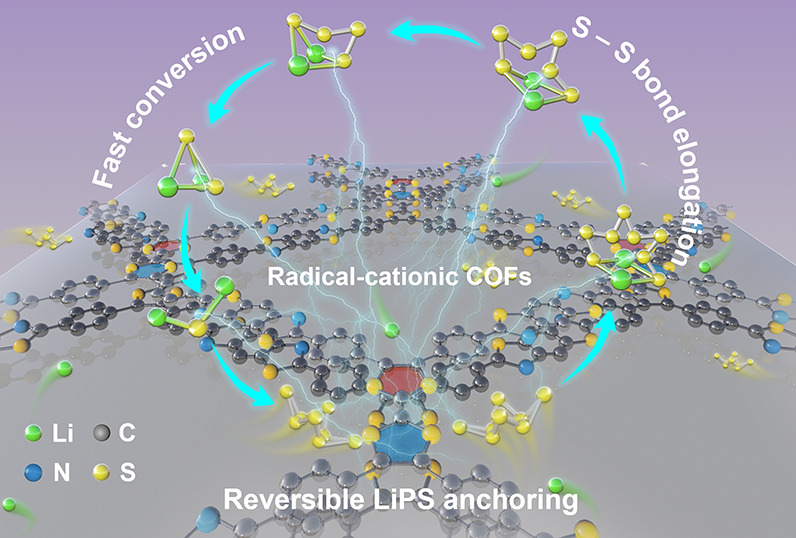

## Introduction

Covalent organic frameworks (COFs) are an emerging class of crystalline organic polymers featuring high porosity, structural tunability, low density, and chemical stability.^1,2^ The functionality and electronic structures of COFs can be fine-tuned at the molecular/atomic level through a sophisticated design of linkers and linkages, making them particularly interesting for energy storage applications.^3,4^ Recently, various COFs^5–12^ have been applied as metal-free sulfur hosts in Li–S batteries, aiming to solve the shuttling problem, the sluggish redox kinetics of lithium polysulfides (LiPSs), and large volumetric expansion in lithium–sulfur (Li–S) batteries. It is usually suggested that the COFs facilitate the physical/chemical confinement of LiPSs within their defined pore structures^5–8,11^ or by forming covalent sulfur bonds to COFs.^9,10^ However, these studies mostly show only limited success in boosting LiPS conversion and achieving a durable cycling performance in Li–S batteries. Pioneering investigations^13–15^ have shown that a suitable catalytic sulfur host can diminish the reaction activation barrier while adsorbing sulfur species, thereby reducing the accumulation of soluble LiPSs and mitigating the shuttle effect. Despite these findings, the electrocatalytic properties of COF-based sulfur cathodes are still rarely explored in Li–S batteries, due to the challenge of integrating highly efficient electrocatalytic sites into pure organic systems.

Organic radicals are highly reactive open-shell species that can impart some unique physical and chemical properties to materials.^16^ Radical-mediated catalysis, in particular, enables the formation of carbon-heteroatom bonds^17^ and facilitates reversible dynamic combination/dissociation reactions.^18^ The different types of radicals (e.g., carbon-,^19^ oxygen-,^20,21^ sulfur-,^22^ and nitrogen-centered^23^ radicals) can lead to distinct catalytic pathways and selectivity due to their varying stability, polarity, and reactivity.^24^ However, radicals are typically thermodynamically and kinetically unstable. The incorporation of radicals into COFs can stabilize them while maintaining their reactive nature.^19–21,25,26^ The unpaired electrons within micro/mesopores of COFs play an important role in constructing π-delocalized frameworks with facilitated interlayer charge transfer,^25,26^ as well as in exposing single-electron sites that can participate in bonding.^27^ Thus, integrating radical moieties in COFs presents a promising strategy for regulating their electronic structure and enhancing the catalytic properties in Li–S batteries. The type of radicals, together with the electronic properties of the framework, synergistically determines the catalytic pathways and activity. Nevertheless, COFs with stable radical building blocks specifically tailored for catalyzing sulfur reduction reactions (SRR) have yet to be explored.

In this work, we present a radical-cationic COF (R-TTF^•+^-COF) as a catalytic sulfur host for accelerating the conversion kinetics of LiPSs (Figure 1a). Via a one-pot sulfurization, the tetrathiafulvalene (TTF) moiety is oxidized by sulfur to generate a mixed-valence resonance structure (TTF^•+^).^28,29^ Subsequently, the paramagnetic dimeric [TTF]_2_^•+^ species form within adjacent bilayers of the close-packed TTF lattices of the COF,^26,28–30^ which is accompanied by the reduction of sulfur to short-chain anions^31–33^ (Figure 1b). Simultaneously, the imine linkages in the COF are converted to thiazole linkages by sulfur (Figure 1c),^34^ forming a more stable and conjugated structure. The sulfurized R-TTF^•+^-COF thus forms a stable, long-range ordered radical-cationic alignment and an expanded π-π conjugated structure with superior electrical conductivity of 3.9 S m^–1^. By examining the electrochemical properties of R-TTF^•+^-COF in Li–S batteries, it was found that the radical cations strengthen the adsorption capability of polysulfides and expose a high catalytic activity for polysulfide conversion. A combination of solid-state nuclear magnetic resonance (ssNMR) spectroscopy, electron paramagnetic resonance (EPR) spectroscopy with theoretical computation shows that the radical cations serve as catalytic centers that effectively immobilize LiPSs and facilitate the elongation and cleavage of the S–S bonds. Accordingly, the R-TTF^•+^-COF endows Li–S batteries with a cycling lifetime of as long as 1500 cycles at a current density of 0.5 C, with a capacity fading of only 0.027%. Such durable Li–S batteries have not yet been achieved with COF materials. This study broadens the design strategy of organic materials as promising electrocatalysts, thereby advancing the research frontline of Li–S batteries.

**Figure 1:**
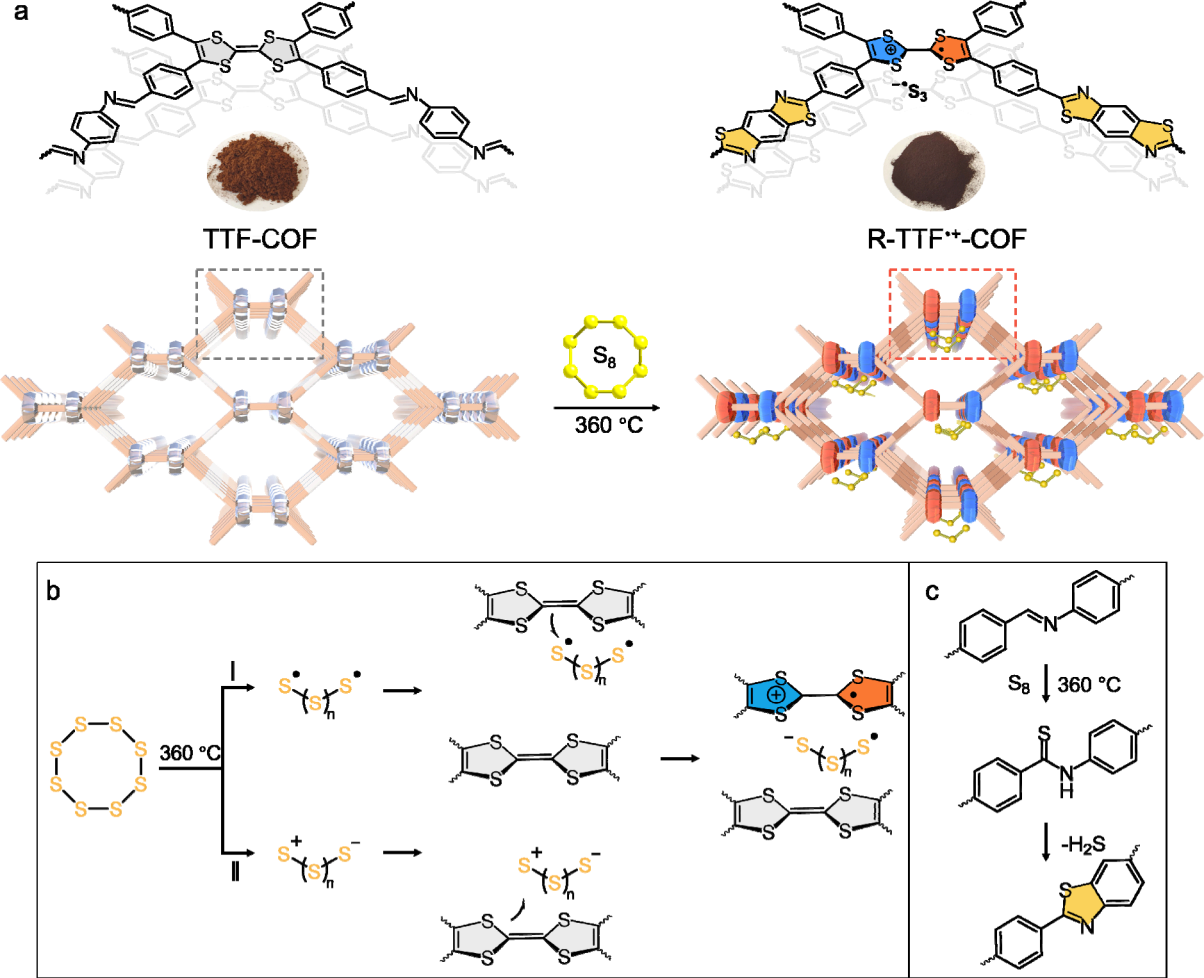
(a) The synthetic route to obtain R-TTF^•+^-COF from TTF-COF, where the possible conversion mechanism of TTF-COF to R-TTF^•+^-COF is shown: (b) The oxidation and stabilization of TTF moieties by sulfur radical anions. The ring-opening of S_8_ molecules may proceed at high temperatures via the homolytic cleavage (I) to generate thiyl diradicals or by heterolytic cleavage (II), which thereby engenders the sulfur ions. These species are highly reactive electrophilic reagents that attack the electron-rich TTF groups. As a result, the TTF moieties lose electrons and are oxidized to stable radical cations. (c) The oxidation routine of the imine-linkage to a thiazole-linkage.

## Results and Discussion

### Synthesis and Characterization of R-TTF^•+^-COF

The imine-linked TTF-COF was synthesized from 1,4-phenylenediamine and 4,4′,4″,4′′′-([2,2′-Bi­(1,3-dithiolylidene)]-4,4′,5,5′-tetrayl)­tetrabenzaldehyde in a mixed solvent of 1,4-dioxane/mesitylene catalyzed with 3 M acetic acid via solvothermal reaction (Scheme S1). Subsequently, R-TTF^•+^-COF was prepared by post-treating the TTF-COF powder with elemental sulfur (S_8_) at 360 °C. The excess S_8_ was then removed with toluene. The reaction conditions were optimized by systematically varying the temperature (i.e., 200, 300 and 360 °C) and reaction time (from 2 to 5 h), as detailed in Figure S1 and Table S1. Notably, sulfurization at 360 °C for 5 h not only ensures the complete conversion of imine bonds into benzothiazole linkages, but also promotes sufficient oxidation of tetrathiafulvalene (TTF) to [TTF]_2_^•+^. This process leads to the formation of stable radical species and the construction of an extended conjugated framework (R-TTF^•+^-COF). After post-treatment at 360 °C, the color of the powder turned from reddish brown of TTF-COF to dark brown of R-TTF^•+^-COF, suggesting more extensive electronic conjugation in R-TTF^•+^-COF. The crystalline structures of the two COFs were analyzed using powder X- ray diffraction (PXRD) combined with structural simulations. The diffraction peaks of TTF-COF appear at 3.72°, 4.45°, 5.89°, and 7.53°, indexed as (110), (010), (200), and (220) facets, respectively, which match well with the simulated AA-stacked model (Figure S2a). The dominant peaks of R-TTF^•+^-COF at 3.86° and 7.69° are assigned to (110) and (220) reflections with a slight shift to higher angles, respectively (Figure 2a). The simulated PXRD pattern (blue curve) agrees well with the experimentally observed pattern (red curve), as evidenced by the negligible difference (gray curve) in Pawley refinement. These PXRD patterns indicate that R-TTF^•+^-COF maintains its crystal structure, as the decrease in intensity of the reflections can be attributed to partial pore filling by the sulfur moieties. More importantly, the PXRD of R-TTF^•+^-COF shows no reflections from S_8_, implying that sulfur is homogeneously distributed and chemically bonded in the COF skeleton (Figure 2a and Figure S3). The partial pore filling of the COF pores by sulfur has been also seen in the N_2_ adsorption–desorption isotherms, indicating the typical microporous characteristics for both COFs, accompanied by a decrease in Brunauer–Emmett–Teller surface area (*S*_BET_) and pore volume from 1015 m^2^ g^–1^ and 0.846 cm^3^ g^–1^ for TTF-COF to 281 m^2^ g^–1^ and 0.234 cm^3^ g^–1^ for R-TTF^•+^-COF (Figure 2b, Figure S2b-d). The pore dimensions of TTF-COF and R-TTF^•+^-COF are compatible with the sizes of LiPS species,^35^ which can enable effective accommodation and transport of LiPSs within the channels while simultaneously providing spatial confinement. Cryogenic low-dose transmission electron microscopy (TEM) was employed to visualize the crystallinity and pore structure of the COFs. High-resolution TEM (HRTEM) images confirm the crystalline quality of the two COFs (Figure 2c and Figure S4a-b). The inverse fast Fourier transform (FFT) images further reveal the two-dimensional rhombic topology along the [001] direction for the two COFs (Figure 2c and Figure S4c). Scanning electron microscopy (SEM) and energy dispersive X-ray (EDX) mapping demonstrate the homogeneous distribution of sulfur in R-TTF^•+^-COF, thereby verifying the uniform incorporation of sulfur into the COF backbone (Figure S5–7).

**Figure 2:**
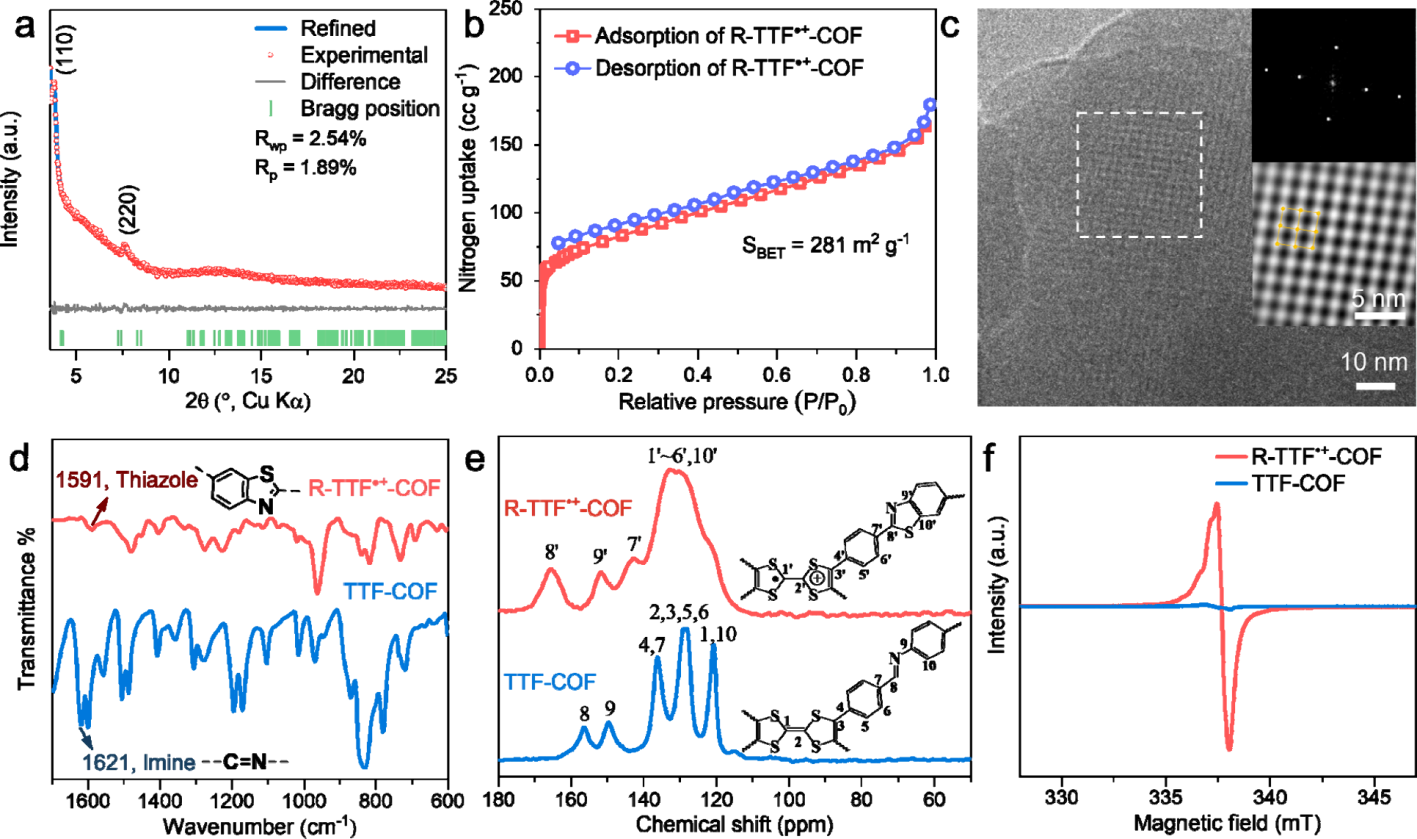
Characterization of R-TTF^•+^-COF. (a) Experimental and simulated PXRD patterns (unit cell parameter: *a* = 24.492 Å, *b* = 23.948 Å, *c* = 8.044 Å, α = 90.692°, β = 90.887°, γ = 60.692°). (b) N_2_ adsorption–desorption isotherms of R-TTF^•+^-COF. (c) Low-dose HRTEM image of R-TTF^•+^-COF under cryogenic conditions. Insets show the FFT (top) and inverse FFT (bottom) of the region indicated by the white square. (d) FTIR spectra, (e) ^13^C CP/MAS solid-state NMR spectra, and (f) solid-state EPR spectra of TTF-COF and R-TTF^•+^-COF.

The structural transformation from TTF-COF to R-TTF^•+^-COF was confirmed by a combination of techniques. The Fourier transform infrared (FTIR) spectra (Figure 2d) show that the imine linkage at 1621 cm^–1^ in TTF-COF shifts to 1591 cm^–1^ in R-TTF^•+^-COF, indicating the formation of a benzothiazole ring.^34^ The characteristic bands for the TTF moiety in R-TTF^•+^-COF also differ from those in TTF-COF (Table S2), confirming the structural differences of the TTF groups in the two COFs.^36^ The reaction intermediates from imine to benzothiazole were investigated by *in situ* thermogravimetry-mass spectrum (TG-MS) analysis. There appears a sharp ion current with *m*/*z* value of 34 at the reaction time of 115 min, which can be assigned as H_2_S species (Figure S8). The observed formation of H_2_S strongly corroborates the reaction mechanism proposed in Figure 1c. Moreover, the solid-state 2D ^1^H–^13^C heteronuclear correlation (HETCOR) NMR spectra have identified the chemical environment surrounding carbon and hydrogen in the two COFs (Figure 2e, and Figure S9–10). The overlapping carbon positions were assigned based on the bound protons, as shown in Figure 2e. In TTF-COF, the chemical shifts at 149 (C9) and 156 ppm (C8) can be exclusively assigned to the imine carbons. The resonance peaks of C9 and C8 shift downfield in R-TTF^•+^-COF (C9′ = 152 and C8′ = 165 ppm) such that the correlation between C8/C10, and the protons disappears, cumulatively supporting the successful oxidation of the imine bonds by sulfur. The peaks correlated with C1–C6 in TTF-COF merge into a single broad hump in R-TTF^•+^-COF, indicating a structural transformation. In particular, the chemical shifts of C1′ and C2′ in R-TTF^•+^-COF are higher than those of C1 and C2 in TTF-COF, which is attributed to the reduced electron density in the TTF unit caused by the structural transformation from ethylene to aromatic character.^37^ This phenomenon is consistent with the ^13^C ssNMR spectrum of TTF^•+^ formed by iodine oxidation,^38^ thus confirming the validity of our proposed structure.

The radical properties of COFs were characterized by solid-state EPR spectroscopy (Figure 2f). R-TTF^•+^-COF possesses an intense radical signal, and the peak-to-peak intensity is increased by a factor of ∼50, compared to the negligible signal intensity of TTF-COF. This confirms the formation of stable radicals in the COF structure. Considering the closely stacked structure within the COF, adjacent TTF^•+^ units might form diamagnetic and thus EPR silent species, which points to the formation of mixed-valence [TTF]_2_^•+^ structures in the close-stacked layers of COFs (Scheme S2).^26^ To further verify the formation of [TTF]_2_^•+^ fragments in R-TTF^•+^-COF, cyclic voltammetry (CV) measurements of the two COFs were conducted in a three-electrode system. Notably, the TTF groups in TTF-COF undergo three-step oxidation (Figure S11), first oxidizing the nonaromatic [TTF]_2_ to [TTF]_2_^•+^ (I), followed by transformation to the [TTF^•+^]_2_ dimer (II) and then to the dicationic [TTF^+^]_2_ (III). In contrast, R-TTF^•+^-COF only exhibits transformations II and III. These differences in CV profiles strongly support the formation of the mixed-valence [TTF]_2_^•+^ units in R-TTF^•+^-COF.^30^ Accordingly, a singly charged counteranion should be in proximity to compensate for the positive charge in the [TTF]_2_^•+^ unit. Using Raman spectroscopy, a new vibration at 523 cm^–1^ was detected, which can be assigned to S_3_^•–^ in R-TTF^•+^-COF (Figure S12).^39^ Similarly, the presence of trisulfides in R-TTF^•+^-COF was identified at 162.7 eV in the high-resolution XPS S 2p spectra (Figure S13).^40^ The detailed assignment is discussed in Tables S3–4. Moreover, elemental analysis and TGA profiles of the two COFs further indicate that trisulfides are incorporated into the COF backbones (Figure S14 and Table S1). Although the size of S_3_^•–^ is compatible with the interlayer spacing of R-TTF^•+^-COF, these species preferentially localize near the [TTF]_2_^•+^ sites along the pore walls (Figure 1a), indicated by density functional theory (DFT) calculation. Therefore, the repeating units were determined to be [TTF]_2_^•+^ S_3_^•–^ in R-TTF^•+^-COF.

### Electronic Structure of R-TTF^•+^-COF

Through the oxidation of the TTF segments and the transformation of the imines into thiazole linkages, the electronic structure of R-TTF^•+^-COF undergoes significant variation and thereby leads to a distinct electron transfer mechanism and redox activity. Ultraviolet–visible-near-infrared diffuse reflectance spectroscopy (UV–vis-NIR/DRS) was employed to ascertain the electronic structures of the two COFs. As shown in Figure S15, R-TTF^•+^-COF exhibits a broader and stronger absorption than TTF-COF in the range of 630 – 1400 nm. This long-wavelength absorption can be attributed to the columnar alignment of open-shell radicals in R-TTF^•+^-COF.^25^ Derived from Tauc Plots, R-TTF^•+^-COF displays a significantly lower optical bandgap (0.66 eV) than TTF-COF (1.15 eV). As shown in Figure 3a, the highest occupied molecular orbital (HOMO) of TTF-COF is predominantly distributed over the TTF moieties, while the lowest unoccupied molecular orbital (LUMO) comprises the imine groups and neighboring phenyl groups. In contrast, the HOMO and LUMO are centered over the [TTF]_2_^•+^ rings and trisulfides in R-TTF^•+^-COF. Accordingly, the calculated bandgap of R-TTF^•+^-COF (*E*_g_ = 0.434 eV) is much smaller than that of TTF-COF (*E*_g_ = 1.401 eV), consistent with the trend observed in the UV–vis-NIR/DRS spectra. Herein, the unpaired electron is delocalized across both layers rather than being localized to a single TTF, indicating a preferable interlayer charge transfer in the mixed-valence [TTF]_2_^•+^. The spatial distribution of the two spins in the R-TTF^•+^-COF under full incorporation of the condensed-phase packing structure was also computed using DFT. Figure 3b shows a clear localization of the two spin densities on the [TTF]_2_^•+^ and trisulfide, which is fully consistent with the strong dipolar coupling visible in the distorted EPR line shape. The density of states (DOS) was calculated to further clarify the changes in the electronic structure (Figure S16). The formation of the radical introduces additional electronic states near the Fermi level, resulting in an increased DOS in this region. Notably, this leads to a clear narrowing of the band gap in the R-TTF^•+^-COF system compared to the pristine TTF-COF. The two states (α-spin and β-spin) near the Fermi level correspond to the singly occupied molecular orbitals (SOMO), which effectively reduce the energy gap between the HOMO and LUMO. The increased DOS near the Fermi level following radical formation suggests enhanced electronic conductivity and a greater number of active sites available for polysulfide adsorption. Such electronic structure would facilitate electron transfer from the electrocatalyst to the antibonding orbitals of the S–S bonds in Li–S cathodes, thereby weakening the bonds and promoting polysulfide activation.^41–43^ The electrical conductivity of the two COF powder samples was measured at room temperature using the four-probe method. Each sample was tested in pellet form with five parallel measurements to ensure reproducibility. The electrical conductivity of TTF-COF was found to be 3.2 × 10^–6^ S m^–1^, while that of R-TTF^•+^-COF reached 3.9 S m^–1^ — a 10^6^-fold increase compared to the pristine COF (Table S5–6). The result aligns well with the calculated HOMO/LUMO gap values. This superior conductivity exceeds that of most previously reported conductive COFs (Table S7).

**Figure 3:**
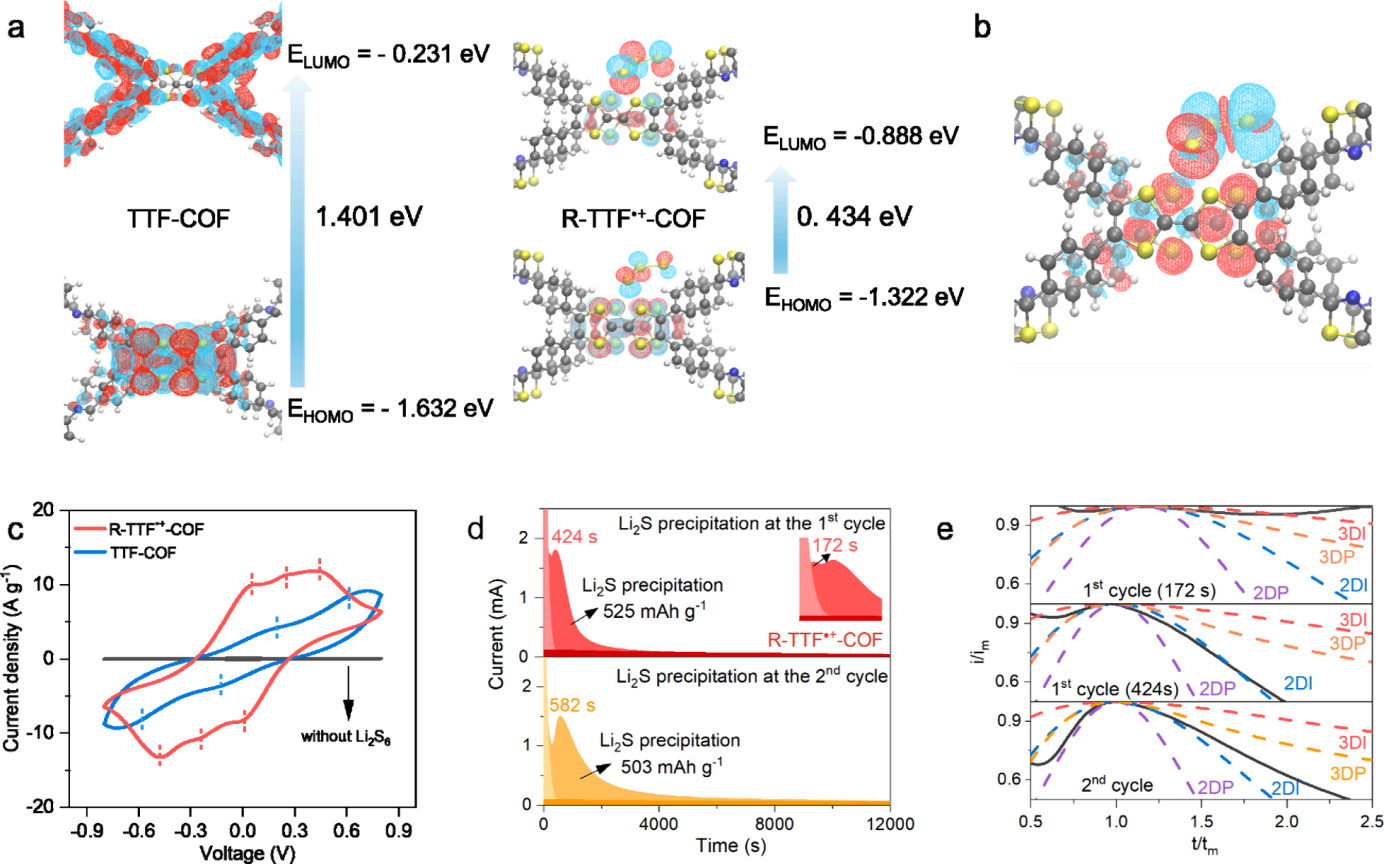
Electronic structures and catalytic properties of R-TTF^•+^-COF. (a) HOMO/LUMO distributions of the adjacent bilayer molecular units in TTF-COF and R-TTF^•+^-COF and their corresponding energy levels. (b) The spin density plots of R-TTF^•+^-COF calculated via DFT by consideration of the interaction between neighboring [TTF]_2_^•+^ and S_3_^•–^, where the red region designates α-spin, and the blue region designates β-spin. (c) CV curves of symmetric cells in the voltage window of −0.8–0.8 V at a scan rate of 5.0 mV s^–1^ in Li_2_S_6_ electrolyte. (d) The potentiostatic Li_2_S precipitation process at 2.05 V on an R-TTF^•+^-COF electrode at the first and second cycles. (e) The corresponding dimensionless transient of R-TTF^•+^-COF electrode at the first and second cycles fitted with theoretical 2D/3D models, respectively (t_m_ denotes the time needed to reach the peak current, i_m_ denotes the peak current).

### Catalytic Properties of R-TTF^•+^-COF toward LiPSs

The radical-cationic structure of R-TTF^•+^-COF could offer electrocatalytic activity toward LiPS conversion due to its open-shell electronic configuration. Given this, static polysulfide adsorption and electrochemical measurements were employed. After immersing 5 mg COF powders into 4 mL 1 mM Li_2_S_6_ solution for 1 h, the R-TTF^•+^-COF-containing solution became nearly colorless, whereas the TTF-COF solution showed no visible color variation (Figure S17). Moreover, the UV–vis absorption peak representing Li_2_S_6_ is almost invisible for the R-TTF^•+^-COF solution, sufficiently reflecting the strong adsorption capability of R-TTF^•+^-COF for LiPSs. The catalytic effect of COFs on the LiPS conversion was evaluated by CV measurements based on symmetric COF|COF cells. As shown in Figure 3c, R-TTF^•+^-COF exhibits three pairs of redox peaks at −0.05/0.05 V, −0.23/0.23 V, and −0.43/0.43 V, respectively. Among these, the redox peaks at −0.05/0.05 V correspond to the interaction between R-TTF^•+^-COF and Li^+^, as these peaks are also present in the CV profile without Li_2_S_6_ (Figure S18). The other two pairs of peaks are associated with the conversion between Li_2_S_8_, Li_2_S_6_ and Li_2_S_4_. In contrast to R-TTF^•+^-COF, TTF-COF shows two pairs of negligible broad peaks at −0.13/0.13 and −0.64/0.64 V with lower integral peak areas than that of R-TTF^•+^-COF. As expected, the radical-cationic COF features high catalytic activity for LiPSs.

The catalytic kinetics for Li_2_S nucleation and growth were further quantitatively investigated with Li_2_S_8_ catholyte. The electrodeposition kinetics on the R-TTF^•+^-COF electrode (172 s) is much faster than that on the TTF-COF electrode (2019 s), along with a higher current response at the first cycle (Figure 3d and Figure S19). According to Faraday’s law, R-TTF^•+^-COF contributes to a precipitation capacity as high as 525 mAh g^–1^, outperforming the lower capacity of the TTF-COF electrode at 316 mAh g^–1^. This indicates a higher catalytic conversion efficiency from long-chain LiPSs to Li_2_S in the R-TTF^•+^-COF catalyzed cell. The precipitation rate and capacity numerically highlight the significant electrocatalytic kinetics of R-TTF^•+^-COF toward the conversion of LiPSs. More notable is that R-TTF^•+^-COF displays two peaks during the first cycle, different from TTF-COF with a single hump, implying distinct Li_2_S nucleation and growth mechanisms for two COFs. Given this, the Bewick, Fleischmann, and Thirsk (BFT) 2D model and the Scharifker-Hills (SH) 3D model^44–47^ were applied to thoroughly examine the electrochemical deposition behavior (Table S8). The nucleation and growth of Li_2_S on TTF-COF comply with the 2D instantaneous (2DI) model (Figure S20), whereas the precipitation on R-TTF^•+^-COF initially proceeds via the 3D instantaneous (3DI) model and subsequently is governed by 2DI surface diffusion (Figure 3e). The 3D nucleation of R-TTF^•+^-COF at the early stage might be initiated by the reduction of S_3_^•–^ and the facilitated spatial diffusion due to the stronger adsorption of LiPSs and faster charge transfer in the R-TTF^•+^-COF (Figure S21). The continuous catalytic function of the R-TTF^•+^-COF electrode was further verified by the second Li_2_S precipitation after recharging the R-TTF^•+^-COF cell to 2.8 V. Impressively, the R-TTF^•+^-COF electrode remains a rapid response (582 s) with a comparable capacity (503 mAh g^–1^), as shown in Figure 3d. Notably, only one peak is shown in the second cycle, which can be ascribed to the consumption of trisulfides during the first precipitation, and the electrodeposition approaches the 2D model (Figure 3e). This result further indicates the stability of the catalytic effect of R-TTF^•+^-COF. In this scenario, the adsorption and catalytic performance jointly validate that the radical-cationic structure endows R-TTF^•+^-COF with catalytic properties to accelerate the electrochemical LiPS conversion kinetics.

### Electrochemical Performance of Li–S Batteries

The intrinsically high porosity and remarkable catalytic ability make R-TTF^•+^-COF an ideal candidate as a sulfur host material. The electrochemical performance of R-TTF^•+^-COF and TTF-COF was systematically studied in Li–S coin cells. The R-TTF^•+^-COF/S composite was fabricated by heating the TTF-COF/S mixture at 360 °C in a vacuum-sealed Pyrex tube. During this process, a minor portion of S_8_ oxidizes TTF-COF, forming R-TTF^•+^-COF and the remaining S_8_ impregnates into the R-TTF^•+^-COF matrix. As a reference, the TTF-COF/S mixture was prepared by physically mixing TTF-COF and S_8_ and heating at 155 °C. The sulfur mass loading in both composites is 69.4 and 68.5 wt %, respectively (Figure S22–26).

The redox processes of R-TTF^•+^-COF/S and TTF-COF/S cathodes were compared using CV measurements at a scan rate of 0.1 mV s^–1^ (Figure 4a). The CV profiles of both cathodes show a typical electrochemical redox behavior of elemental sulfur. Besides, the R-TTF^•+^-COF/S cathode has a much lower electrochemical polarization (325 mV) than that of TTF-COF/S (470 mV), agreeing well with the polarization potential gaps observed in the galvanostatic (dis)­charge profiles (Figure S27). Furthermore, the capacity ratio (*Q*_2_/*Q*_1_) of the second plateau (*Q*_2_) to the first plateau (*Q*_1_) further demonstrates the higher sulfur conversion efficiency of R-TTF^•+^-COF (2.8) compared to TTF-COF (2.0).^48^ To solidify the enhanced electrochemical performance, the rate capability of Li–S cells was measured at current densities ranging from 0.05 to 2.0 C and back to 0.1 C (Figure 4b). Specifically, the specific discharge capacities of the R-TTF^•+^-COF/S electrode reach 1140, 935, 825, 741, 681, 607, and 846 mAh g^–1^ at current densities of 0.05, 0.1, 0.2, 0.5, 1.0, 2.0, and 0.1 C, respectively. In contrast, the TTF-COF/S electrode delivers specific discharge capacities of 859, 680, 576, 477, 404, 320, and 611 mAh g^–1^ at the corresponding current densities.

**Figure 4:**
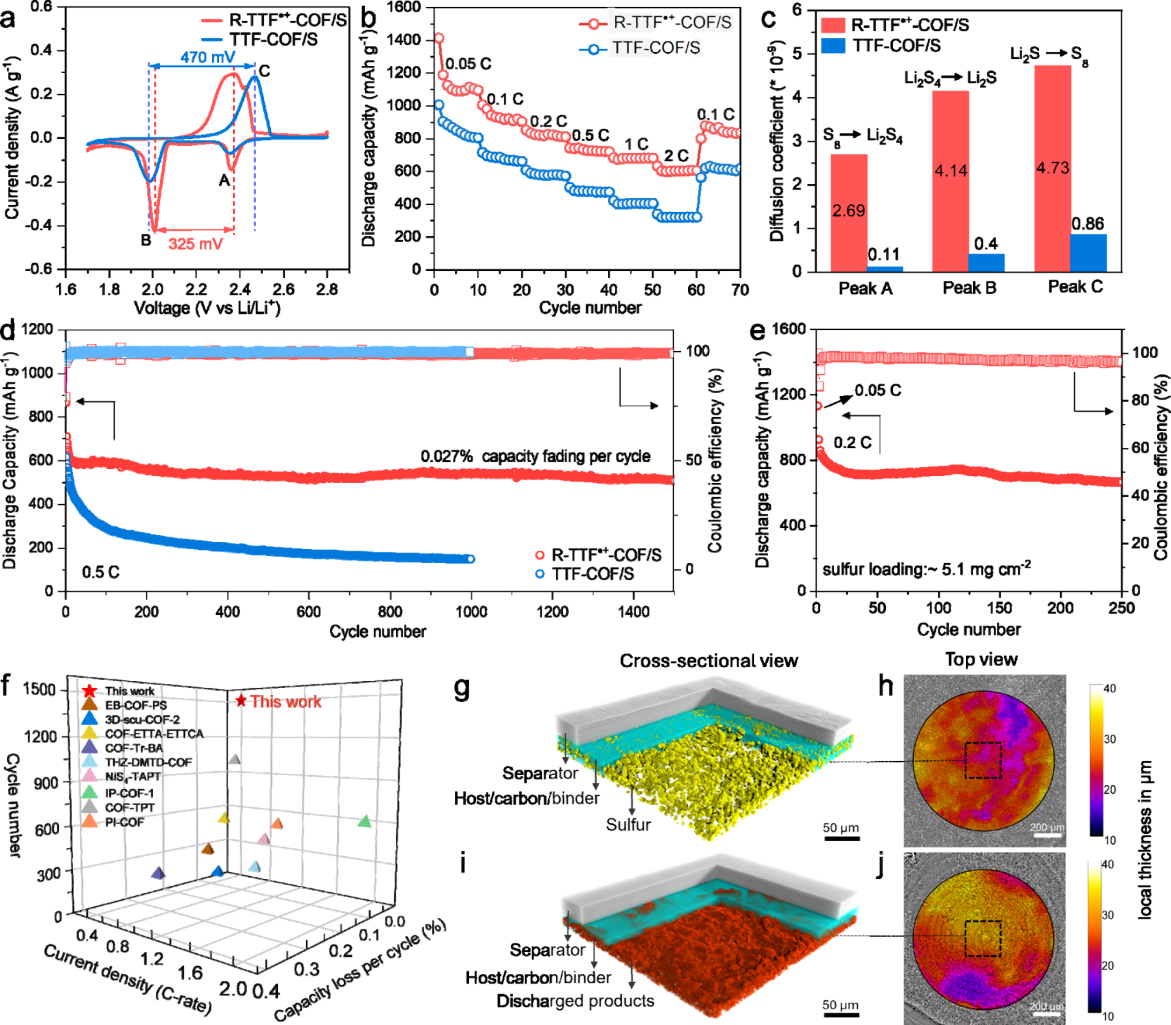
Electrochemical performance of R-TTF^•+^-COF/S and TTF-COF/S in Li–S batteries. (a) CV profiles at a scan rate of 0.1 mV s^–1^. (b) Rate capability at different current densities (1 C = 1675 mA g^–1^). (c) Diffusion coefficients calculated from CV at various scan rates in coin cells. (d) Long-term cycling at the current density of 0.5 C (Areal sulfur loading: ∼1.3 mg cm^–2^). (e) Cycling stability of the R-TTF^•+^-COF/S cathode with high sulfur loading at the current density of 0.2 C. The cell was first discharged at 0.05 C for activation. (Areal sulfur loading: ∼5.1 mg cm^–2^). (f) Comparisons of the cycle number, current density, and capacity fading per cycle for R-TTF^•+^-COF with recently reported COF as the sulfur host in Li–S batteries. The corresponding data are summarized in Table S9. *In situ* X-ray microtomographic 3D renderings of the R-TTF^•+^-COF/S cathode are shown in (g) at the initial state and (i) after the first discharge at the current density of 0.05 C in the regions marked by black squares in the top-view slices (h) and (j), respectively. The corresponding thickness distributions of the cathode are shown in (h) and (j).

R-TTF^•+^-COF significantly accelerates the conversion kinetics of LiPSs, which can be attributed to its high catalytic activity and lower charge-transfer resistance (*R*_ct_ = 123.2 Ω for R-TTF^•+^-COF compared to 340.1 Ω for TTF-COF) (Figure S28). The lithium-ion (Li^+^) diffusion coefficient (*D*_Li_^+^) was also calculated to measure the diffusion kinetics of Li^+^ (Figure S29). According to the Sevick-Randles equation,^49^ the diffusion coefficients of R-TTF^•+^-COF/S at peaks A, B, and C are calculated to be 2.69 × 10^–9^, 4.14 × 10^–9^ and 4.73 × 10^–9^, respectively, which is an order of magnitude higher than those of the TTF-COF/S electrode (1.10 × 10^–10^, 4.00 × 10^–10^ and 8.60 × 10^–10^) (Figure 4c). Thus, the facilitated diffusion kinetics by R-TTF^•+^-COF are consistent with the improved rate capability.

Long-term stability is one of the critical indicators for validating the practicability of the R-TTF^•+^-COF/S cathode in Li–S batteries. R-TTF^•+^-COF/S allows Li–S cells to sustain their capacity following up to 1500 cycles with only 0.027% capacity fading per cycle with an initial energy density of 225 Wh kg^–1^ at the current density of 0.5 C, considerably outperforming the TTF-COF/S electrode (Figure 4d). The relatively low Coulombic efficiency observed during the first three cycles can be attributed to the activation process required for the full utilization of sulfur in the initial stages of cycling.^50^ This impressive cycling performance also surpasses most reported COF materials in terms of lifespan and capacity loss ratio (Figure 4f and Table S9). Such good durability of Li–S batteries shows the catalytic activity of R-TTF^•+^-COF in suppressing the LiPS shuttling while elevating the utilization efficiency of sulfur species. The shuttle current was then measured to determine the inhibition functions of R-TTF^•+^-COF for LiPSs. R-TTF^•+^-COF/S exhibits a smaller shuttle current than TTF-COF/S cells, demonstrating the significant suppression of LiPSs by R-TTF^•+^-COF (Figure S30). To further examine the potential practical application for R-TTF^•+^-COF, Li–S cells with a high sulfur loading of up to 5.1 mg cm^–2^ were assembled, which persists for 250 cycles with capacity retention of 72% with an initial energy density of 463 Wh kg^–1^ at the current density of 0.2 C (Figure 4e). In addition, the electrochemical performance is evaluated at the pouch cell scale with a cell size of 4.4 cm × 5.7 cm. The pouch cell retains 85% of its initial capacity after 50 cycles at 0.2 C. Even after 200 cycles, it still delivers a capacity of 464 mAh g^–1^ (Figure S31a). Impressively, the pouch cell was capable of powering over 90 LED lights, highlighting the practical application potential of the COF-catalyzed Li–S battery (Figure S31b). To assess the structural stability of the COF, R-TTF^•+^-COF was immersed in the electrolyte for 1 week. The material preserved its crystallinity and the benzothiazole linkages, as confirmed by PXRD and FTIR spectra (Figure S32). Furthermore, the stability of the radical species was examined by EPR spectroscopy after 1500 cycles, revealing nearly unchanged signal intensity (Figure S33). These results collectively demonstrate the high catalytic durability and robust structural stability of the R-TTF^•+^-COF under practical operating conditions.

COFs and MOFs have been considered to effectively reduce the volumetric expansion of cathodes in Li–S batteries thanks to their high porosity;^3^ however, this has not yet been convincingly proven. In this study, *in situ* X-ray microtomography was utilized to visualize the accommodation capacity of COF pores for sulfur species. From the cross-sectional images and 3D renderings of the R-TTF^•+^-COF/S cathode, the sulfur particles observed in the fresh cathode completely disappear after the first discharge at a current density of 0.05 C, despite using a cathode with ∼5.1 mg cm^–2^ sulfur loading (Figure S35 and Figure 4g, i). Furthermore, the thickness distribution analysis of the R-TTF^•+^-COF/S cathode shows a small volume expansion ratio of ∼7.2% after the first discharge (Figure S36 and Figure 4h, j). This measurement demonstrates the ability of the COF to effectively host sulfur species, which mitigates the large volumetric expansion resulting from the lithiation of S_8_.

### Catalytic Mechanism of R-TTF^•+^-COF in Li–S Batteries

To gain insight into the role of the radical-cationic R-TTF^•+^-COF in LiPS conversion, ^13^C and ^7^Li ssNMR techniques were employed to track the evolution of the chemical environment surrounding carbon and lithium on the R-TTF^•+^-COF backbone during the electrochemical process (Figure 5a-c).

**Figure 5:**
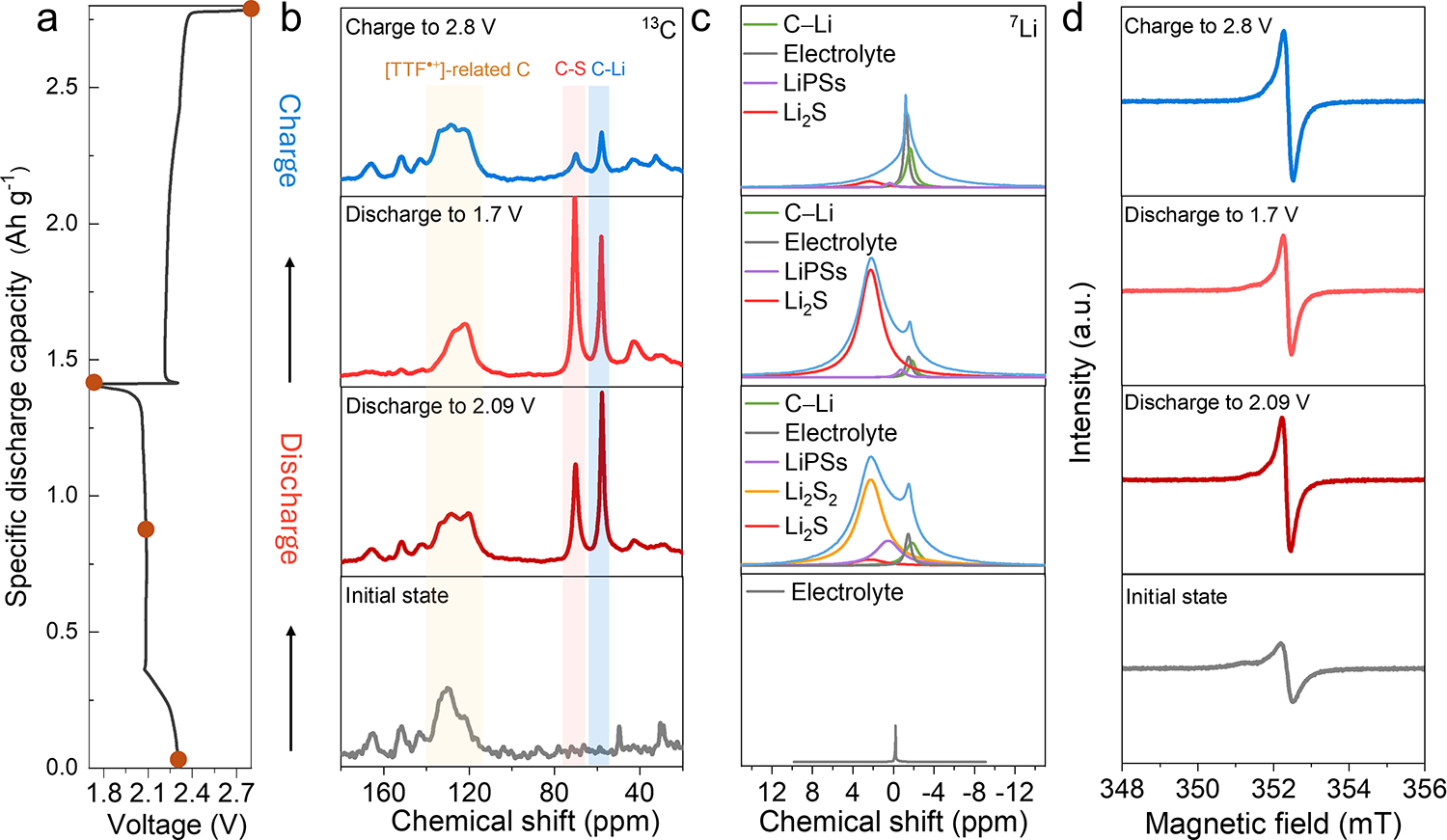
Analysis of catalytic behavior of R-TTF^•+^-COF. (a) Galvanostatic (dis)­charge profile of R-TTF^•+^-COF/S cathode. (b) Solid-state CP/MAS ^13^C NMR and (c) ^7^Li NMR spectra. (d) Solid-state EPR spectra measured at X-band during the first cycle under the current density of 0.05 C.

In the ^13^C NMR spectra, upon discharging to 1.7 V, the peaks representing TTF^•+^ groups overall shift upfield. While recharging to 2.8 V, the chemical shift and the peak shape nearly return to the initial state (Figure 5b). The peak shift is attributed to the electron shielding originating from the chemical interaction between sulfide species (Li_2_S_*n*_, 1≤ *n* ≤ 8) and TTF^•+^ units. Simultaneously, two peaks at 58 and 70 ppm emerge upon discharging from the initial state to 1.7 V, indicating the interplay between carbon and lithium (C–Li)^51^ as well as carbon and sulfur (C–S)^52^ on TTF^•+^, respectively. These variations reflect the chemical immobilization of LiPSs on TTF^•+^ units. Interestingly, the area ratio of the two peaks evolves with (dis)­charging, suggesting the dynamic binding and dissociation of sulfides on the TTF^•+^ groups; however, the two peaks remain as weak signals at the fully recharged state, possibly resulting from the partially irreversible combination of a few sulfides in proximity to TTF^•+^. Moreover, a new peak at 225.4 ppm becomes increasingly visible upon discharging from the initial state to 1.7 V and attenuates upon charging (Figure S37). This correlates with the generation of C=S^53^ in TTF^•+^ groups caused by the electron transfer on TTF^•+^ during the interaction with LiPSs. The interaction between TTF^•+^ and LiPSs primarily facilitate the cleavage of the S–S bonds and the separation of sulfide species from TTF^•+^ groups (Scheme S3). Additionally, the chemical shift of benzothiazole-related carbons (C7, C8, C9) remains unchanged at each state, which rules out the reactivity around the groups during the electrochemical process. The ^7^Li ssNMR spectra with deconvolved peaks are shown in Figure 5c. When discharged to 2.09 V, the ^7^Li ssNMR spectra reveal that sulfur is mostly converted to low- order lithium sulfides (Li_2_S_2_) at 2.18 ppm, with minor Li_2_S at 2.38 ppm.^54^ Li_2_S_2_ is further reduced to Li_2_S upon discharging to 1.7 V, while the Li_2_S peak nearly disappears on recharging to 2.8 V. In addition, the peak assigned to C–Li also appears at −1.5 ppm^55^ during discharge and persists when recharging to 2.8 V. Therefore, the ^13^C and ^7^Li spectra synergistically corroborate the catalytic activity of R-TTF^•+^-COF for LiPSs. Additionally, the FTIR signatures at 1082 cm^–1^ for C–Li,^56^ 868 cm^–1^ for C–S,^57^ and 1102 cm^–1^ representing C=S bonds^58^ are also indicative of the interactions between Li_2_S_n_ and TTF^•+^ carbon during discharging (Figure S38 and Table S10). Likewise, the high-resolution S 2p XPS spectra identify the products at different states, supporting the ssNMR results (Figure S39). Furthermore, *operando* Raman spectroscopy was employed to monitor the time-resolved evolution of LiPSs during the SRR. For the R-TTF^•+^-COF/S cathode (Figure S40a), signatures for elemental sulfur at 152, 218, 471 cm^–1^ disappear and characteristic Raman signals are observed at 445 cm^–1^ (Li_2_S_8_), 398 cm^–1^ (Li_2_S_6_ + Li_2_S_4_)^39,59^ near the first discharge plateau at 2.3 V (Table S3). These signals first strengthen by 2.25 V and subsequently weaken at 2.1 V and disappear around the second plateau at approximately 2.09 V, indicating the reduction of long-chain LiPSs to short-chain sulfides. Notably, a distinct peak at ∼521 cm^–1^, attributed to the S_3_^•–^, appears during the first plateau, suggesting that the R-TTF^•+^-COF significantly accelerates the cleavage of the S–S bond in LiPSs. In contrast, the TTF-COF/S cathode show negligible LiPS signals (Figure S40b), indicating the loss or dissolution of LiPSs in the cathode. These observations underscore the catalytic capability of the R-TTF^•+^-COF to effectively immobilize polysulfides and facilitate the SRR.

The solid-state EPR spectra were recorded to uncover the evolution of the radical center during (dis)­charging. The intensity of the EPR signal at discharge states of 2.09 and 1.7 V and at a charge state of 2.8 V is a factor of 2 greater compared to the initial sample (Figure 5d). This phenomenon indicates the irreversible change in the spin system of R-TTF^•+^-COF during the electrochemical process. In the initial R-TTF^•+^-COF, the spin density primarily originates from the contribution of [TTF]_2_^•+^ and S_3_^•–^ with a spin density value of ∼0.56. During the first discharging, trisulfides are consumed by lithiation and replaced bybis­[(trifluoromethyl)­sulfonyl]­imide (TFSI^–^), forming stable TFSI^–^-based radical cations (i.e., [TTF]_2_^•+^TFSI^–^). The spin density in R-TTF^•+^-COF with TFSI^–^ present was determined via calculation to be only delocalized over the [TTF]_2_^•+^ (Figure S41), showing only the α-spin state with a spin density value of ∼1.11. As a result, the larger spin density of R-TTF^•+^-COF with TFSI^–^ present leads to a remarkable increase in the intensity of the EPR signal. The bandgap of R-TTF^•+^-COF with TFSI^–^ is calculated to be 0.434 eV, identical to that of R-TTF^•+^-COF with S_3_^•–^ present (Figure S42). The unchanged value manifests the observation that both frontier orbital (HOMO and LUMO) levels are determined mainly by the radical cation [TTF]_2_^•+^ rather than the nearby anions. This is consistent with the high catalytic activity of the [TTF]_2_^•+^ center.

### Computational Study of R-TTF^•+^-COF as Electrocatalysts in Li–S Batteries

Considering that S_3_^•–^ is superseded by TFSI^–^ after discharge, the interaction energies and bond lengths are thus calculated using R-TTF^•+^-COF with TFSI^–^. The adsorption affinity of R-TTF^•+^-COF for LiPSs (Li_2_S_*n*,_ *n* = 4, 6, 8) was calculated. The atomic structures of LiPSs were first optimized in the gas phase and in the presence of R-TTF^•+^-COF (Figure S43–45). As depicted in Figure S46, the interaction energies of Li_2_S_8_, Li_2_S_6,_ and Li_2_S_4_ on R-TTF^•+^-COF are −721, −951 and −953 meV, respectively, indicating the strong interaction of R-TTF^•+^-COF with LiPSs. This trend also suggests a higher capability of R-TTF^•+^-COF in immobilizing the lower-order LiPSs, i.e., Li_2_S_6_ and Li_2_S_4_. Compared with those in the gas phase, the S–S bonds of Li_2_S_8_, Li_2_S_6,_ and Li_2_S_4_ are elongated by a maximum of 18.4%, 5.9%, and 1.3% when adsorbed on R-TTF^•+^-COF, respectively (Figure 6a). The free energies for the lithiation reactions of polysulfide intermediates were calculated with R-TTF^•+^-COF and in the gas phase (Figure 6a). The reaction energies with R-TTF^•+^-COF are sizably lower for the reduction of Li_2_S_8_, Li_2_S_6_, and Li_2_S_4_. These results further demonstrate the catalytic activity of R-TTF^•+^-COF in the SRR, thus ensuring an overall improved electrochemical performance of Li–S cells.

**Figure 6:**
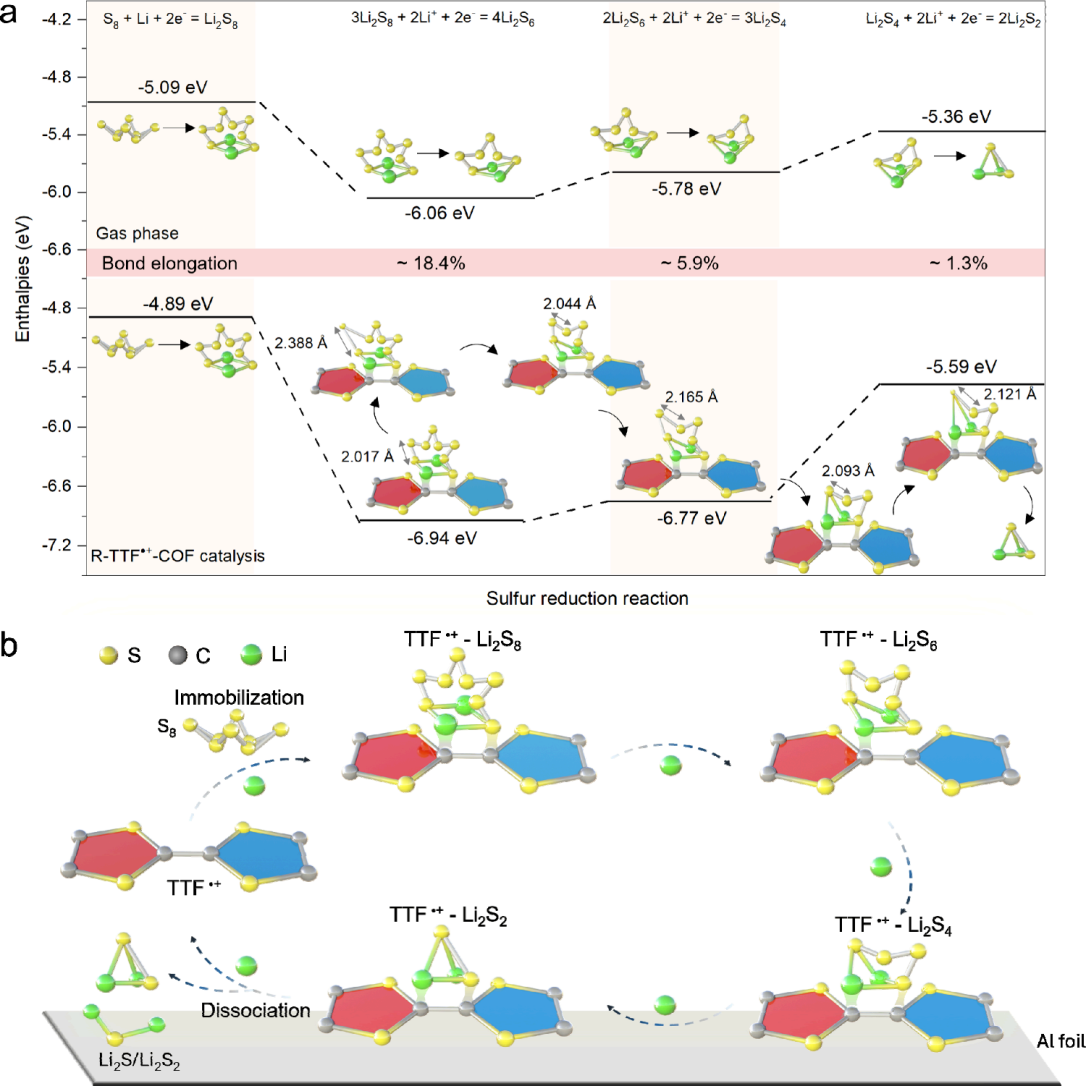
Computational study on the catalytic effect of R-TTF^•+^-COF toward LiPS conversion and the proposed catalytic mechanism. (a) Gibbs free energy of SRR from S_8_ to Li_2_S_2_ in the gas phase or in the presence of R-TTF^•+^-COF, and evolution of bond lengths of Li_2_S_8_, Li_2_S_6_, and Li_2_S_4_ in the presence of R-TTF^•+^-COF during SRR. (b) The proposed catalytic mechanism to promote SRR via the covalent fixation of LiPSs on R-TTF^•+^-COF, thereby facilitating the cleavage of S–S bonds (sulfur atoms, yellow; carbon atoms, gray; lithium atoms, green).

The herein proposed catalytic mechanism of R-TTF^•+^-COF in Li–S batteries is based on previous reports of [TTF]_2_^•+^ TFSI^–^ as illustrated in Figure 6b. As electrocatalysts in the sulfur cathode, R-TTF^•+^-COF with active radical-cationic groups [TTF]_2_^•+^ are both electrophilic and nucleophilic.^24^ Profiting from this, LiPSs can be grafted via electron-rich radical sites with Li and electron-deficient sites with S in [TTF]_2_^•+^ units, owing to the different electron affinities of Li and S atoms.^60^ Therefore, LiPSs are reversibly anchored to the radical-cationic sites and meanwhile transformed to lower-order lithium sulfides via accelerating the cleavage of long-chain LiPSs, which thus facilitates the SRR kinetics and effectively suppresses the shuttle effects during the electrochemical process.

## Conclusions

In summary, the one-step sulfurization of an imine-linked TTF-COF creates a R-TTF^•+^-COF composed of benzothiazole linkages and radical-cationic [TTF]_2_^•+^ motifs, which presents an extended conjugated framework with superior electrical conductivity of 3.9 S m^–1^ at room temperature. Consequently, the R-TTF^•+^-COF exhibits an improved interlayer electron mobility, enhanced adsorption capability, and efficient catalytic activity for polysulfide conversion. The reported R-TTF^•+^-COF-based Li–S batteries achieve superior long-term cycling stability for 1500 cycles at 0.5 C with just 0.027% capacity loss per cycle and significantly improved rate capability. The radical-cationic sites in R-TTF^•+^-COF provide both electrophilic and nucleophilic sites to dynamically fix LiPSs and effectively promote the cleavage of S–S bonds in the SRR. Detailed DFT computations reveal the strong interactions between R-TTF^•+^-COF and LiPSs, along with the catalytic effect of R-TTF^•+^-COF in the SRR via prolonging the S–S bonds and lowering the enthalpies of SRR from Li_2_S_8_ to Li_2_S_2_. Furthermore, the coordinating anions within R-TTF^•+^-COF show no influence on either the bandgap or the catalytic activity toward LiPSs, thereby underscoring the role of the radical cations [TTF]_2_^•+^ as the primary catalytic centers. This study paves the way for the exploration and design of conductive and stable open-shell radical COF materials and advanced organic electrocatalysts for Li–S batteries and other energy storage applications.

## Supplementary Material



## Notes

Additional experimental details, materials, and methods (PDF)

## a

School of Chemistry and Molecular Engineering, Nanjing Tech University, 211816 Nanjing, China
