# Supplementary material for: A Radical-Cationic Covalent Organic Framework to Accelerate Polysulfide Conversion for Long-Durable Lithium–Sulfur Batteries
Source: J Am Chem Soc. 2025 Aug 19;147(34):31073–84. doi: 10.1021/jacs.5c09421 (PMC12395410; doi:10.1021/jacs.5c09421)
Supplement: Supplementary file 1 [file ja5c09421_si_001.pdf]

A Radical-Cationic Covalent Organic Framework to Accelerate Polysulfide Conversion for Long-Durable Lithium-Sulfur Batteries

Sijia Cao, Pouya Partovi-Azar, Jin Yang, Dongjiu Xie, Timo Held, Gianluca Marcozzi, Joseph E. McPeak, Wei Zhang, Xia Zhang, Markus Osenberg, Zdravko Kochovski, Changxia Li, Daniel Sebastiani, Johannes Schmidt, Moritz Exner, Ingo Manke, Arne Thomas*, Wenxi Wang*, Yan Lu*.

Contents

1	Materials and instrumentations
2	Experimental methods
3	Structural characterizations of COFs
4	Kinetics study of COFs to catalyze polysulfide conversion
5	Electrochemical measurements of Li-S batteries
6	Analysis of discharged and charged samples to study the catalytic mechanism
7	Computational studies to understand the catalytic mechanism

1. Materials and instrumentations

All reagents and solvents are commercially obtained and used as received. 4,4',4'',4'''-([2,2'-Bi(1,3-dithiolylidene)]-4,4',5,5'-tetrayl)tetrabenzaldehyde (97%) was purchased from BLDpharm. Sublimed sulfur powder (200 mesh), lithium nitrate ($LiNO_3$), bis(trifluoromethane)sulfonimide lithium salt (LiTFSI), polyvinylidene fluoride (PVDF), *N*-methyl-2-pyrrolidone (NMP, ≥ 99%), *p*-phenylenediamine (97%), 1,4-dioxolane (99.8%), mesitylene (≥ 99.8%), 1,2-dimethoxyethane (DME, 99.9%), and 1,3-dioxolane (DOL, 99.9%) were purchased from Sigma-Aldrich.

Elemental analyses were performed on a Thermo FlashEA 1112 Organic Elemental Analyzer. Each measurement was carried out parallelly three times and the results were taken as the average of three values.

Powder X-ray diffraction (PXRD) measurements were performed on a Bruker D8 Advance instrument with Cu Kα radiation (λ=1.54 Å) at a generator voltage of 40 kV and generator current of 40 mA (scanning speed, 2° min^{-1}; scanning range from 2° to 60°).

Nitrogen sorption measurements were carried out on a Quantachrome Quadrasorb SI instrument. The specific surface area was calculated using the Brunauer-Emmett-Teller (BET) method and pore size distribution (PSD) was calculated from the adsorption branch of isotherms using the non-local density functional theory (NLDFT) model.

Transmission electron microscopy (TEM) was performed with a low dose acquisition scheme using SerialEM[1] on JEOL JEM-2100 (JEOL GmbH, Eching, Germany) operated at 200 kV and equipped with a 4 k × 4 k CMOS digital camera (TVIPS TemCam-F416). HRTEM images were acquired at a magnification of 500,000×, corresponding to a pixel size of 0.23 Å at the specimen level, while keeping the total electron dose below 10 e $Å^{-2}$. All imaging was carried out at temperatures around 90 K. COF samples for TEM were sonicated in methanol for 5 min and a 5 μL of sample dispersion was applied to Lacey carbon-coated copper TEM grids (200 mesh, Science Services) and subsequently dried under a

fume hood. The grids were loaded at room temperature into a cryogenic transfer holder (Gatan 914, Gatan, Munich, Germany) that was cooled with liquid nitrogen after the transfer into the TEM column.

X-ray photoelectron spectra (XPS) were conducted on a Thermo Scientific K-Alpha+ X-ray Photoelectron Spectrometer with Al Kα radiation. The samples were pressed onto the sample holder using carbon tape for measurement. The air-sensitive sample was prepared under an argon atmosphere in the glovebox and transferred in the argon-sealed holder.

Solid-state diffuse reflectance Ultraviolet-visible-near-infrared spectroscopy (UV-vis-NIR/DRS) analysis has been done on Agilent Cary 4000 UV-Vis Spectrophotometer with wavelength range 200-1400 nm (± 0.08 nm accuracy).

Scanning electron microscopy (SEM) analyses were performed on an electron microscope Zeiss Merlin. The accelerating voltages are 3-5 kV for images. The pristine COF samples were coated with a thin gold layer to improve the electrical conductivity for SEM measurement. Energy dispersive X-ray spectrometry (EDX) was performed with Brucker QUANTAX detector.

Fourier transform infrared spectra (FTIR) were conducted on a Thermo Nicolet Magna 750 FTIR Spectrometer with KBr-Pressing for powder samples. Air-sensitive FTIR analyses were performed on a Jasco Spectrometer in ATR mode in the Ar-filled glovebox.

^{13}C cross-polarization magic-angle spinning (CP/MAS) solid-state nuclear magnetic resonance (ssNMR) spectra were carried out on a Bruker AVIII HD 400WB spectrometer equipped with a 4 mm Low Gamma Magic Angle Spinning Probe operating at 100.6 MHz. ^{7}Li ssNMR spectra were performed at 155.4 MHz.

Electron paramagnetic resonance (EPR) spectra were collected on the Miniscope MS5000 benchtop system at X-band (~ 9.5 GHz) at room temperature. The intensity is normalized by the mass of each sample.

Thermogravimetric analysis (TGA) was performed using a Netzsch TG209 F1 analyzer under a nitrogen atmosphere, with a heating rate of 10 °C/min, over a temperature range of 25–800 °C. TG-mass spectrometry (TG-MS) analysis was conducted by coupling the TGA with a Netzsch QMS 403 C mass spectrometer.

Ex-situ and *operando* Raman spectroscopy were both conducted with the Renishaw InVia confocal Raman microscope with a 532 nm laser at 0.1% of its maximum power. *Operando* spectroscopy was performed using an EL-Cell ECC Opto-10 cell holder, where a two-electrode side-by-side configuration featuring a sulfur electrode stripe and a metallic counter electrode stripe was utilized. The cells were cycled at a rate of 0.05 C.

Four-probe conductivity measurements: the specific resistivity and conductivity of COF pellets (~ 5 mm in diameter) were measured following compression using a hollow-cylinder punch press at a pressure of ~ 1.5 tons, within a custom-built setup. During compression, electrical resistivity was recorded between the two electrodes. The specific resistivity was calculated by multiplying the measured resistance by the cross-sectional area and dividing by the pellet thickness. Conductivity was then obtained as the reciprocal of the specific resistivity.

X-ray tomography measurements were implemented with a cell with a configuration of Li (2.5 μm in diameter) | Separator (3 μm in diameter) | COF/S cathode (2.5 μm in diameter), as shown in Supplementary Fig.32. Synchrotron X-ray tomography was conducted at the BAMline within BESSY II in Berlin, Germany. The synchrotron beam was monochromatized to 25 keV using a double monochromator, which achieved an energy resolution of approximately 1.5%. For the tomography measurements, 2400 projections were recorded over a 180° battery rotation, each with an exposure time of 0.3 s. To expedite measurements due to limited beam time allocation, a 2 by 2 binning technique was employed. This reduced exposure time and resulted in a total measurement duration of approximately 20 minutes, yielding a spatial resolution of 0.88 μm. The raw tomography data obtained from BAMline underwent processing using in-house reconstruction software developed in IDL 8.2. The data was first normalized and de-noised, with additional filtering and phase retrieval where

necessary. Subsequently, filtered back projection was employed for the final reconstruction. 3D visualizations presented in this context were created using VG Studio MAX 3.1.

2. Experimental methods

2.1 The Synthesis of TTF-COF

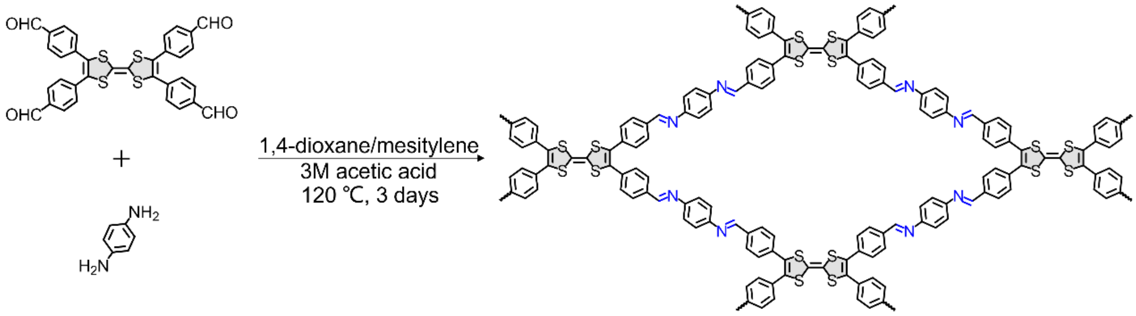

Scheme S1 Schematic synthesis of TTF-COF.

32.7 mg 4,4',4'',4'''-([2,2'-Bi(1,3-dithiolylidene)]-4,4',5,5'-tetrayl)tetrabenzaldehyde (TTF) and 12.9 mg *p*-phenylenediamine (Ph) were added into a Pyrex tube with 2.4 mL mixed solvent (1,4-dioxane/mesitylene/3 M acetic acid = 1:1:0.4 (v/v/v)). The tube was sonicated for 20 min to guarantee a uniform suspension. Subsequently, the tube was frozen in liquid nitrogen, evacuated to 0.05 mbar, and allowed to thaw in order to complete three consecutive freeze-pump-thaw cycles. Afterwards, the tube was flame-sealed and placed in the oven at 120 °C for 3 days. The tube was then cooled down to room temperature (RT). The resulting precipitate was washed thoroughly three times using 10 mL chloroform, 10 mL tetrahydrofuran and 10 mL acetone 3 times, sequentially. The obtained powder was then dried at 120 °C overnight under vacuum. The final reddish-brown powder was obtained with a yield of ~75%, termed as TTF-COF. (Elemental analysis: calculated (%): C:72.25, N:7.33, S:16.76, H:3.66; found (%): C:70.08, N:7.11, S:16.12, H:3.79).

2.2 The Synthesis of R-TTF$^{\bullet+}$-COF

R-TTF$^{\bullet+}$-COF was prepared by mixing TTF-COF powder and sulfur powder (mass ratio=1:15), followed by grinding for 15 min and dispersing in the toluene (toluene: S_8 = 50:1 wt.%) by sonication for 2 h. The solvent was removed via vacuum filtration. The mixture was dried overnight under 40 °C and then transferred to a Pyrex tube. The tube was frozen in liquid nitrogen, evacuated to 0.05 mbar, flame-sealed, and positioned in the furnace at 130 °C for 2 h with a heating rate of 1 °C min^{-1}. The temperature was increased up to 155 °C and held for 5 h (heating rate = 1 °C min^{-1}) and ultimately increased to 360 °C and held for 5 h (heating rate = 1 °C min^{-1}), finally cooled down to RT. The obtained powder was thoroughly washed using Soxhlet extraction with toluene overnight and then dried in the vacuum oven at 120 °C overnight. The obtained dark-brown powder was denoted as R-TTF$^{\bullet+}$-COF (360 °C_5 h). To investigate the effects of temperature and reaction time, the reaction temperature was varied to 200 °C and 300 °C, yielding R-TTF$^{\bullet+}$-COF (200 °C_5 h) and R-TTF$^{\bullet+}$-COF (300 °C_5 h), respectively. Additionally, the reaction conducted at 360 °C was shortened to 2 hours, resulting in R-TTF$^{\bullet+}$-COF (360 °C_2 h).

2.3 The preparation of COF/S composites

TTF-COF powder and S_8 were mixed at a weight ratio of 1:3, and then ground for 15 min and sonicated for 2 h in the toluene. The solvent was removed via vacuum filtration. The mixture was transferred to a Pyrex tube after drying under vacuum at 40 °C. The tube was frozen in liquid nitrogen, evacuated to 0.05 mbar, flame-sealed, and positioned in the furnace at 130 °C for 2 h with a heating rate of 1 °C min^{-1}. Afterwards, the temperature was increased to 155 °C and maintained for 5 h (heating rate = 1 °C min^{-1}) and then increased to 360 °C and remained for 5 h (heating rate = 1 °C min^{-1}). The composites were denoted as R-TTF$^{\bullet+}$-COF/S. As a reference, the TTF-COF/S_8 mixture was transferred to a Teflon container and sealed in a steel autoclave under an argon atmosphere in a glove box. The autoclave was heated in the oven at 155 °C for 12 h. The resulting composites were denoted as TTF-COF/S.

2.4 Electrochemical tests

The COF/S composites were mixed with super P and polyvinylidene fluoride (PVDF) in a weight ratio of 7:2:1 in the N-Methyl-2-Pyrrolidone (NMP) solvent to prepare the uniform slurry. The slurry was coated onto carbon-coated aluminum foil using a doctor blade with a wet film thickness of 150 micrometers and dried in the oven at 50 °C for 12 h. The electrode was then cut into round slices with a diameter of ~12 mm as cathodes. The areal loading of active materials is ~1.1-1.3 mg cm^{-2}. The cathodes with high sulfur loading were prepared by adjusting the electrode thickness. The coin cells were assembled with the cathode, a Li chip as the anode, and a piece of Celgard 2500 membrane as the separator in an Ar-filled glovebox (UNIlab plus, M. BRAUN) with H_2O content <0.5 ppm and O_2 content <0.5 ppm. 1 M LiTFSI dissolved in 1,2-dimethoxyethane (DME)/1,3-dioxolane (DOL) (v/v =1:1) with 2 wt% $LiNO_3$ was prepared as the electrolyte. The current density of 1 C equals 1675 mA g^{-1}. The specific capacity was calculated based on the mass of sulfur content in the electrode. Galvanostatic (dis)charging tests were performed on a Neware battery testing system (CT-4008-5 V10 mA) at room temperature. The electrochemical impedance spectroscopy (EIS) was recorded using a GAMRY Interface 1000 within a frequency range of 1000 kHz to 0.01 Hz. The cyclic voltammetry (CV) was conducted with the GAMRY Interface 1000 by varying scan rates in the electrochemical window of 1.7–2.8 V *vs* Li/Li^+. To carry out the shuttle current measurement, the coin cells were cycled for two rounds of (dis)charge at 0.05C rate to stabilize the cells. In the third cycle, the cells were fully discharged and then charged to 2.7 V, and allowed to rest for 10 min. Afterwards, the cells were potentiostatically held at 2.35 V for 10 h. A Li_2S_6 solution was prepared by dissolving stoichiometric sulfur and Li_2S (5:1, in molar ratio) in a mixed solvent of DOL/DME (1:1, v/v), followed by vigorous stirring at 80 °C for 48 h. A Li_2S_8 solution was prepared using the same procedure with sulfur and Li_2S in a 7:1 molar ratio. For the polysulfide conversion tests, the slurry of the host materials (TTF-COF/R-$TTF^{\bullet+}$-COF), super P, and PVDF (70:20:10 wt.%) in NMP was coated on carbon paper. After drying at 50 °C overnight, the electrode was cut into slices with a diameter of ~12 mm. The areal loading of the host materials was ~ 0.8 mg cm^{-2}. Two identical electrodes were used to assemble the symmetric cells. A 40 μL 0.1 M Li_2S_6 solution with 1.0 M LiTFSI dissolved in DOL/DME (1:1, v/v) solvent with 2 wt% $LiNO_3$ was used as electrolyte. The symmetric cells were measured at a scan rate of 5.0 mV s^{-1}

in a potential window of -0.8–0.8 V. The same electrodes were used in Li_2S precipitation tests. The cells were assembled using 20 μL 0.25 M Li_2S_8 solution with 1.0 M LiTFSI in DOL/DME (1:1, v/v) solvent with 2 wt% $LiNO_3$ as catholyte, and 20 μL electrolyte without Li_2S_8 was used as anolyte. The cells were first galvanostatically discharged at 0.1 C to 2.16 V and then potentiostatically held at 2.05 V for Li_2S nucleation and growth. The potentiostatic discharge was terminated after 65000 s. To carry out the second precipitation, the cell was galvanostatically charged at 0.1 C to 2.8 V after the first Li_2S precipitation, then galvanostatically discharged at 0.1 C to 2.16 V and potentiostatically discharged at 2.05 V for the second Li_2S electrodeposition.

2.5 Computational details

All calculations have been performed at the density functional theory (DFT)[2] level using the CP2K software package[3]. The DFT calculations have been carried out in conjunction with a basis set with triple-zeta valence plus one set of polarization functions for hydrogen, carbon, oxygen, nitrogen, and sulfur (TZVP-MOLOPT) together with a double-zeta valence plus one set of polarisation functions (DZVP-MOLOPT) for lithium. Both basis sets are optimized from molecular calculations[4]. We have also used Goedecker-Teter-Hutter (GTH) pseudopotentials in all calculations[5,6], together with the semiempirical DFT-D3[7] correct for the long-range dispersion interactions.

In the first step, to account for the exchange and correlation effects, Perdew-Burke-Ernzerhof (PBE)[8] generalised gradient approximation has been used for the calculations involving both atomic coordinates and unit cell optimisations. In these calculations, the external pressure has been set to 0.0 GPa while the pressure tolerance and maximum atomic force component have been set to 0.01 GPa and 0.02 eV/Å, respectively.

In order to have a more reliable treatment of possible charge localization and radicals, the optimised coordinates and unit cells have been used for a further optimization of atomic coordinates and electronic structure calculations using the PBE0 hybrid functional[9]. In DFT calculations with hybrid exchange-correlation functionals, a truncated Coulomb operator is considered in CP2K for the exchange part[10]. The parameter for Schwarz screening has been set to 10^{-6}. We have also employed the auxiliary density matrix method[11] to lower the computational cost of periodic hybrid DFT calculations. A pFIT3 basis set has been used for these calculations.

First, the structure of TTF-COF has been fully optimized (i.e. atomic coordinates together with the unit cell) with starting unit cell vectors of length $|a|$=24.800Å, $|b|$=24.900Å, $|c|$=7.420Å, and angles α=90.000, β=90.000, and γ=60.000. Afterward, the structure of R-TTF$^{\bullet+}$-COF was fully optimized, starting from the optimized coordinates and the unit cell from the first step. In these computations, two COF layers have been considered inside a unit cell.

To explore the possibility of covalently bonded sulfur chains in R-TTF$^{\bullet+}$-COF structures, various starting structures have been considered, where sulfur chains Sx of different lengths ($x = 1...8$) were assumed to be initially attached to distinct positions in R-TTF$^{\bullet+}$-COF. In these optimizations, only the atomic coordinates have been optimized.

In computations of Gibbs free energy, the entropic contributions to the free energy of each system are assumed to be negligible compared to its internal energy. It is worth mentioning here that similar results were obtained by considering the formation of other LiPS products in each reaction. Moreover, the volume change during the reactions was considered to be insignificant. Therefore, the Gibbs free energy for each system is approximated by its internal energy.

3. Structural characterizations of COFs

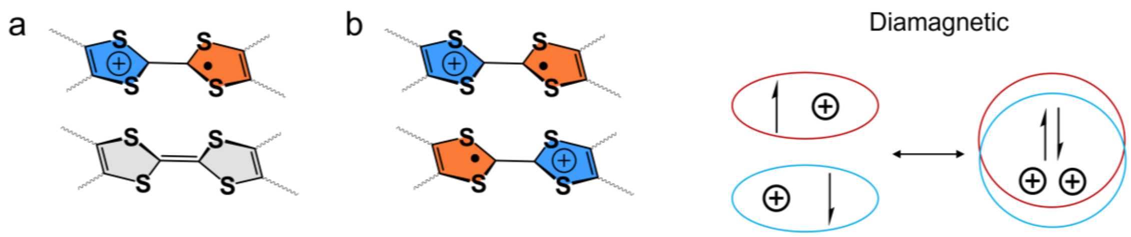

Scheme S2 (a) The structure of the mixed-valence complex $[TTF]_2^{\bullet+}$. (b) The structure of dimers $[TTF^{\bullet+}]_2$ and the diamagnetic state resulting in the silent EPR signal. Oxidation of closely stacked TTF can generate two possible radical-cations, which are mixed-valence complex $[TTF]_2^{\bullet+}$ and dimers $[TTF^{\bullet+}]_2$, respectively. The former is paramagnetic while the latter features a diamagnetic dimeric EPR-

silent structure.[12,13]

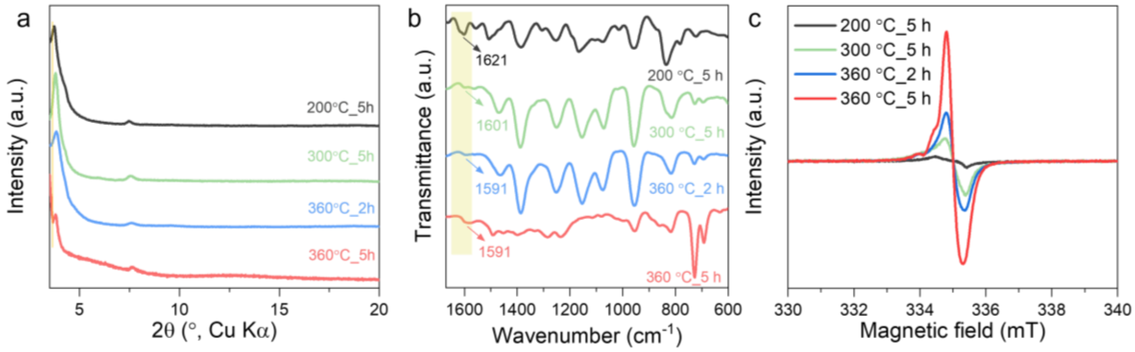

Figure S1 (a) PXRD (b) FTIR spectra (c) Solid-state EPR spectra of post-sulfurized TTF-COF at 200 °C, 300 °C, 360 °C for 2 hours and 5 hours. To further investigate the optimization process, we examined the products synthesized at different temperatures — 200 °C, 300 °C, and 360 °C. The crystal structures of the resulting materials were analyzed using powder X-ray diffraction (PXRD). The (110) diffraction peaks show a gradual shift toward higher angles with increasing temperature: from 3.72° at 200 °C (5 h), to 3.81° at 300 °C (5 h), and 3.86° at 360 °C (5 h), indicating a temperature-induced transformation of the pore structure. In the FTIR spectra, the characteristic imine bond absorption at 1621 cm^{-1} remains unchanged at 200 °C, but shifts to 1601 cm^{-1} at 300 °C and 1591 cm^{-1} at 360 °C. This suggests partial oxidation of the imine bonds at 300 °C and their complete conversion to benzothiazole linkages at 360 °C. Furthermore, electron paramagnetic resonance (EPR) spectroscopy reveals a significantly higher radical concentration in the sample treated at 360 °C, compared to those treated at 200 °C and 300 °C. The elemental analysis results (**Table S1**) show that there is no sulfur chain incorporation in the sample prepared at 200 °C, in agreement with the negligible intensity in EPR spectrum. And the increase in the temperature increases the sulfur dosage, which can be attributed to the oxidation degree of the TTF moieties, which support the EPR results very well. We also investigated the effect of reaction time at 360 °C by comparing products obtained after 2 hours and 5 hours. The (110) reflection remains at 3.86° for both durations, and the FTIR bands corresponding to benzothiazole are consistently observed at 1591 cm^{-1}, indicating that the imine-to-benzothiazole transformation occurs within 2 hours. However, the concentration of $TTF^{\bullet +}$ radicals in the 5-hour sample is approximately twice that of the 2-hour sample. The elemental analysis results show that the longer duration (5 h) increases the sulfur dosage, which is consistent with the higher oxidation degree of the TTF moieties shown in the EPR spectra.

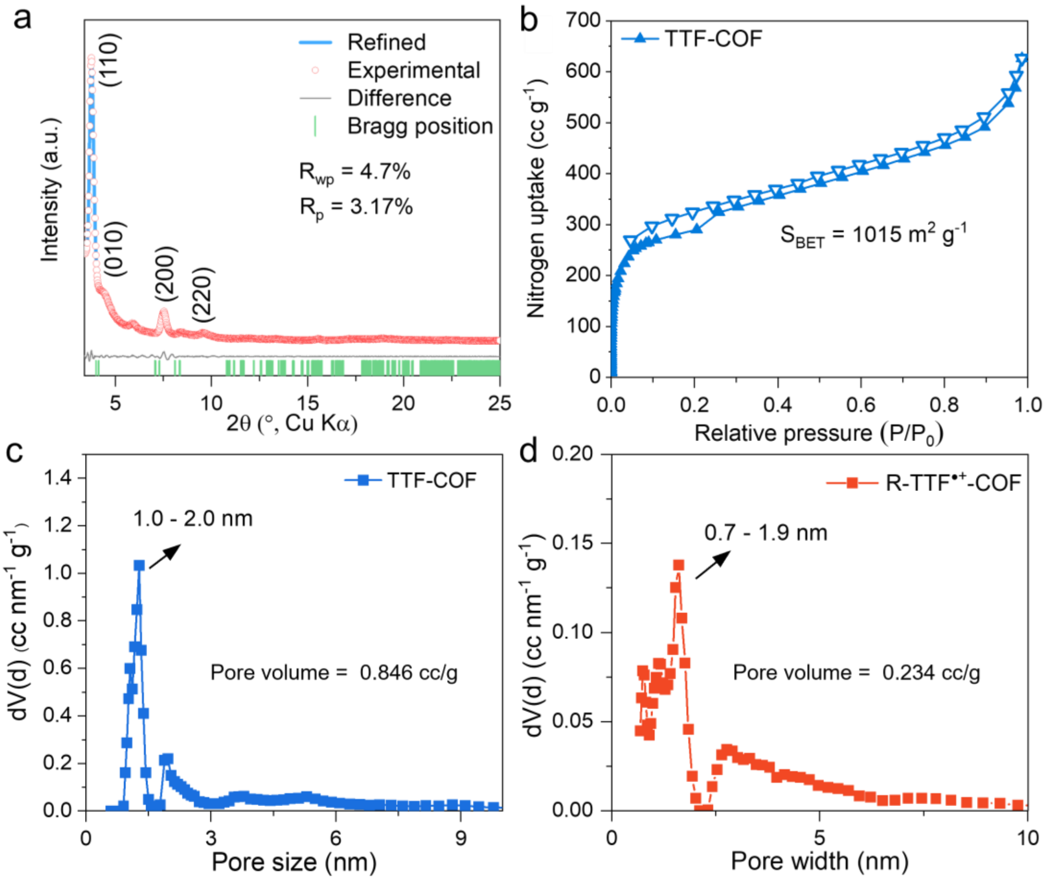

Figure S2 (a) The experimental PXRD patterns and the simulated XRD results of TTF-COF. The crystal structure for TTF-COF was simulated by crystal building and geometry optimization based on DFT calculation. Pawley refinement was used to further fit the experimentally obtained structure for TTF-COF using Material Studio V 5.0. Based on a *C1* monoclinic space group with lattice parameters of a=24.775, b=24.052, c=8.145 Å, and α = 90.723, β=90.969, and γ= 61.054°, a simulated XRD pattern (blue curve) is in good agreement with the experimentally observed pattern, showing their negligible difference (grey curve). Structural simulations indicate that the pore width of the TTF-COF is 2.1 nm, whereas that of the R-TTF$^{•+}$-COF is reduced to 1.8 nm. (b) Nitrogen adsorption-desorption isotherms of TTF-COF measured at 77 K. TTF-COF display type I isotherms, showing the microporous nature. The Brunauer–Emmett–Teller surface area (S_{BET}) of TTF-COF is 1015 $m^2 g^{-1}$. (c) Pore size distributions of TTF-COF. (d) Pore size distributions of R-TTF$^{•+}$-COF.

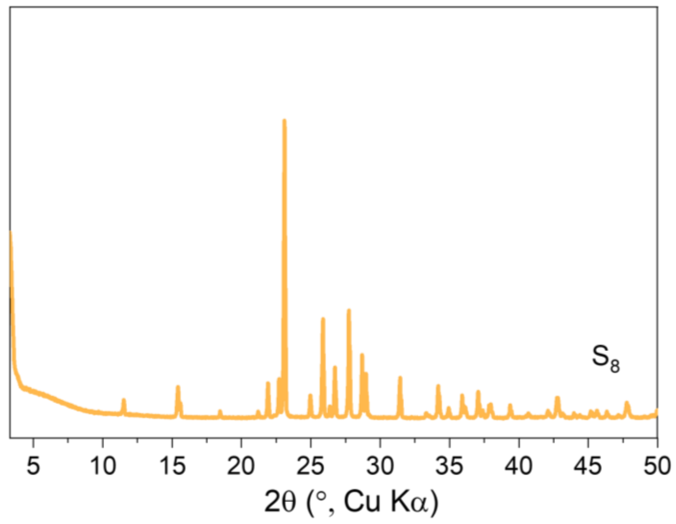

Figure S3 PXRD pattern of S_8.

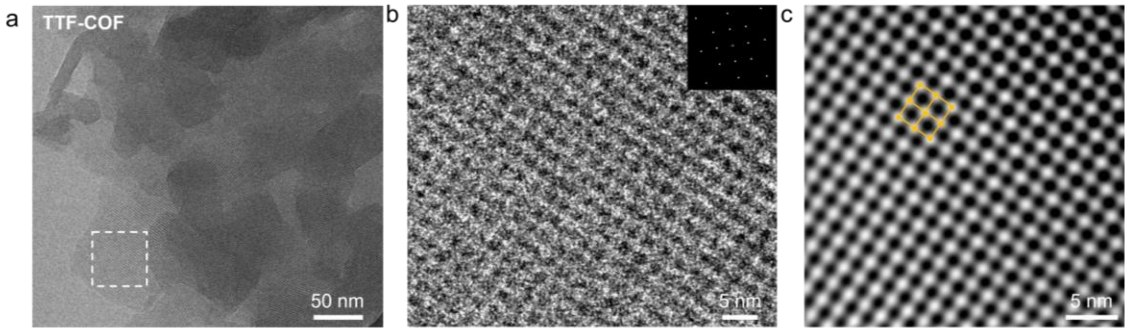

Figure S4 (a) Cryogenic low-dose TEM image of TTF-COF and FFT (inset). (b) Cryogenic low-dose HRTEM image of the region indicated by the white square in (a) with FFT (inset). (c) Inverse FFT of (b).

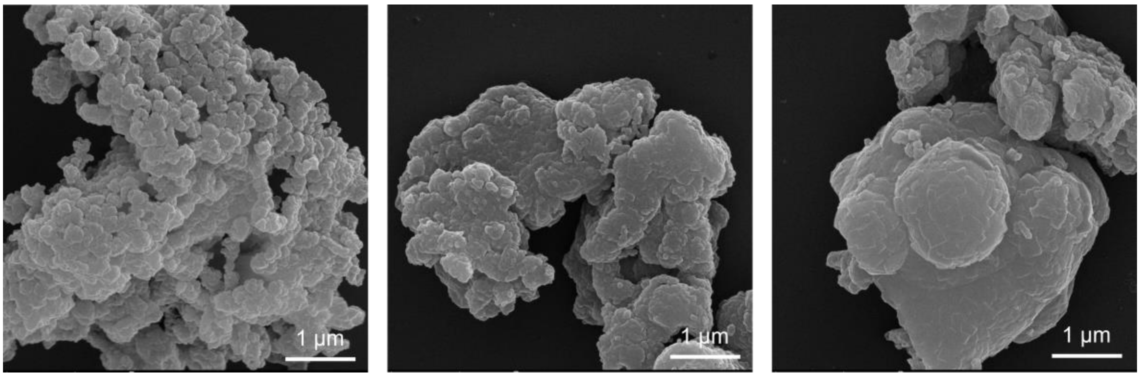

Figure S5 SEM images of TTF-COF powder.

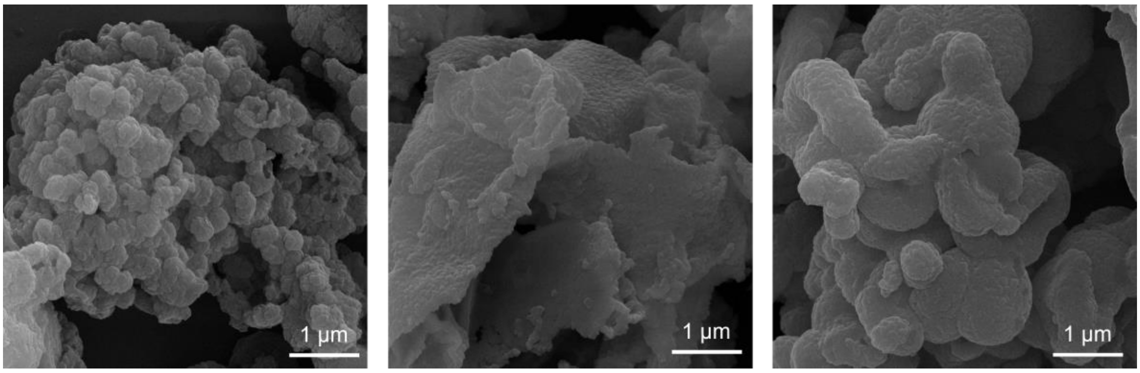

Figure S6 SEM images of R-TTF$^{•+}$-COF powder.

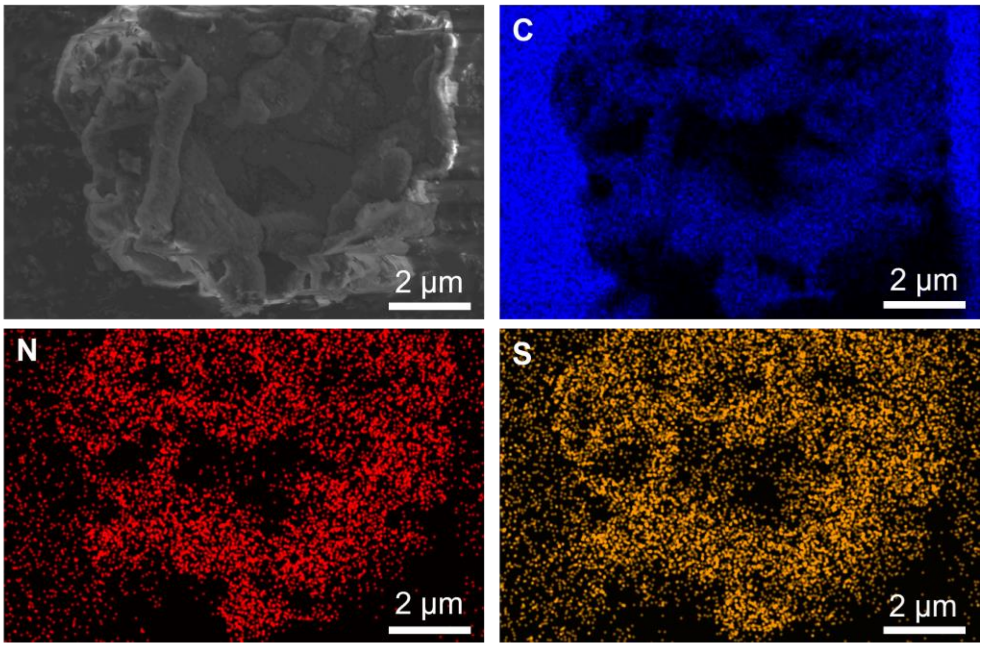

Figure S7 SEM images of R-TTF$^{\bullet+}$-COF powder and the corresponding EDX mapping of elements C, N, S, respectively.

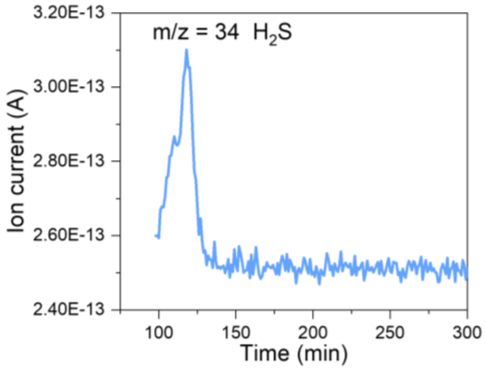

Figure S8 Time-dependent abundance of the H_2S fragment from the mass spectrum of *in situ* reaction at 360 °C. The TTF-COF/S mixture was first treated at 155 °C for 5 hours in a sealed autoclave under Argon atmosphere. Subsequently, the treated composites were transferred to the chamber and kept at 360 °C for 5 hours under Argon flow for TG-MS analysis. There appears a sharp ion current with m/z value of 34 at the reaction time of 115 min, which can be assigned as H_2S species. The formation of H_2S suggests the transformation of the imine-bonds in TTF-COF into the benzothiazole structure in 2 hours, which is also evidenced by presence of benzothiazole in the FTIR spectrum of R-TTF$^{\bullet+}$-COF

(360 °C_2 h).

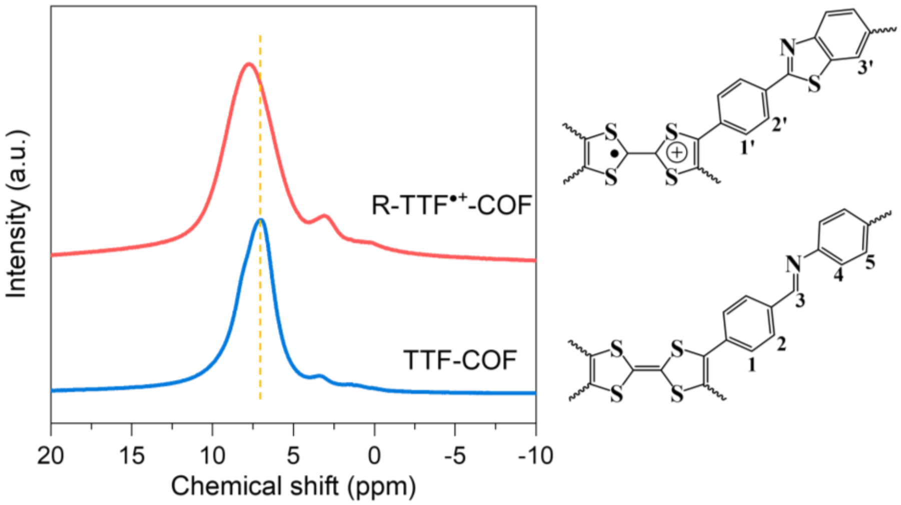

Figure S9 Solid-state ^{1}H NMR spectra of TTF-COF and R-TTF$^{\bullet+}$-COF. Compared to TTF-COF, the spectrum of R-TTF$^{\bullet+}$-COF shows an overall shift to a higher frequency, indicating the deshielding phenomenon, in according with the oxidation of TTF-COF.

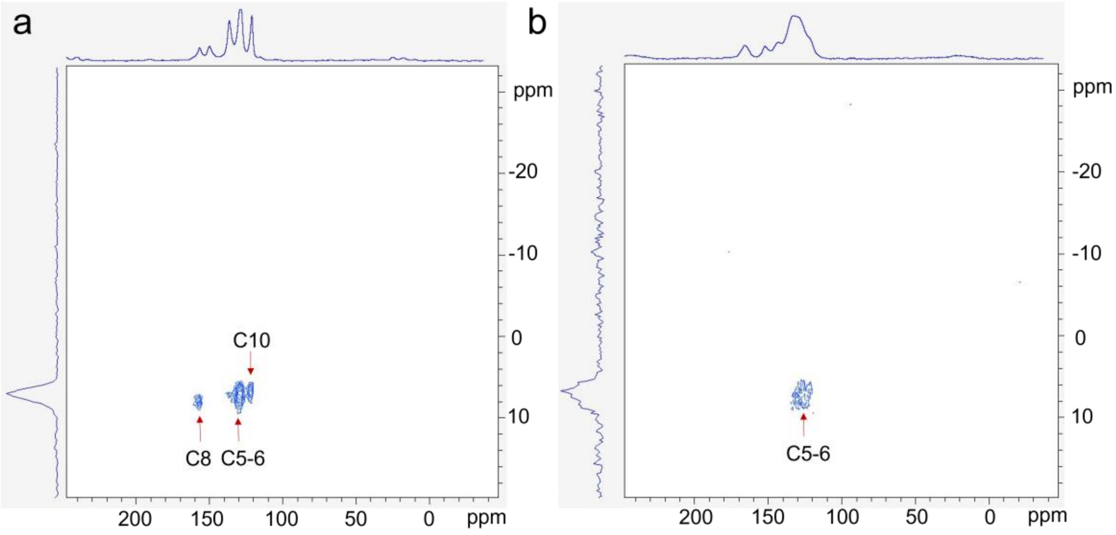

Figure S10 Solid-state 2D ^{1}H-^{13}C HETCOR NMR spectra of (a) TTF-COF and (b) R-TTF$^{\bullet+}$-COF. The chemical shift of ^{13}C is shown on the horizontal axis and the chemical shift of ^{1}H is shown on the

vertical axis.

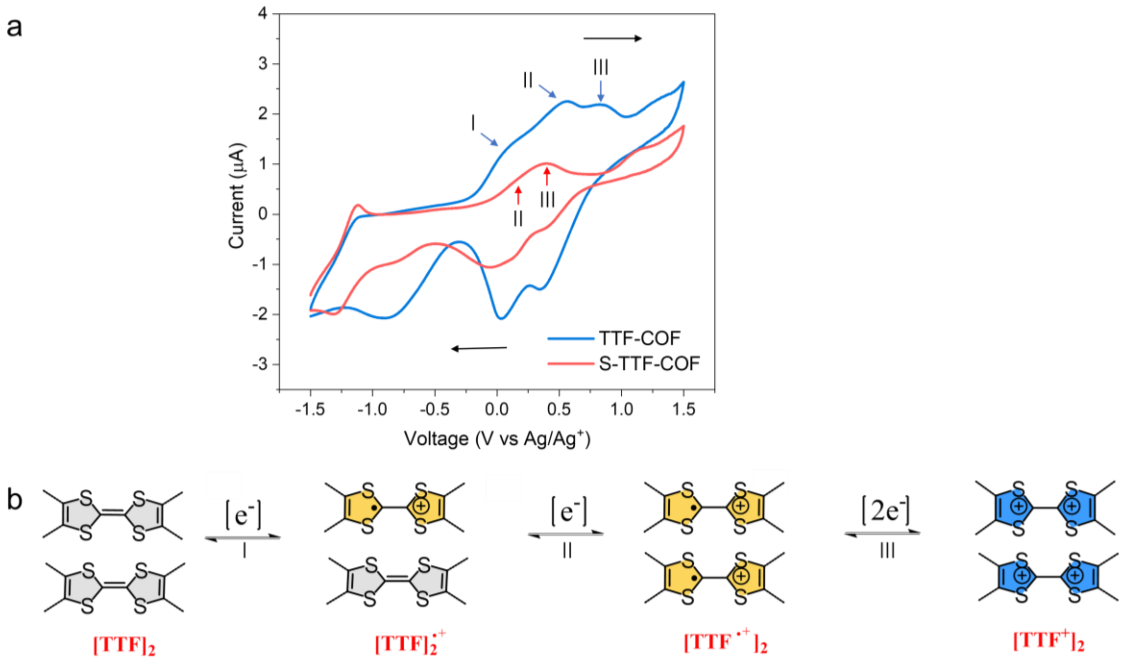

Figure S11 (a) Cyclic voltammetry curves of TTF-COF and R-TTF$^{\bullet+}$-COF at a scan rate of 10 mV s^{-1} in a three-electrode system. The COF powder was mixed with 10% Super P in NMP to prepare the uniform "ink". The "ink" coated on carbon paper was used as the working electrode, Pt foil was used as the counter electrode (CE), and non-aqueous Ag/Ag^+ was used as the reference electrode (RE). The cyclic voltammograms were acquired by scanning from -1.5 to +1.5 V in the electrolyte consisting of 0.1 M tetrabutylammonium hexafluorophosphate (TBAPF$_4$) dissolved in CH_3CN solution. (b) The four-electron redox reactions of bilayer stacked TTF groups. The stacked non-aromatic [TTF]$_2$ groups in TTF-COF are first oxidized to [TTF]$_2^{\bullet+}$ at 74 mV (I), transformed to the [TTF$^{\bullet+}$]$_2$ dimers at 560 mV (II), and then to dicationic [TTF$^+$]$_2$ at 837 mV (III). In contrast, transformation II of R-TTF$^{\bullet+}$-COF occurs at 116 mV, followed by transformation III at 404 mV. The potentials are negatively shifted compared to those of TTF-COF, which is probably attributed to the oxidized backbones of R-TTF$^{\bullet+}$-COF.[14-16]

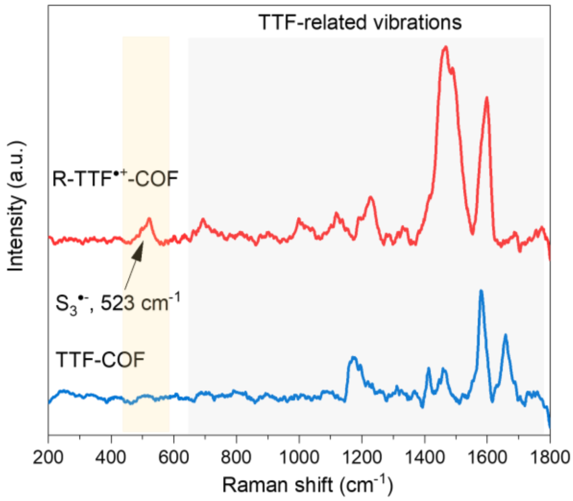

Figure S12 Raman spectra for TTF-COF and R-TTF$^{•+}$-COF. The Raman signals in the 700-1800 cm^{-1} range belong to the TTF groups.[17-19] The emergence of the peak at 523 cm^{-1} in R-TTF$^{•+}$-COF is assigned to $S_3^{•-}$.

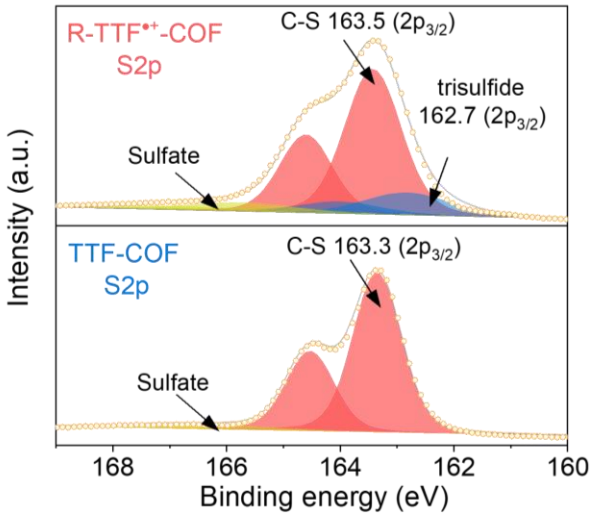

Figure S13 High-resolution S 2p XPS spectra for TTF-COF and R-TTF$^{•+}$-COF.

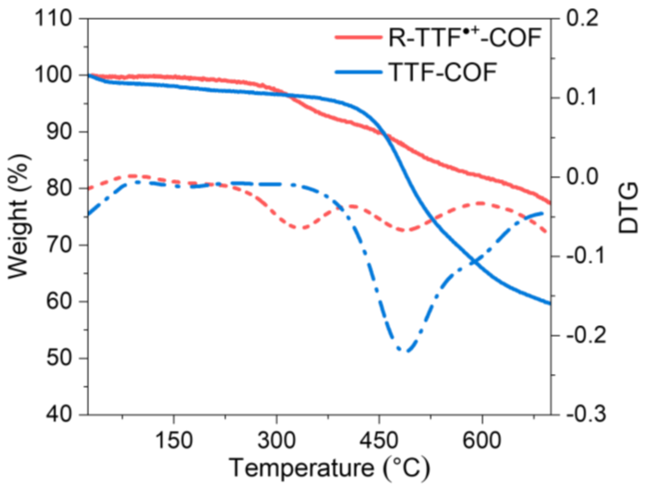

Figure S14 TGA (solid line) and DTA data (dashed line) for TTF-COF (blue) and R-TTF$^{\bullet+}$-COF (red), respectively. R-TTF$^{\bullet+}$-COF shows lower thermal stability with two-stage decomposition at 295 °C and 420 °C, respectively. The weight loss in the first stage comes from the incorporated sulfur species in R-TTF$^{\bullet+}$-COF. For comparison, TTF-COF shows only one-step decomposition starting from 410 °C, attributed to the degradation of the main COF skeleton.

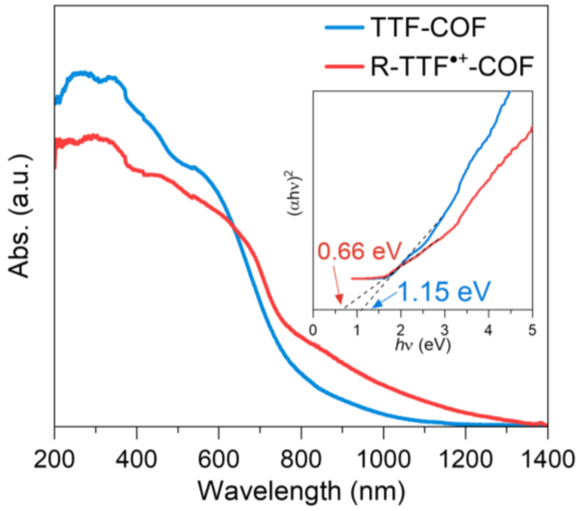

Figure S15 UV-vis-NIR/DRS spectra of TTF-COF (blue) and R-TTF$^{\bullet+}$-COF (red) measured from 200 nm to 1400 nm. Insert is the corresponding Tauc plots showing an optical bandgap.

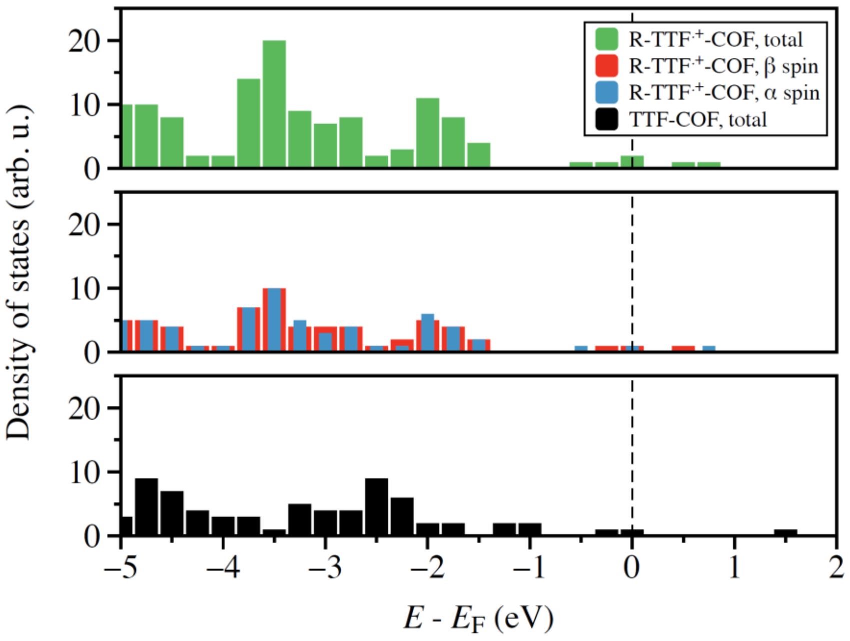

Figure S16 The density of states (DOS) for TTF-COF (bottom panel, black) and R-TTF$^{\cdot+}$-COF (top panel, green) systems. The middle panel shows the DOS for alpha (blue) and beta (red) spin polarizations. The DOS in each case is represented as a histogram of electronic states close to the HOMO energy (Fermi level), whose energy is set to zero in all cases. The bin size for the histograms is set to 0.25 eV. Note that only the LUMO state is shown above the Fermi level.

Table S1 Elemental analysis results of C, N, H, S elements in the TTF-COF, R-TTF$^{\cdot+}$-COF (200 °C_5 h), R-TTF$^{\cdot+}$-COF (300 °C_5 h), R-TTF$^{\cdot+}$-COF (360 °C_2 h), R-TTF$^{\cdot+}$-COF (360 °C_5 h).

SAMPLE	C %	N %	H %	S %
TTF-COF	70.08	7.11	3.79	16.12
R-TTF$^{\cdot+}$-COF (200 °C_5 h)	68.2	7.20	3.01	15.54
R-TTF$^{\cdot+}$-COF (300 °C_5 h)	59.58	5.14	2.47	29.84
R-TTF$^{\cdot+}$-COF (360 °C_2 h)	54.60	4.7	2.02	38.96
R-TTF$^{\cdot+}$-COF	47.59	4.38	1.46	42.32

(360 °C_5 h)

Table S2 Characteristic IR absorption (cm^{-1}) of tetrathiafulvalene groups in TTF-COF and R-TTF$^{\bullet+}$-COF.[17]

**Groups**	**TTF-COF (TTF)**	**R-TTF$^{\bullet+}$-COF (TTF$^{\bullet+}$)**
C=C stretching	1560,1506	1479
C-S stretching	721,836	732,839
C-C-S bending	783	819

Table S3 Experimentally measured Raman signals of sulfur species in DOL/DME solvent and theoretically calculated reported values in the literatures.

Sulfur species	Experimental values cm^{-1}	DFT calculations
S_8^{2-}	365[20], 475[21], 450[20], 400[25], 508[26]	459[22]
S_7^{2-}	390[20], 265[20]	
S_6^{2-}	365[20], 400[25], 399[26-27]	453[22]
S_5^{2-}	119[20]	
S_4^{2-}	200[20,25], 449[27], 450[20], 453[25], 501[26], 199[26-27]	369[22], 442[22]
S_4^{2-} + S_6^{2-}	395[21]	
S_3^{2-}	200[20], 450[20]	
S_2^{2-}	-	420[22], 440[24]
$S_3^{\bullet-}$	533[20], 532[22], 535[23], 536[25]	504[22]
$S_4^{\bullet-}$, $S_7^{\bullet-}$	500-518[20]	-

The different sulfur species might have the spectral overlap in the Raman analysis, especially for the dianionic polysulfides S_x^{2-} (X=4-8) that have the Raman signals in the range of 100-500 cm^{-1}, as summarized in **Table S3**. The Raman shifts for the different species have been confirmed by experimental results and computations.[20-27] As for the $S_3^{\bullet-}$, the fingerprint is located in the 530-535 cm^{-1}, which is very distinct to the other sulfur species. Though some papers report the $S_4^{\bullet-}$ and $S_7^{\bullet-}$ with

the possible Raman shift at ~510-518 cm^{-1}, they are generally short-lived intermediates and only form transiently under electrochemical or single-electron transfer conditions. Therefore, in our system, the stable sulfur species in R-TTF$^{\bullet+}$-COF can be assigned as $S_3^{\bullet-}$.

Table S4 Experimentally measured XPS signals of sulfur species in the literatures.

Sulfur species	BE S $2p_{2/3}$ (eV)
S_n^{2-} (n = 6-8)	163-164[28,29,31-33]
S_n^{2-} (n = 3-4)	162.0-162.9[30-33]
S_n^{2-} (n = 2)	161.3-162.0[29,31]
S^{2-}	160.5-161.2[28,30,31,33]
C-S	163-164[28,30]
Oxidized S	> 166[28-32]

The characteristic binding energies of sulfur species are summarized in **Table S4**. In general, longer sulfur chains exhibit higher binding energy (BE) values within the same system, providing a useful indicator for evaluating the sulfur conversion pathway in Li–S batteries. However, these BE values can be influenced by the local chemical environment, leading to slight shifts across different systems. The assignments can also be challenging when the chain lengths are similar. In our system, the binding energy at 162.7 eV of sulfur species in R-TTF$^{\bullet+}$-COF can be attributed to either trisulfide or tetrasulfur species. Combined with Raman analysis and the sulfur content determined by TGA and elemental analysis, the sulfur species can be assigned to $S_3^{\bullet-}$

Table S5 Parameters for four-probe conductivity measurement of TTF-COF.

Probe variable	Value1	Value2	Value3	Mean Value
Total length L1 (mm)	48.70	48.66	48.62	48.66
Probe length L (mm)	0.665	0.625	0.585	0.625
Probe diameter d (mm)	4.980	4.990	5.010	4.993

	Value1	Value2	Value3	Value4	Value5	Mean Value
Applied current I (μA)	0.19998	0.39999	0.59999	0.80001	1.00002	0.60000
Probe voltage U (V)	1.995	3.991	5.986	7.981	9.976	5.986
Calculated conductivity ρ (S/m)	3.199E-06	3.199E-06	3.199E-06	3.199E-06	3.199E-06	3.199E-06
DC resistivity (Ω m)	312601.353	312624.907	312596.191	312574.019	312563.842	312592.062

Table S6 Parameters for four-probe conductivity measurement of R-TTF$^{•+}$-COF.

Probe variable	Value1	Value2	Value3	Mean Value
Total length L1 (mm)	48.79	48.78	48.80	48.79
Probe length L (mm)	0.755	0.745	0.765	0.755
Probe diameter d (mm)	4.980	4.990	5.010	4.993

	Value1	Value2	Value3	Value4	Value5	Mean Value
Applied current I (μA)	0.19999	0.40002	0.60001	0.79999	1.00000	0.60000
Probe voltage U (V)	1.995	3.992	5.988	7.984	9.980	5.988
Calculated conductivity ρ (S/m)	3.865	3.863	3.863	3.863	3.863	3.864
DC resistivity (Ω m)	0.259	0.259	0.259	0.259	0.259	0.259

Table S7 Overview of electrically conductive COFs (σ > 0.0001 S m^{-1}).

COF	Electrical conductivity S/m	Method	Ref.
**R-TTF$^{\bullet+}$-COF**	3.9	*Four-probe, pellet*	*This work*
D-scu-COF-1, 3D-scu-COF-2	0.0032 – 0.0035	*Four-probe, pellet*	*J. Am. Chem. Soc.* **2022**,*144*, 17209.
I_2@TTF-Ph-COF I_2@TTF-Py-COF	0.001 – 0.0001	*Two-probe, pellet*	*Chem. Eur. J.*, **2014**, 20, 14608
TTF-DMTA	0.00018	*Two-probe, pellet*	*ACS Appl. Mater. Interfaces.* **2020**,12, 19054
I_2@TANG-COF	1	*Four-probe, pellet*	*J. Am. Chem. Soc.* **2020**,142, 2155
I_2@sp2c-COF	0.071	*Two-probe, pellet*	*Science.* **2017**, 357, 673.
PyVg-COF	0.4	*Two-probe, film*	*Chem. Sci.* **2019**, 10, 1023.
I_2@JUC-518	0.027	*Two-probe, pellet*	*J. Am. Chem. Soc.* **2019**, 141, 13324.
*p*FePc, *p*CoPc, *p*NiPc, *p*CuPc	*0.0018 - 0.15*	*Four-probe, pellet*	*J. Am. Chem. Soc.* **2023**,*145*, 24230.
TCNQ@SBFdiyne-COF	3.4	*Two-probe, film*	*Small.* **2021**, **17**, e2103152.

4. Kinetics study of COFs catalyzing polysulfide conversion.

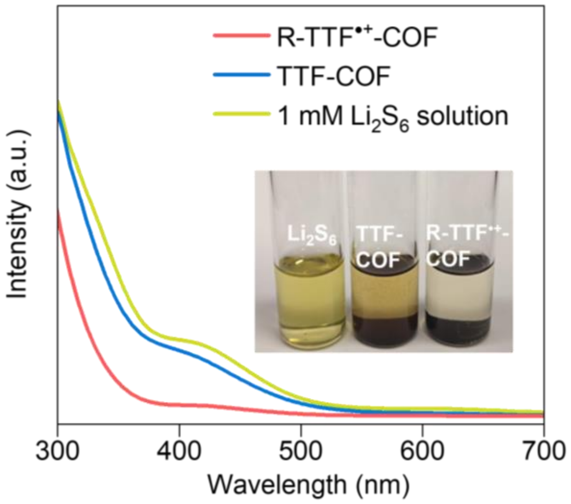

Figure S17 UV-vis spectra of the supernatant solution immersed with 5 mg COF powders and the corresponding photos of the static Li_2S_6 adsorption tests (insert picture).

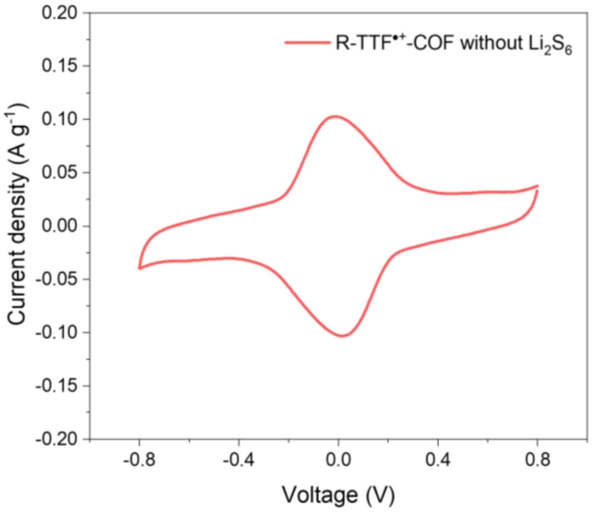

Figure S18 CV curves at a scan rate of 5.0 mV s^{-1} in the symmetric cells without Li_2S_6 using two identical electrodes with R-TTF$^{\bullet+}$-COF catalysts.

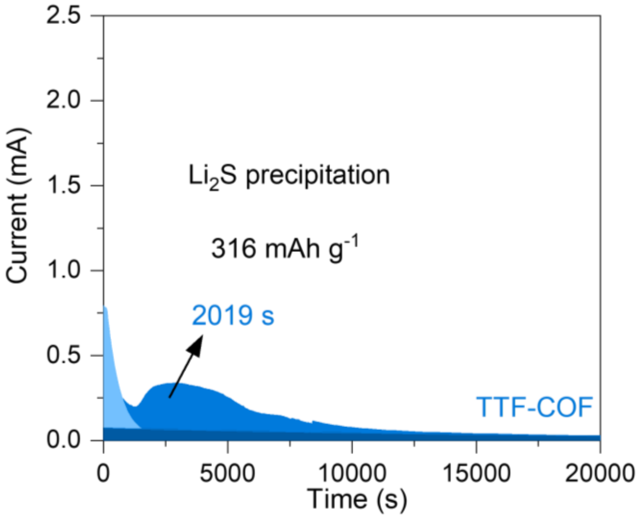

Figure S19 The potentiostatic Li_2S precipitation process at 2.05 V on TTF -COF electrode.

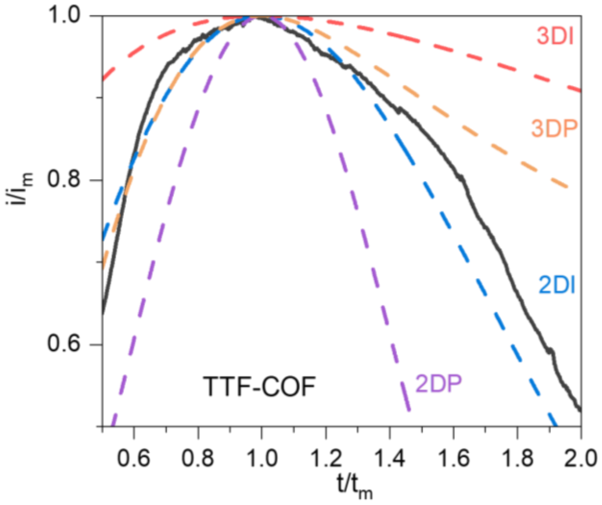

Figure S20 The derived dimensionless transient of TTF-COF fitted with theoretical 2D/3D models, respectively (t_m: time needed to reach the peak current; i_m: peak current).

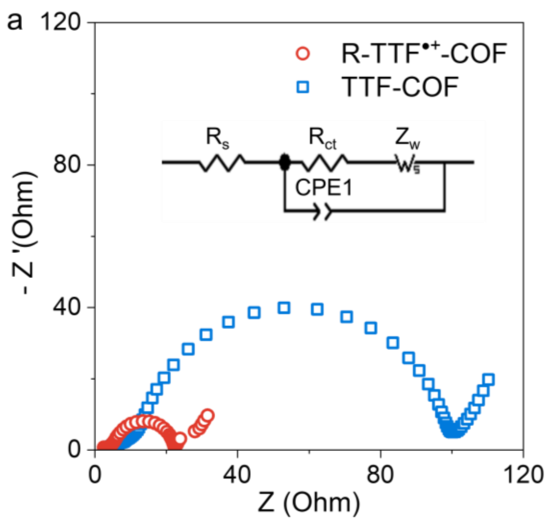

b

Sample	R_s (Ω)	R_{ct} (Ω)
TTF-COF|TTF-COF with Li_2S_6 symmetric cell	5.9	99.8
R-TTF$^{•+}$-COF|R-TTF$^{•+}$-COF with Li_2S_6 symmetric cell	4.2	21.6

Figure S21 (a) Nyquist plots of Li_2S_6 COF|COF symmetric cells with TTF-COF and R-TTF$^{•+}$-COF, respectively. The inset shows the equivalent circuit model used for fitting the impedance data. (b) Solution resistance (R_s) and charge-transfer resistance (R_{ct}) values obtained from the fitted spectra of Li_2S_6 COF|COF symmetric cells with TTF-COF and R-TTF$^{•+}$-COF, respectively.

Table S8 A. Beweick, M. Fleischmann, and H.R. Thirsk (BFT) 2D model with two forms (1) instantaneous (2DI) and (2) progressive (2DP) and Scharifker-Hills (SH) 3D model with two forms: (1) instantaneous (3DI) and (2) progressive (3DP).[34]

2D progressive (2DP)	2D instantaneous (2DI)
$\frac{I}{I_m}=\left(\frac{t}{t_m}\right)^2 \exp\left[-\frac{2}{3}\left(\frac{t}{t_m}\right)^3+\frac{2}{3}\right]$	$\frac{I}{I_m}=\frac{t}{t_m}\exp\left[-\frac{1}{2}\left(\frac{t}{t_m}\right)^2+\frac{1}{2}\right]$
3D progressive (3DP)	3D instantaneous (3DI)
$\frac{I}{I_m}=\left(\frac{1.2254t_m}{t}\right)^{0.5}\{1-\exp\left[-2.3367\left(\frac{t}{t_m}\right)^2\right]\}$	$\frac{I}{I_m}=\left(\frac{1.9542t_m}{t}\right)^{0.5}[1-\exp\left(\frac{-1.2564t_m}{t}\right)]$

5. Electrochemical measurements of Li-S batteries

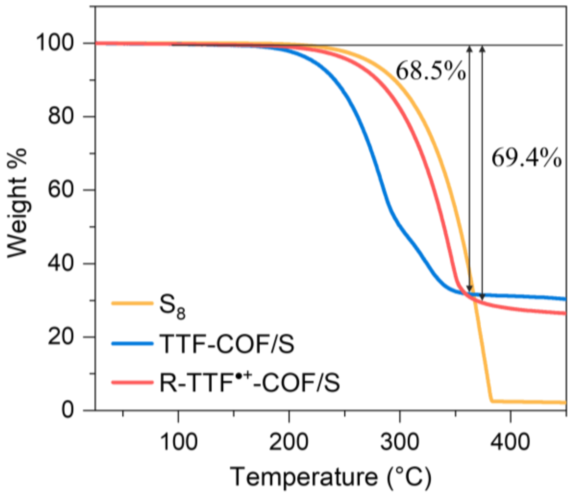

Figure S22 Comparisons of TGA curves for TTF-COF/S, R-TTF$^{\bullet+}$-COF/S, and pure S_8. The sulfur loading in the two hosts has been carefully tuned to be comparable. Noted that trisulfide species are also considered as a portion of the sulfur content in R-TTF$^{\bullet+}$-COF/S composites.

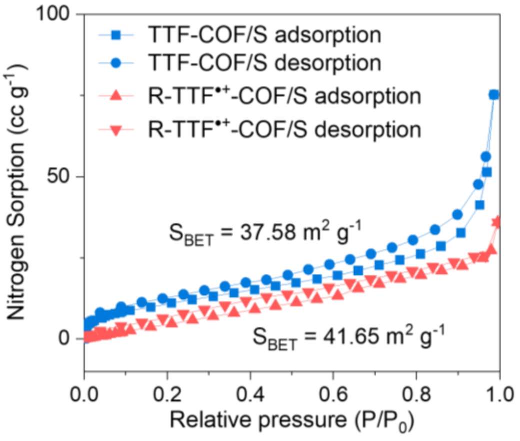

Figure S23 Adsorption/desorption isotherms of R-TTF$^{\bullet+}$-COF/S and TTF-COF/S composites in N_2 at 77 K. The decrease in the specific surface area of R-TTF$^{\bullet+}$-COF/S and TTF-COF/S composites implies the incorporation of sulfur into the COF pores.

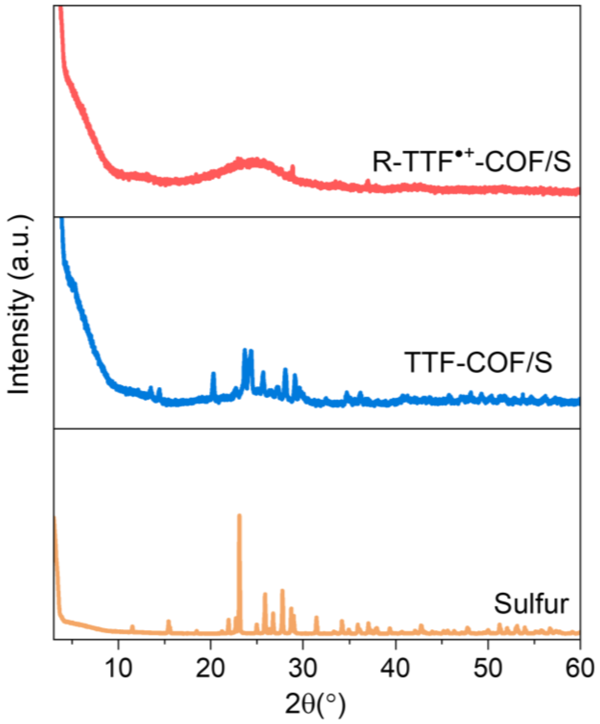

Figure S24 PXRD spectra of R-TTF$^{•+}$-COF/S, TTF-COF/S, and elemental sulfur. The PXRD pattern of TTF-COF/S reflects the feature of elemental sulfur, whereas R-TTF$^{•+}$-COF/S barely shows PXRD fingerprints of S_8, indicating the stronger interactions between R-TTF$^{•+}$-COF and S_8.

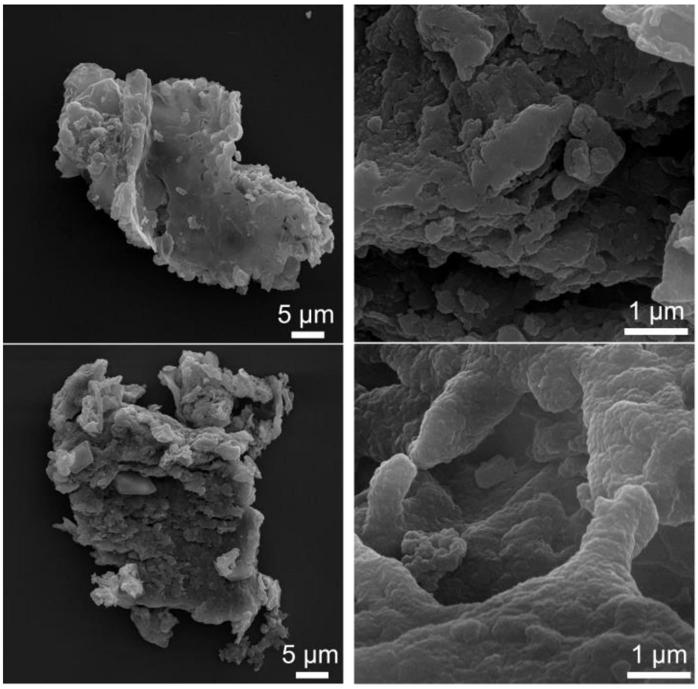

Figure S25 SEM images of different regions in the TTF-COF/S composite powder sample.

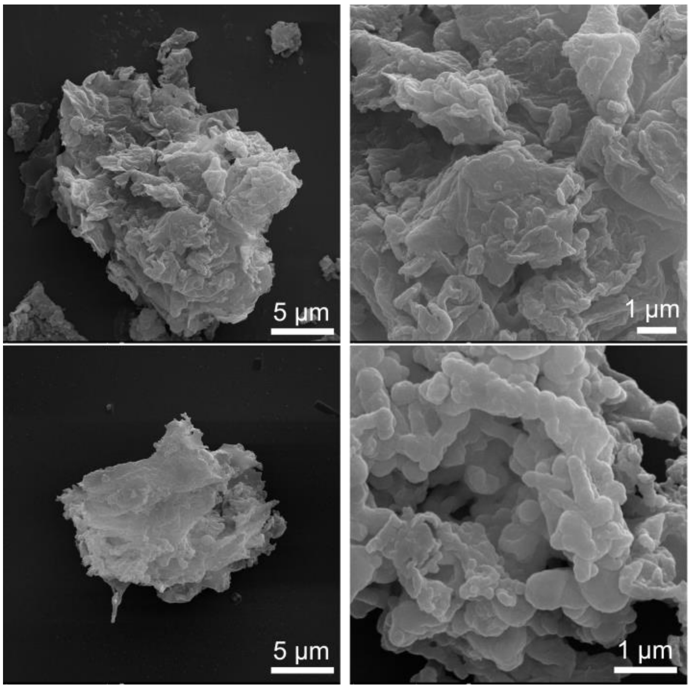

Figure S26 SEM images of different regions in R-TTF$^{\bullet+}$-COF/S composite powder sample.

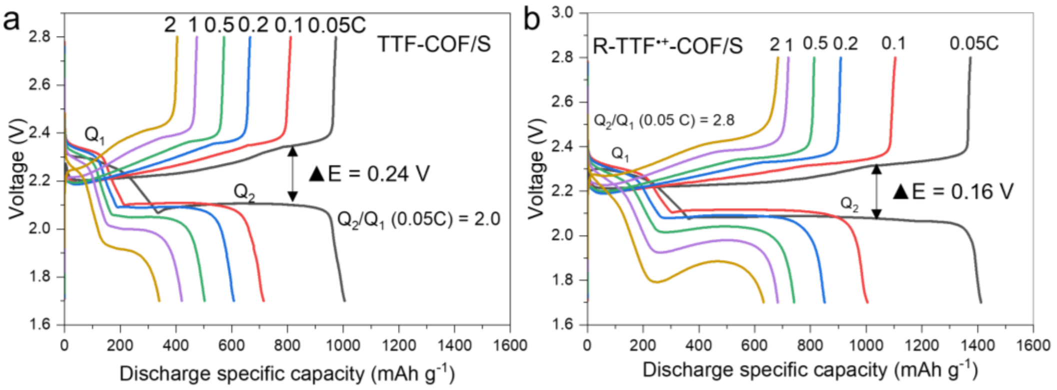

Figure S27 Galvanostatic (dis)charge of Li-S cells with R-TTF$^{\bullet+}$-COF/S and TTF-COF/S cathode at different current densities of 0.05 C, 0.1 C, 0.2 C, 0.5 C, 1 C and 2 C. Q1 and Q2 represent the capacity contribution of the first plateau and second plateau, respectively. Q2/Q1 stands for the sulfur conversion efficiency.

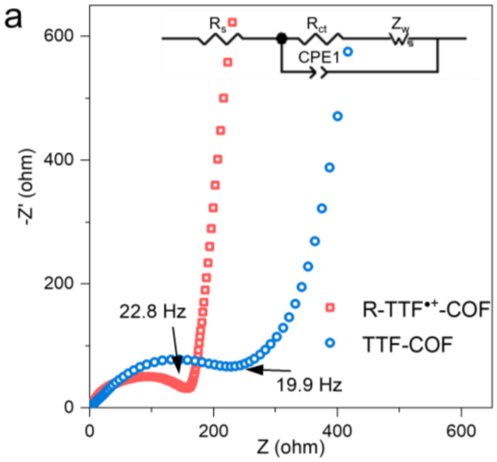

b

Sample	R_s (Ω)	R_{ct} (Ω)
TTF-COF/S coin cell	2.9	340.1
R-TTF$^{\bullet+}$-COF/S coin cell	3.1	123.2

Figure S28 (a) Nyquists plots of R-TTF$^{\bullet+}$-COF/S and TTF-COF/S coin cells after resting for 6 h. The inset shows the equivalent circuit model used for fitting the impedance data. (b) Solution resistance (R_s) and charge-transfer resistance (R_{ct}) values obtained from the fitted spectra of TTF-COF/S and R-TTF$^{\bullet+}$-COF/S coin cells.

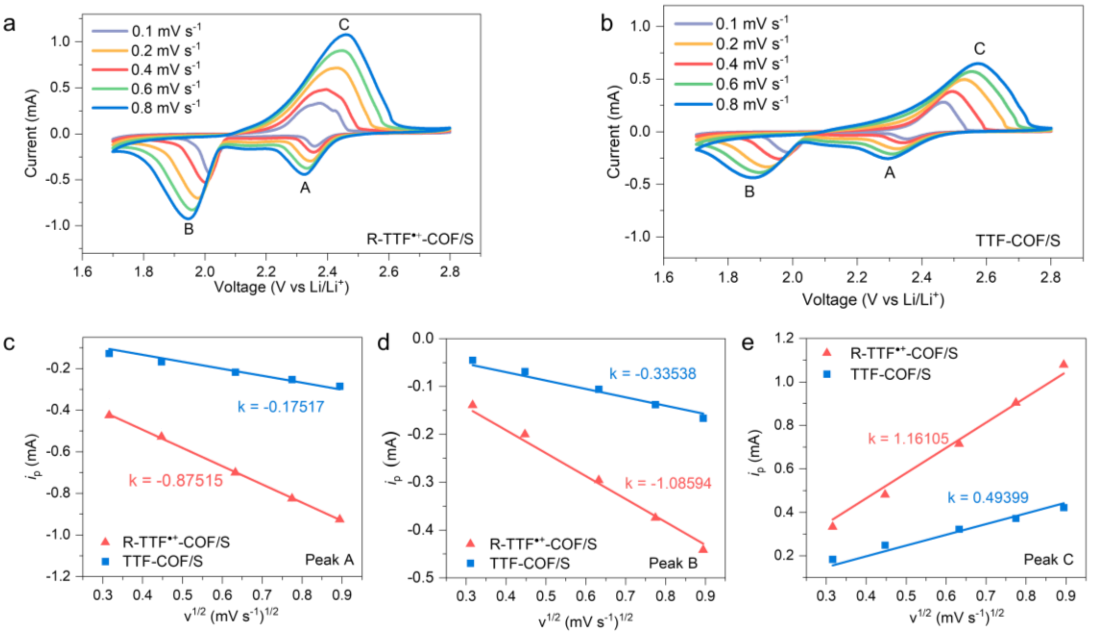

Figure S29 CV profiles of (a) R-TTF$^{\bullet+}$-COF/S and (b) TTF-COF/S assembled electrodes at different scan rates of 0.1, 0.2, 0.4, 0.6, and 0.8 mV s^{-1}. Linear fit of peak current vs. the square root of scan rate for peaks (c) A, (d) B, and (e) C for R-TTF$^{\bullet+}$-COF/S (red) and TTF-COF/S (blue), respectively.

The Randles–Sevcik equation was used to calculate the diffusion coefficients of various active materials[35]:

$$D^{0.5} = \frac{i_P}{269000n\,(an)^{0.5}Acv^{0.5}}$$

where i_p is the peak current (A), A is the electrode area (cm^2, here assumed to be 1.13 cm^2), c is the concentration of Li^+ (0.0014 mol mL^{-1}), v is the scan rate (V s^{-1}), D is the diffusion coefficient (cm^2 s^{-1}), n is the number of transferred electrons, and a is transfer coefficient (0.5 based on the assumption that the peak current is gained from the reversible process).

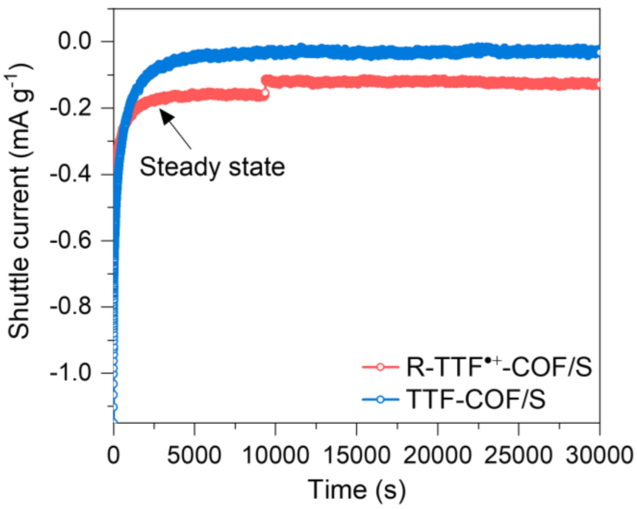

Figure S30 Shuttle current measurements of R-TTF$^{\bullet+}$-COF/S and TTF-COF/S derived coin cells when the cells are held at a constant potential of 2.35 V.

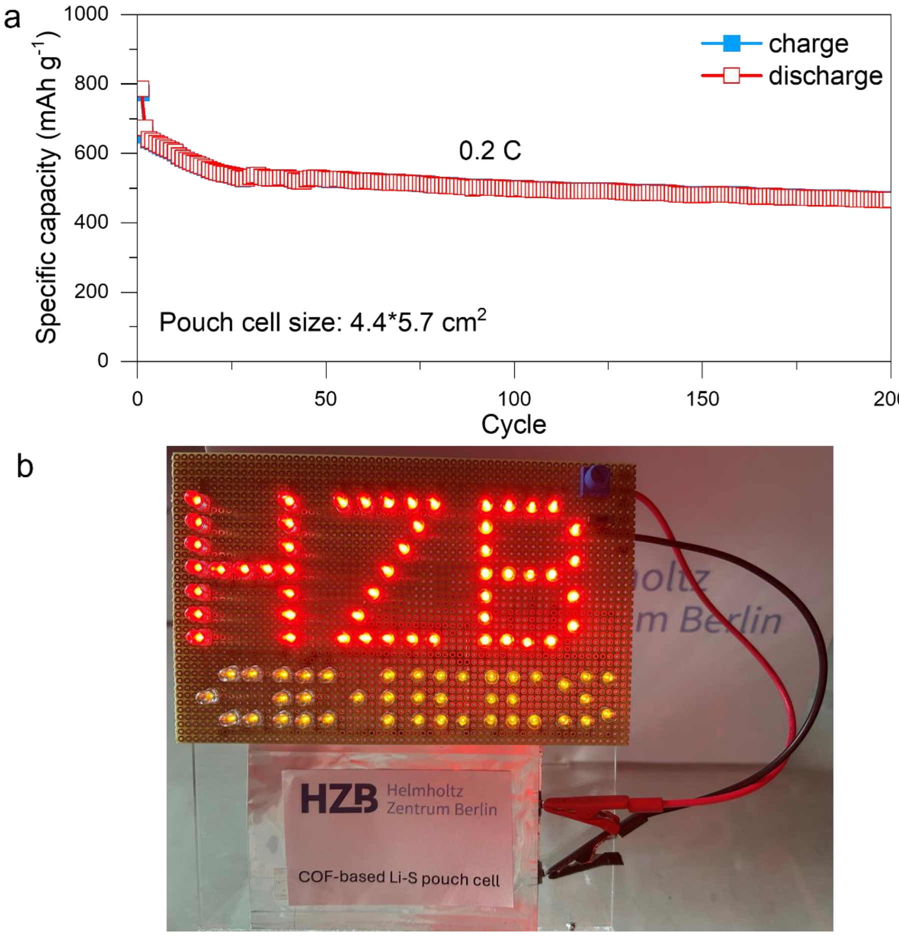

Figure S31 (a) The cycling performance of R-TTF$^{\bullet+}$-COF/S pouch cell over 200 cycles (E/S ratio: 11 μL mg_s^{-1}). (b) Demonstration test of the R-TTF$^{\bullet+}$-COF/S pouch cell illuminating the LED light featuring the HZB CE-IEES logo. The LED is made of 52 red LED (HZB) and 40 yellow LED (CE-IEES) series.

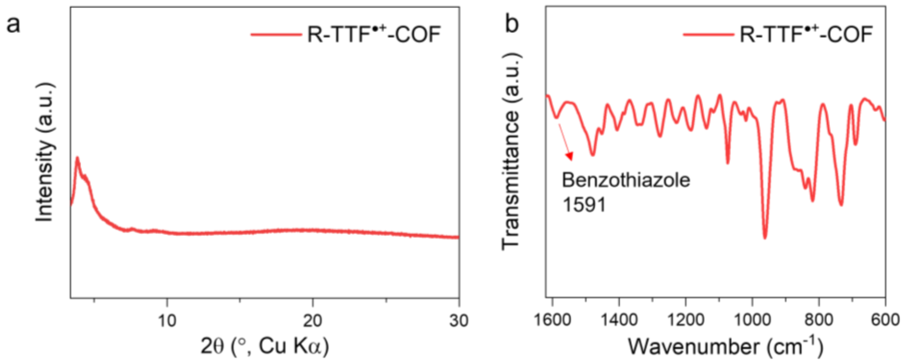

Figure S32 (a) PXRD and (b) FTIR of R-TTF$^{\bullet+}$-COF immersing in the electrolyte for 7 days.

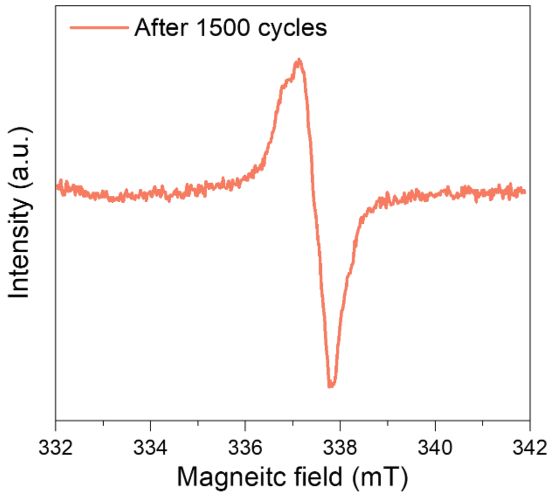

Figure S33 Solid-state EPR spectra of R-TTF$^{\bullet+}$-COF/S cathode materials after 1500 discharge-charge cycles.

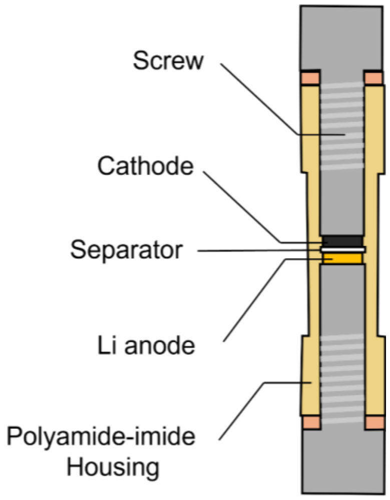

Figure S34 The cell configuration for *in situ* X-ray tomography measurement.

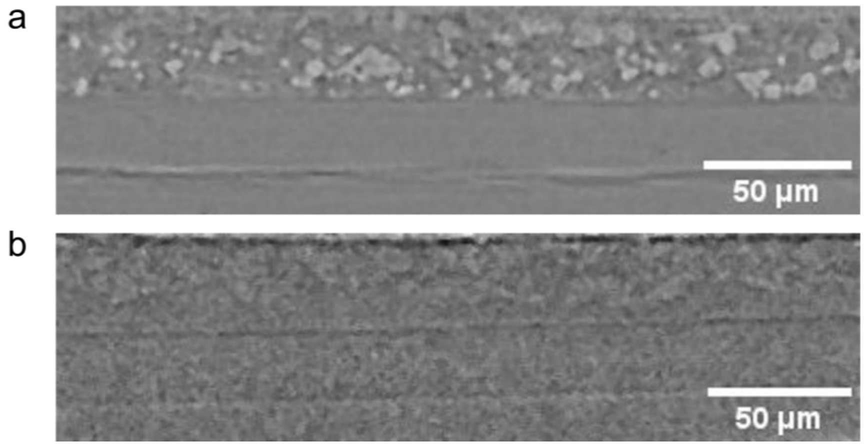

Figure S35 The cross-sectional X-ray tomographic 2D slices of the cathode/separator/Li region at (a) the initial state and (b) the discharged state.

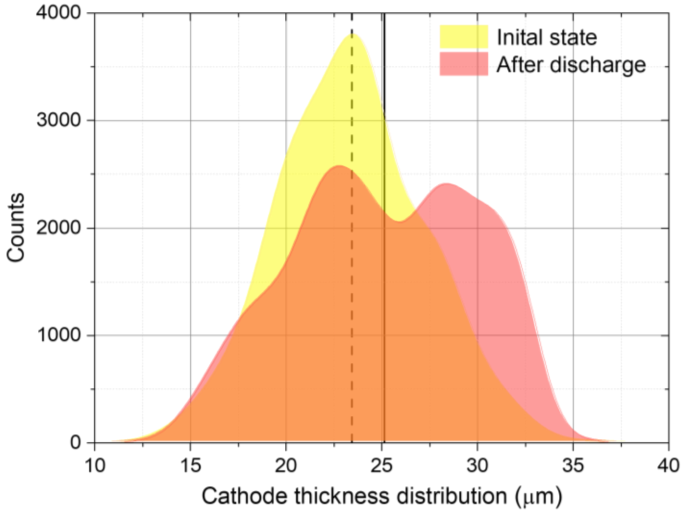

Figure S36 The thickness distribution of the cathode within the selected circular regions in Fig. 3h and 3j shows a mean thickness of 23.45 μm at the initial state and 25.14 μm at the discharged state.

Table S9 Performance comparisons in Li-S coin cells using COFs as sulfur hosts.

COFs as sulfur host	Sulfur content (sulfur loading)	Cycling performance (initial capacity and retention)	Capacity loss per cycle	Catalysis mechanism	Ref.
**R-TTF•+-COF**	~1.3 mg cm^{-2}, ~ 5.1 mg cm^{-2}	856 mAh g^{-1} at 0.5 C, 60.7% retention, 1500 cycles	0.027%	Dynamic covalent binding	This work
**EB-COF-PS**	~1.5 mg cm^{-2}	913 mAh g^{-1} at 0.5 C, 60.8 % retention, 300 cycles	0.13%	Electrostatic interactions	35
**3D-scu-COF-2**	~1.1 mg cm^{-2}	1021 mAh g^{-1} at 0.5 C, 90% retention, 100 cycles	0.10%	Enhanced electrical conductivity	36
**COF-ETTA-ETTCA**	~1.3 mg cm^{-2}	1005 mAh g^{-1} at 0.5 C, 58% retention, 528 cycles	0.08%	π-conjugated framework	37
**COF-Tr-BA**	~1.5 mg cm^{-2}	1349 mAh g^{-1} at 0.5 C, 48% retention, 200 cycles	0.27%	Absorption by boric acid	38
**PI-COF**	< 1.0 mg cm^{-2}	900 mAh g^{-1} at 1C, 85% retention, 500 cycles	0.03%	Introducing redox-active moieties and increasing molecular polarity	39
**THZ-DMTD-COF**	~1.0 mg cm^{-2}	642 mAh g^{-1} at 1 C, 78.9% retention, 200 cycles	0.105%	Introducing redox-active moieties	40
**IP-COF-1**	~1.0 mg cm^{-2}, ~ 9 mg cm^{-1}	837 mAh g^{-1} at 2 C, 75.2% retention, 600 cycles	0.042%	Covalently immobilized LiPS	41
**NiS_4-TAPT**	~2.0 mg cm^{-2}	714 mAh g^{-1} at 1 C, 70% retention, 400 cycles	0.075%	Metal-center assisted adsorption	42
**COF-TPT**	~1.0 mg cm^{-2}	1330 mAh g^{-1} at 0.5 C, 55% retention, 1000 cycles	0.045%	Introducing polar hydroxyl groups	43

6. Analysis of discharged and charged samples to study the catalytic mechanism

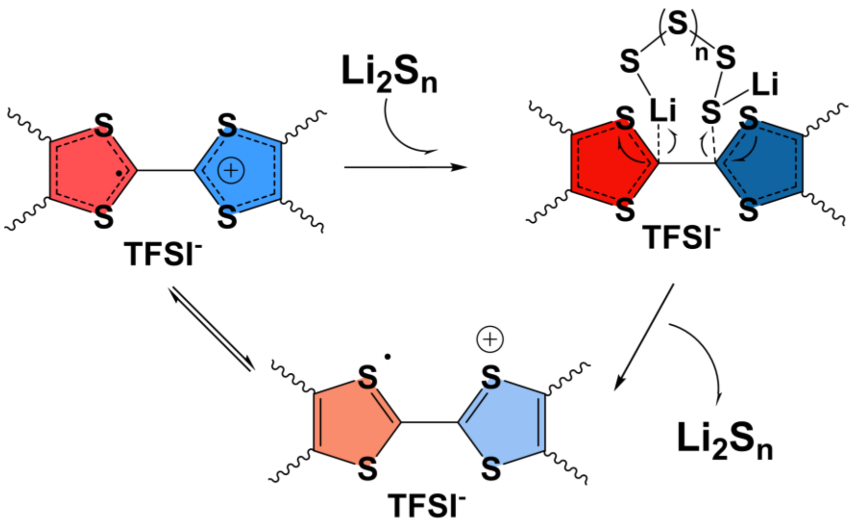

Scheme S3 The dynamic structural evolution of LiPSs on $TTF^{•+}$ during SRR.

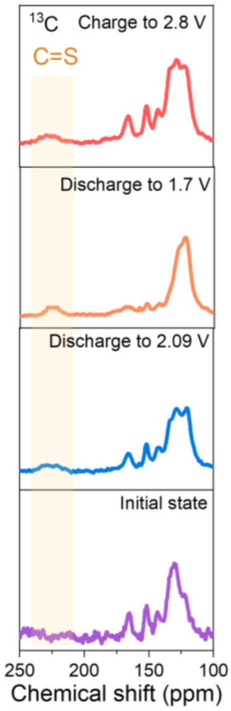

Figure S37 Solid-state CP/MAS ^{13}C NMR (250-100 ppm) of R-$TTF^{•+}$-COF/S cathodes at the first cycle.

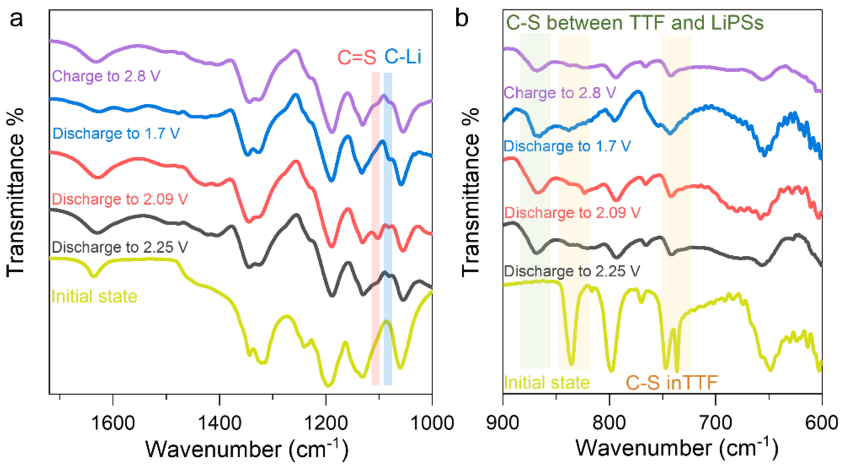

Figure S38 (a) *Ex situ* FTIR spectra of the R-TTF$^{•+}$-COF cathode at different states of (dis)charge from 1000 to 1700 cm^{-1} and (b) from 560 to 900 cm^{-1}. The signal intensity of the FTIR spectra was weakened compared to the initial state, probably due to the formation of a solid electrolyte interface and electrolyte residue on the electrode surface. When discharging to 2.25 V, 2.09 V, 1.7 V, and recharging to 2.8 V, the characteristic bands of the TTF$^{•+}$ unit in discharged cathodes shift relative to the initial state, which reveals a different environment surrounding TTF$^{•+}$ under electrochemical conditions.

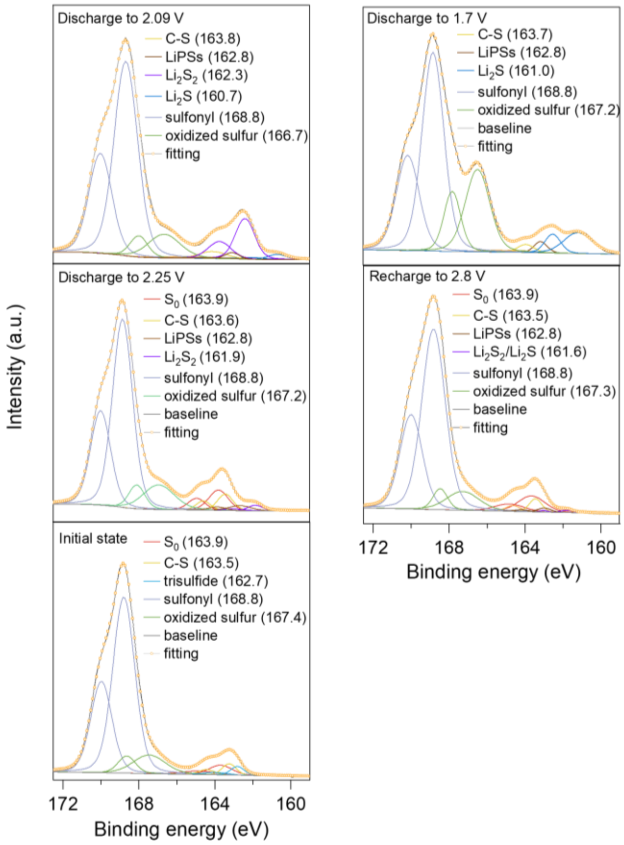

Figure S39 *Ex-situ* high-resolution S 2p XPS spectra of R-TTF$^{\bullet+}$-COF cathode at the initial and different (dis)charge states during the first cycle. The strong peak at 168.8 eV belongs to the sulfonyl groups in LiTFSI and the other peaks beyond 166 eV come from the oxidized sulfur. When discharging to 2.25 V, 2.09 V, 1.7 V, all the C-S peaks shift to higher binding energy, which suggests a change in the TTF surroundings. When discharged to 2.25 V, a portion of sulfur is converted to LiPSs. Meanwhile, there is one new component at 161.9 eV that can be assigned to Li_2S_2[44], because of the reaction between trisulfides in R-TTF$^{\bullet+}$-COF and Li^+. When discharged to 2.09 V, sulfur is mostly reduced to a product at 162.3 eV, which can be assigned to the Li_2S_3 or Li_2S_2 interacting with TTF. When discharged to 1.7 V, the peak for Li_2S is located at higher energy (161.0 eV) compared to those reported in the literatures (160.5-161.0 eV)[45], which is ascribed to the strong interaction between Li_2S and R-TTF$^{\bullet+}$-COF. When recharging to 2.8 V, most sulfur is oxidized to S_8; however, an emerging weak signal at 161.6 eV may hint that a few sulfides are connected to C in TTF$^{\bullet+}$ groups, supporting the ^{13}C ssNMR results.

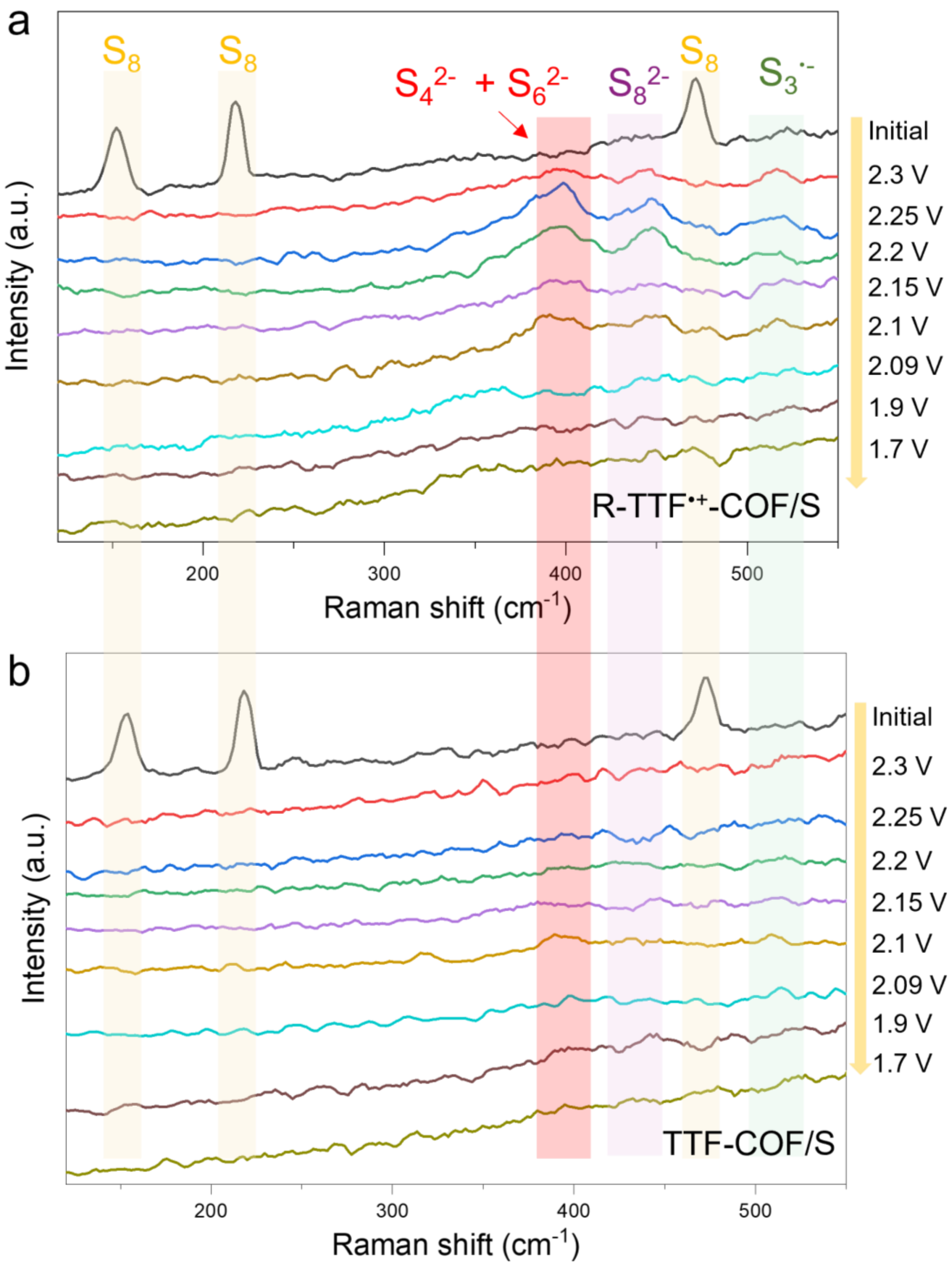

Figure S40 *Operando* Raman measurement during the first discharge cycle for (a) R-TTF$^{\bullet+}$-COF/S and (b) TTF-COF/S cathodes.

Table S10 Characteristic IR adsorption (cm^{-1}) corresponding to **Figure S38**.

Species	Wavenumber (cm^{-1})	Vibrations	Ref.
LiTFSI	1346	Asymmetric SO_2 stretching mode	46
	1326	C–SO_2–N bending mode	
	1193	Asymmetric stretching mode of –CF_3	
	1132	C–SO_2–N bending	
	1061	Asymmetric S–N–S stretching	
	796	-CF_3 symmetric bending mode	
	746	S–N stretching mode	
PVDF	1405	–CH_2	47
$LiNO_3$	1340	–NO_3^-	48
Li- $TTF^{\bullet+}$	1082 (2.25V, 2.08V, 1.7V, 2.8V)	C–Li	49
$TTF^{\bullet+}$ group	1102 (2.25V, 2.08V, 1.7V, 2.8V)	C=S	50
	838 (initial state), 840 (2.08V, 1.7V, 2.8V)	C–S stretching in $TTF^{\bullet+}$	
	736 (initial state), 732 (2.08V, 1.7V, 2.8V)	C–S stretching in $TTF^{\bullet+}$	
	868 (2.25V, 2.08V, 1.7V, 2.8V)	C-S stretching when interacting with sulfur-chain	51

7. Computational studies to understand the catalytic mechanism

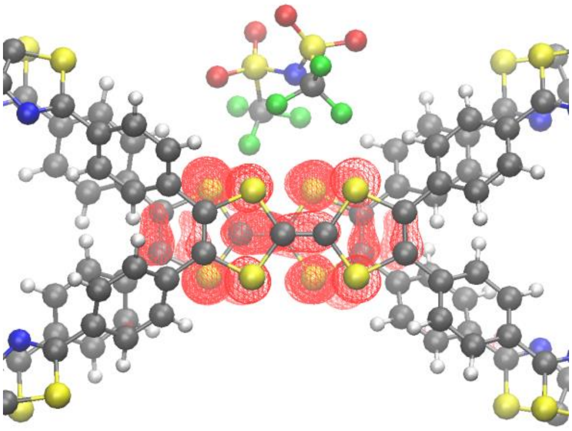

Figure S41 Spin density plots of R-TTF$^{\bullet+}$-COF stabilized by TFSI^{-} upon discharge.

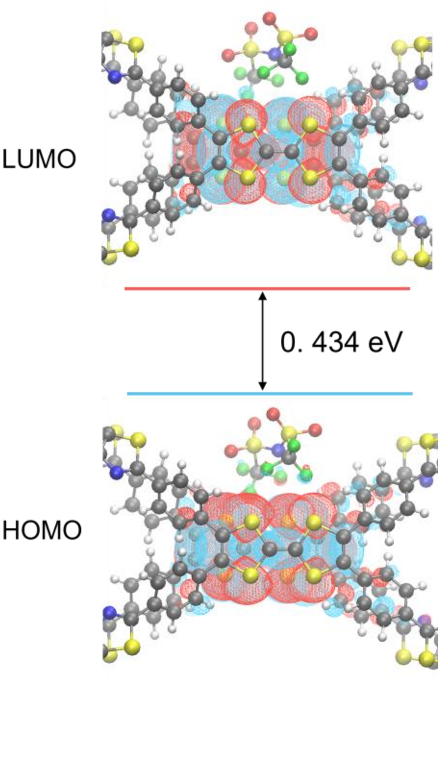

Figure S42 Distribution of HOMO/LUMO of the bilayer molecular unit in R-TTF$^{\bullet+}$-COF stabilized by TFSI^{-} and the bandgap.

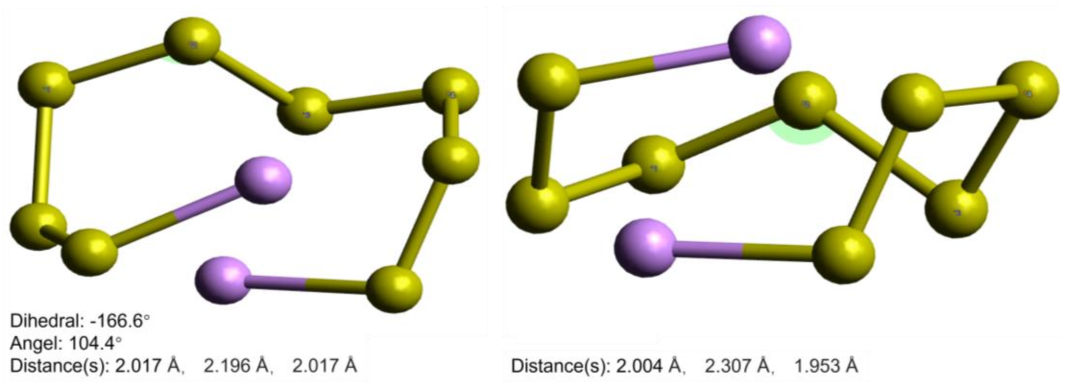

Figure S43 Left panel: Optimized structure of a Li_2S_8 molecule in the gas phase. Right panel: Optimized structure of a Li_2S_8 molecule in the presence of R-TTF$^{\bullet+}$-COF stabilized by $TFSI^-$. Both structures are optimized at the PBE level of theory.

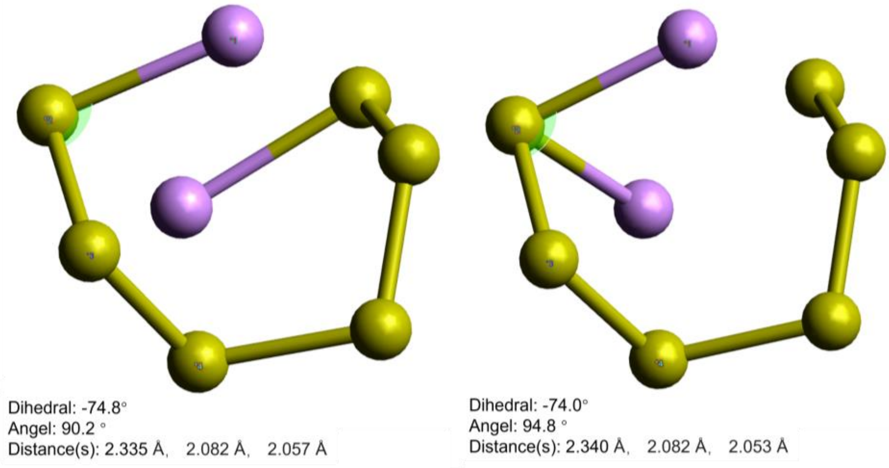

Figure S44 Left panel: Optimized structure of a Li_2S_6 molecule in the gas phase. Right panel: Optimized structure of a Li_2S_6 molecule in the presence of R-TTF$^{\bullet+}$-COF stabilized by $TFSI^-$. Both structures are optimized at the PBE level of theory.

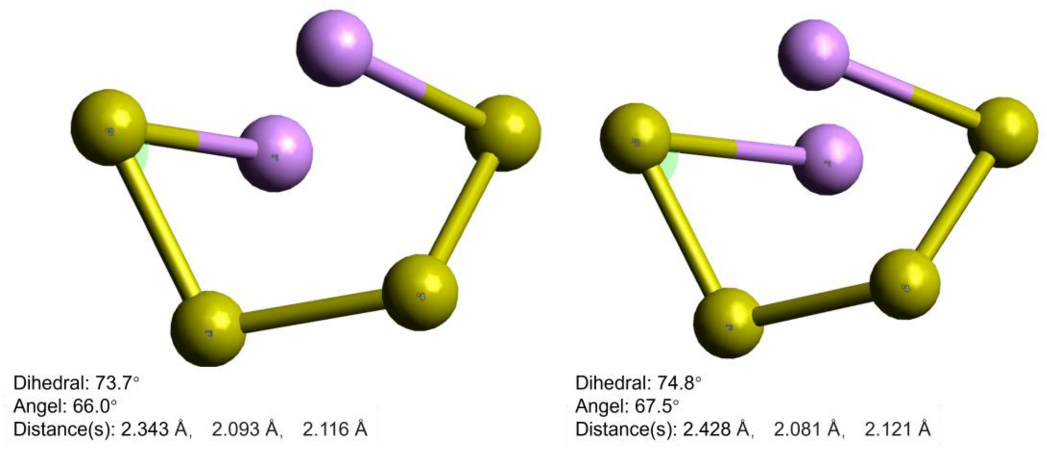

Figure S45 Left panel: Optimized structure of a Li_2S_4 molecule in the gas phase. Right panel: Optimized structure of a Li_2S_4 molecule in the presence of R-TTF$^{\bullet+}$-COF stabilized. Both structures are optimized at the PBE level of theory.

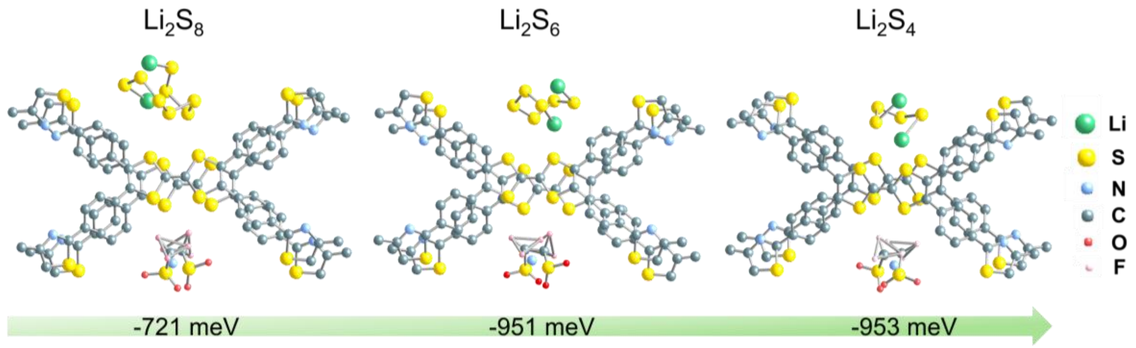

Figure S46 The interaction energies between R-TTF$^{\bullet+}$-COF and lithium polysulfides.

Table S11 Atom coordinates in the unit cell of TTF-COF.

Atom	Label	x	y	z
C	C0	0.52582	0.81359	0.96491
C	C1	0.52519	0.7568	0.97339
C	C10	0.8764	0.67454	0.98739
C	C100	0.98627	0.39183	0.5426
C	C101	0.04066	0.3774	0.45881
C	C102	0.04486	0.42735	0.38158
C	C103	0.09124	0.31214	0.45766
C	C104	0.10111	0.67266	0.52334
C	C107	0.20164	0.65099	0.57464
C	C108	0.19078	0.23384	0.3944
C	C109	0.25155	0.22151	0.42859
C	C11	0.87569	0.54265	0.00461
C	C110	0.18077	0.18237	0.35318
C	C111	0.19078	0.71357	0.60363
C	C112	0.26258	0.60311	0.54424
C	C113	0.54146	0.84078	0.48163
C	C114	0.54055	0.88929	0.58245
C	C115	0.48847	0.94906	0.58812
C	C116	0.43593	0.96291	0.49119
C	C117	0.43731	0.91488	0.38734
C	C118	0.48907	0.8553	0.38244
C	C119	0.54247	0.7086	0.45846
C	C12	0.87596	0.72178	0.88582
C	C120	0.49018	0.74431	0.55631
C	C121	0.43991	0.73384	0.54898
C	C122	0.44025	0.68627	0.4452
C	C123	0.49235	0.65033	0.34794
C	C124	0.54279	0.66106	0.35495
C	C125	0.38926	0.67218	0.44328

C	C126	0.38273	0.02639	0.49659
C	C129	0.28967	0.10781	0.39697
C	C13	0.92931	0.63852	0.08576
C	C130	0.29996	0.15961	0.43307
C	C131	0.22967	0.12062	0.35103
C	C132	0.29847	0.68124	0.55176
C	C133	0.23848	0.72809	0.59484
C	C134	0.30998	0.61802	0.52997
C	C14	0.98021	0.64796	0.07936
C	C15	0.98001	0.69462	0.97573
C	C16	0.92709	0.73116	0.87896
C	C17	0.92877	0.52675	0.91125
C	C18	0.87456	0.49454	0.09936
C	C19	0.92484	0.43344	0.10228
C	C20	0.97794	0.4182	0.01107
C	C21	0.97898	0.46611	0.91519
C	C22	0.02956	0.35364	0.01214
C	C23	0.0317	0.70751	0.97059
C	C26	0.12286	0.70134	0.07316
C	C27	0.12446	0.27472	0.91449
C	C28	0.18576	0.26299	0.90062
C	C29	0.11304	0.2226	0.90534
C	C30	0.11145	0.76316	0.03311
C	C31	0.18282	0.65638	0.12435
C	C32	0.47375	0.87867	0.95418
C	C33	0.4776	0.92892	0.03491
C	C34	0.42823	0.99051	0.03114
C	C35	0.37345	0.00456	0.94516
C	C36	0.3704	0.95495	0.85829
C	C37	0.41945	0.89345	0.86283
C	C38	0.47238	0.74519	0.9877
C	C39	0.41881	0.78666	0.0755
C	C4	0.64285	0.72572	0.97571

C	C40	0.36944	0.77492	0.08676
C	C41	0.37132	0.72101	0.01056
C	C42	0.4252	0.67854	0.92752
C	C43	0.47486	0.69017	0.91657
C	C44	0.31915	0.70883	0.01294
C	C45	0.32175	0.06903	0.95215
C	C48	0.22315	0.14902	0.88706
C	C49	0.23427	0.20135	0.89008
C	C5	0.70561	0.69418	0.98661
C	C50	0.16153	0.161	0.88859
C	C51	0.21982	0.73173	0.07614
C	C52	0.15893	0.77833	0.03756
C	C53	0.23071	0.67084	0.12295
C	C8	0.82281	0.66379	0.99008
C	C81	0.59545	0.77691	0.47997
C	C82	0.59576	0.71983	0.46669
C	C85	0.71249	0.6903	0.48312
C	C86	0.77527	0.6585	0.49207
C	C89	0.89324	0.62647	0.50494
C	C9	0.8225	0.60704	6.70E-04
C	C90	0.89202	0.56991	0.49477
C	C91	0.94634	0.63793	0.51851
C	C92	0.94327	0.50452	0.48128
C	C93	0.94288	0.69271	0.44677
C	C94	0.00135	0.5966	0.6039
C	C95	0.05143	0.60763	0.6091
C	C96	0.04869	0.66093	0.53038
C	C97	0.99302	0.70383	0.45262
C	C98	0.99715	0.48941	0.39211
C	C99	0.93846	0.45412	0.55381
H	H135	0.90136	0.72538	0.37971
H	H136	0.0047	0.55523	0.66561

H	H137	0.09374	0.57523	0.67378
H	H138	0.99008	0.74502	0.39029
H	H139	0.00121	0.52692	0.33002
H	H140	0.89772	0.46431	0.62417
H	H141	0.98234	0.35398	0.60382
H	H142	0.08629	0.41605	0.31313
H	H143	0.08276	0.27675	0.51988
H	H144	0.0923	0.71642	0.45969
H	H145	0.25926	0.26169	0.45299
H	H146	0.1347	0.19182	0.31619
H	H147	0.14491	0.75006	0.63891
H	H148	0.27128	0.55445	0.52988
H	H149	0.58027	0.87905	0.66069
H	H150	0.48801	0.9854	0.67009
H	H151	0.39711	0.92598	0.31015
H	H152	0.49015	0.81894	0.30014
H	H153	0.49027	0.77978	0.63997
H	H154	0.39986	0.76106	0.62542
H	H155	0.49309	0.61392	0.26581
H	H156	0.5823	0.63367	0.27678
H	H157	0.39166	0.637	0.35163
H	H158	0.38396	0.05809	0.59366
H	H159	0.34663	0.15077	0.45974
H	H160	0.22261	0.08085	0.31529
H	H161	0.23065	0.77589	0.62061
H	H162	0.35679	0.58058	0.50652
H	H163	0.19058	0.61009	0.16441
H	H54	0.83587	0.7497	0.80805
H	H55	0.92944	0.60334	0.16835
H	H56	0.02075	0.62031	0.15601
H	H57	0.92632	0.76699	0.79719
H	H58	0.92938	0.56269	0.83314
H	H59	0.83456	0.5059	0.17434

H	H60	0.92346	0.39721	0.1782
H	H61	0.01964	0.4539	0.84225
H	H62	0.0252	0.31941	0.09189
H	H63	0.02963	0.73948	0.87206
H	H64	0.19391	0.30353	0.89977
H	H65	0.06565	0.23131	0.90679
H	H66	0.06463	0.79958	0.00325
H	H67	0.51871	0.91889	0.10669
H	H68	0.43124	0.02803	0.10005
H	H69	0.32869	0.96599	0.78802
H	H70	0.41604	0.85604	0.79502
H	H71	0.4162	0.82845	0.13553
H	H72	0.32841	0.80709	0.15458
H	H73	0.42725	0.63697	0.86682
H	H74	0.51524	0.65751	0.84727
H	H75	0.32651	0.66421	0.95454
H	H76	0.32874	0.10238	0.03187
H	H77	0.28131	0.19303	0.87884
H	H78	0.15313	0.12068	0.87882
H	H79	0.15033	0.82602	0.00917
H	H80	0.27662	0.63604	0.1649
N	N105	0.15539	0.63369	0.58112
N	N106	0.14418	0.29705	0.39496
N	N127	0.33664	0.04465	0.39485
N	N128	0.3439	0.69953	0.54412
N	N24	0.0772	0.68315	0.07465
N	N25	0.078	0.33775	0.92481
N	N46	0.26984	0.08587	0.87634
N	N47	0.26624	0.74884	0.07349
S	S2	0.59908	0.80891	0.96294
S	S3	0.59787	0.6873	0.97004
S	S6	0.7503	0.73296	0.98356
S	S7	0.74954	0.611	0.00231

S	S83	0.66806	0.77368	0.48991
S	S84	0.66841	0.65101	0.46535
S	S87	0.82073	0.69626	0.50333
S	S88	0.8185	0.57515	0.492

Table S12 Atom coordinates in the unit cell of R-TTF$^{\bullet+}$-COF.

Atom	Label	x	y	z
C	C0	0.56907	0.84883	0.93132
C	C1	0.57008	0.79168	0.93793
C	C10	0.92554	0.71065	0.92673
C	C100	0.23638	0.27547	0.44543
C	C101	0.2969	0.2475	0.51101
C	C102	0.21507	0.23757	0.36002
C	C103	0.21833	0.75176	0.50003
C	C104	0.3011	0.65781	0.36285
C	C105	0.57545	0.88189	0.44783
C	C106	0.57295	0.93102	0.54845
C	C107	0.52031	0.99121	0.54962
C	C108	0.4678	0.00492	0.45139
C	C109	0.47049	0.95597	0.34933
C	C11	0.92663	0.57921	0.90152
C	C110	0.52341	0.89633	0.34649
C	C111	0.5767	0.74955	0.43312
C	C112	0.5239	0.78721	0.52892
C	C113	0.47184	0.77916	0.52278
C	C114	0.47084	0.73145	0.42296

C	C115	0.52409	0.6926	0.33029
C	C116	0.57595	0.70152	0.33435
C	C117	0.41531	0.72315	0.42188
C	C118	0.41176	0.06847	0.45018
C	C12	0.93165	0.75584	0.83269
C	C121	0.31465	0.14474	0.39544
C	C122	0.33511	0.18239	0.48561
C	C123	0.25365	0.17256	0.3326
C	C124	0.31808	0.74443	0.47031
C	C125	0.25674	0.7785	0.52677
C	C126	0.33914	0.68507	0.38711
C	C13	0.97393	0.67353	0.0375
C	C14	0.02783	0.67848	0.04803
C	C15	0.03425	0.72266	0.95105
C	C16	0.985	0.76209	0.84597
C	C17	0.9819	0.56677	0.82245
C	C18	0.92554	0.52816	0.9806
C	C19	0.9777	0.4674	0.98053
C	C20	0.03315	0.45599	0.90581
C	C21	0.03449	0.50686	0.82707
C	C22	0.08898	0.39175	0.90216
C	C23	0.09157	0.72855	0.96409
C	C26	0.18941	0.70743	0.93361
C	C27	0.18719	0.3146	0.93194
C	C28	0.24921	0.28524	0.98634
C	C29	0.16327	0.27829	0.8502
C	C30	0.16812	0.76368	0.03126
C	C31	0.25041	0.67611	0.87493
C	C32	0.5153	0.91441	0.92254
C	C33	0.51903	0.96523	0.99871
C	C34	0.46926	0.02715	0.99396
C	C35	0.41337	0.04133	0.9126
C	C36	0.41	0.99112	0.83121

C	C37	0.45981	0.92941	0.83571
C	C38	0.51762	0.77821	0.95236
C	C39	0.46306	0.81794	0.04053
C	C4	0.68906	0.76173	0.91653
C	C40	0.41394	0.80518	0.04809
C	C41	0.41709	0.75152	0.96806
C	C42	0.47228	0.7103	0.88633
C	C43	0.52143	0.72322	0.87882
C	C44	0.36324	0.74068	0.96825
C	C45	0.35935	0.10605	0.91251
C	C48	0.2628	0.1846	0.86627
C	C49	0.28598	0.22041	0.95404
C	C5	0.75203	0.73105	0.89975
C	C50	0.20047	0.21386	0.81379
C	C51	0.26756	0.75769	0.01676
C	C52	0.20649	0.78899	0.07544
C	C53	0.28849	0.70199	0.91668
C	C73	0.63075	0.81712	0.44754
C	C74	0.63122	0.7602	0.43826
C	C77	0.74959	0.72982	0.4439
C	C78	0.8134	0.69776	0.43502
C	C8	0.87067	0.70083	0.909
C	C81	0.93293	0.66569	0.43823
C	C82	0.93207	0.60965	0.41546
C	C83	0.98643	0.67779	0.4566
C	C84	0.98451	0.54358	0.39745
C	C85	0.97991	0.73693	0.41078
C	C86	0.04492	0.63247	0.52014
C	C87	0.09506	0.64388	0.52622
C	C88	0.08854	0.70229	0.47472
C	C89	0.0295	0.74933	0.42232
C	C9	0.87132	0.64406	0.90008
C	C90	0.03887	0.52869	0.30961

C	C91	0.98046	0.4928	0.46813
C	C92	0.02937	0.43039	0.45696
C	C93	0.08518	0.417	0.38182
C	C94	0.08883	0.46701	0.30502
C	C95	0.1402	0.35216	0.38739
C	C96	0.14341	0.71196	0.47193
C	C99	0.24014	0.69133	0.42189
H	H127	0.93598	0.7735	0.35962
H	H128	0.05197	0.58692	0.5628
H	H129	0.13988	0.60758	0.57333
H	H130	0.02277	0.7952	0.38252
H	H131	0.04299	0.56583	0.24624
H	H132	0.93944	0.50193	0.53736
H	H133	0.02469	0.39239	0.51377
H	H134	0.13069	0.45685	0.23863
H	H135	0.31235	0.27655	0.57844
H	H136	0.31694	0.61238	0.30073
H	H137	0.61198	0.92175	0.6296
H	H138	0.52	0.02739	0.63175
H	H139	0.43082	0.96596	0.26988
H	H140	0.52526	0.85981	0.26353
H	H141	0.52447	0.8229	0.61065
H	H142	0.43158	0.80879	0.59805
H	H143	0.52517	0.6554	0.25073
H	H144	0.61602	0.67092	0.25914
H	H145	0.23828	0.14362	0.26424
H	H146	0.24099	0.82378	0.58972
H	H147	0.26602	0.63403	0.79755
H	H54	0.8949	0.78568	0.74636
H	H55	0.96882	0.64002	0.11493
H	H56	0.06492	0.64869	0.1338
H	H57	0.98887	0.79705	0.77093
H	H58	0.98335	0.60486	0.75636

H	H59	0.884	0.53545	0.04472
H	H60	0.97505	0.42917	0.04329
H	H61	0.07676	0.49842	0.76535
H	H62	0.26663	0.31278	0.05295
H	H63	0.56076	0.95611	0.06762
H	H64	0.47398	0.06436	0.05887
H	H65	0.36762	0.00141	0.76395
H	H66	0.45528	0.89214	0.7711
H	H67	0.45847	0.86008	0.10297
H	H68	0.37217	0.83685	0.11578
H	H69	0.47697	0.66806	0.82343
H	H70	0.56257	0.69097	0.80971
H	H71	0.18283	0.18613	0.74918
H	H72	0.19057	0.83172	0.1503
N	N119	0.35926	0.0812	0.37758
N	N120	0.36244	0.76366	0.48852
N	N24	0.14494	0.68892	0.90003
N	N25	0.14411	0.37843	0.95585
N	N46	0.30557	0.12076	0.84469
N	N47	0.31104	0.77767	0.04329
N	N97	0.19682	0.6703	0.41084
N	N98	0.19309	0.33991	0.45583
S	S148	0.13782	0.28621	0.30044
S	S149	0.08418	0.32723	0.81322
S	S150	0.1407	0.78138	0.55254
S	S151	0.08994	0.79261	0.07456
S	S152	0.41199	0.13428	0.54744
S	S153	0.3631	0.17001	0.00947
S	S154	0.36467	0.67637	0.85853
S	S155	0.41628	0.65571	0.33118
S	S156	0.88188	0.44729	0.31072
S	S157	0.7936	0.50126	0.34688
S	S158	0.75013	0.56837	0.18695

S	S2	0.64272	0.84516	0.92885
S	S3	0.64449	0.72287	0.93051
S	S6	0.79676	0.77009	0.89836
S	S7	0.79809	0.64798	0.87589
S	S75	0.70409	0.81305	0.45625
S	S76	0.705	0.69136	0.43947
S	S79	0.85915	0.73484	0.44836
S	S80	0.8578	0.61549	0.40321

Table S13 Atomic coordinates in the unit cell of R-TTF$^{\bullet+}$-COF stabilized by TFSI^{-}.

Atom	Label	x	y	z
C	C0	0.57958	0.82607	0.93495
C	C1	0.57789	0.76989	0.94388
C	C10	0.94076	0.6816	0.94395
C	C100	0.25741	0.25343	0.46221
C	C101	0.31904	0.22587	0.5216
C	C102	0.23608	0.21449	0.38235
C	C103	0.22646	0.73914	0.49452
C	C104	0.30976	0.64572	0.35743
C	C105	0.58128	0.86475	0.43705

C	C106	0.58251	0.91442	0.52768
C	C107	0.53024	0.97536	0.53508
C	C108	0.47398	0.98995	0.45362
C	C109	0.47327	0.94053	0.3602
C	C11	0.94299	0.54915	0.92902
C	C110	0.52548	0.88034	0.35104
C	C111	0.5798	0.73193	0.42673
C	C112	0.52831	0.76902	0.52817
C	C113	0.47481	0.76296	0.52472
C	C114	0.47009	0.71779	0.42199
C	C115	0.52237	0.67896	0.3254
C	C116	0.57557	0.68584	0.3271
C	C117	0.41298	0.71108	0.41975
C	C118	0.41727	0.05416	0.45837
C	C12	0.94389	0.72684	0.84243
C	C121	0.31852	0.12994	0.41212
C	C122	0.33939	0.16846	0.49631
C	C123	0.25661	0.15729	0.35518
C	C124	0.31498	0.73262	0.46548
C	C125	0.25328	0.76621	0.52126
C	C126	0.33618	0.67319	0.38233
C	C13	0.99276	0.6451	0.04565
C	C14	0.04542	0.65262	0.04571
C	C15	0.04821	0.69825	0.9452
C	C159	0.71741	0.93462	0.17345
C	C16	0.99626	0.73542	0.84457
C	C160	0.78396	0.8688	0.73815
C	C17	0.99407	0.53852	0.83148
C	C18	0.94889	0.49672	0.01858
C	C19	0.00331	0.43745	0.01318
C	C20	0.05488	0.42794	0.92068
C	C21	0.04884	0.47987	0.82902
C	C22	0.11308	0.36414	0.91291

C	C23	0.10445	0.70744	0.952
C	C26	0.20151	0.68961	0.91578
C	C27	0.21156	0.28674	0.94878
C	C28	0.2731	0.25658	0.00879
C	C29	0.19003	0.25147	0.85209
C	C30	0.17936	0.74614	0.01281
C	C31	0.26259	0.65967	0.85635
C	C32	0.52663	0.89318	0.92218
C	C33	0.52959	0.94389	0.00222
C	C34	0.47953	0.00607	0.99812
C	C35	0.42368	0.02134	0.91394
C	C36	0.42159	0.97127	0.8273
C	C37	0.47158	0.90935	0.83095
C	C38	0.52303	0.75885	0.95003
C	C39	0.46707	0.80034	0.0314
C	C4	0.70019	0.73493	0.94911
C	C40	0.41626	0.78972	0.03291
C	C41	0.41805	0.73646	0.95344
C	C42	0.47425	0.69404	0.87651
C	C43	0.52507	0.70482	0.87478
C	C44	0.36295	0.72648	0.94999
C	C45	0.36855	0.08646	0.91648
C	C48	0.27157	0.1654	0.8689
C	C49	0.29279	0.20027	0.96875
C	C5	0.76522	0.7017	0.95578
C	C50	0.20988	0.19551	0.80971
C	C51	0.26675	0.74276	0.99593
C	C52	0.20543	0.77286	0.05468
C	C53	0.28844	0.68667	0.897
C	C73	0.63605	0.79863	0.42908
C	C74	0.63529	0.74198	0.42355
C	C77	0.75648	0.70899	0.41563
C	C78	0.82176	0.67631	0.41361

C	C8	0.88541	0.67101	0.94571
C	C81	0.94242	0.64595	0.43101
C	C82	0.94371	0.58806	0.41727
C	C83	0.99465	0.66103	0.45289
C	C84	0.998	0.52153	0.40874
C	C85	0.98761	0.72035	0.40536
C	C86	0.05308	0.61777	0.52213
C	C87	0.10287	0.63001	0.52699
C	C88	0.09652	0.68783	0.4696
C	C89	0.03692	0.73364	0.41579
C	C9	0.88636	0.61376	0.93991
C	C90	0.05484	0.50703	0.33392
C	C91	0.99463	0.46922	0.47306
C	C92	0.04528	0.40724	0.46827
C	C93	0.10299	0.39431	0.40469
C	C94	0.10611	0.44581	0.33479
C	C95	0.15937	0.32943	0.41068
C	C96	0.15161	0.69833	0.46609
C	C99	0.24854	0.67872	0.41598
F	F165	0.80386	0.81121	0.66355
F	F166	0.72413	0.89004	0.77696
F	F167	0.81818	0.85852	0.87744
F	F168	0.77205	0.87968	0.16933
F	F169	0.7078	0.96436	0.02779
F	F170	0.6722	0.91795	0.19135
H	H127	0.94343	0.75761	0.35666
H	H128	0.06065	0.57289	0.57162
H	H129	0.14726	0.5947	0.57887
H	H130	0.02857	0.78004	0.37519
H	H131	0.06019	0.54433	0.27565
H	H132	0.95236	0.47668	0.53294
H	H133	0.04018	0.3689	0.52156
H	H134	0.14906	0.43735	0.27722

H	H135	0.33496	0.25539	0.58467
H	H136	0.32606	0.60022	0.2952
H	H137	0.62459	0.90649	0.59323
H	H138	0.53454	0.01159	0.60644
H	H139	0.43124	0.9505	0.29155
H	H140	0.52335	0.84426	0.27437
H	H141	0.53045	0.80359	0.61118
H	H142	0.43604	0.79295	0.6043
H	H143	0.52158	0.64338	0.24301
H	H144	0.6138	0.65547	0.24654
H	H145	0.24083	0.12781	0.29121
H	H146	0.237	0.81157	0.58423
H	H147	0.27913	0.6172	0.78004
H	H54	0.90557	0.75609	0.75978
H	H55	0.99159	0.61005	0.12576
H	H56	0.0844	0.62364	0.12748
H	H57	0.99601	0.77151	0.76471
H	H58	0.99096	0.57704	0.75647
H	H59	0.91094	0.50207	0.09596
H	H60	0.00584	0.39846	0.08526
H	H61	0.08721	0.47394	0.7524
H	H62	0.28933	0.283	0.08515
H	H63	0.57071	0.93527	0.0737
H	H64	0.4847	0.04253	0.06583
H	H65	0.37985	0.98152	0.75678
H	H66	0.467	0.87281	0.76261
H	H67	0.46253	0.84248	0.09391
H	H68	0.37409	0.82315	0.09667
H	H69	0.47878	0.65199	0.81285
H	H70	0.56646	0.67105	0.80878
H	H71	0.19355	0.16887	0.73474
H	H72	0.18861	0.81578	0.12924
N	N119	0.36359	0.0665	0.39299

N	N120	0.35966	0.75183	0.48458
N	N157	0.73741	0.94112	0.49145
N	N24	0.15807	0.66916	0.88602
N	N25	0.16691	0.35022	0.97812
N	N46	0.31542	0.10197	0.84245
N	N47	0.31002	0.76347	0.02248
N	N97	0.20539	0.65726	0.40474
N	N98	0.213	0.31787	0.47325
O	O161	0.85629	0.87767	0.52754
O	O162	0.7565	0.01142	0.29304
O	O163	0.64805	0.03224	0.34928
O	O164	0.79486	0.97209	0.70781
S	S148	0.15779	0.26218	0.32826
S	S149	0.11147	0.30082	0.80589
S	S150	0.1487	0.768	0.5472
S	S151	0.10161	0.77273	0.05997
S	S152	0.41751	0.12082	0.55107
S	S153	0.36985	0.14955	0.02651
S	S154	0.36488	0.66166	0.84058
S	S155	0.41361	0.64379	0.32797
S	S156	0.79729	0.92199	0.60269
S	S158	0.7143	0.98894	0.34098
S	S2	0.65493	0.81833	0.93715
S	S3	0.65214	0.69987	0.95172
S	S6	0.81129	0.73879	0.97087
S	S7	0.8122	0.61808	0.94992
S	S75	0.71062	0.7924	0.42107
S	S76	0.70959	0.67258	0.412
S	S79	0.86748	0.71364	0.41816
S	S80	0.8692	0.59324	0.40182

References

1. Mastronarde, D. N. Automated electron microscope tomography using robust prediction of specimen movements. *J. Struct. Biol.* **2005,** 152, 36–51.
2. Kohn, W. & Sham, L. J. Self-consistent equations including exchange and correlation effects. *Phys. Rev.* ***1965***, 140, 4A.
3. Kuhne, T. D. *et al.* CP2K: An electronic structure and molecular dynamics software package - Quickstep: Efficient and accurate electronic structure calculations. *J. Chem. Phys.* **2020**, 152, 194103.
4. Vandervelde, J. & Hutter, J. Gaussian basis sets for accurate calculations on molecular systems in gas and condensed phases. *J. Chem. Phys.* **2007**, 127, 114105.
5. Goedecker, S. Separable dual-space Gaussian pseudopotentials. *Phys. Rev.* **1995,** 54, 3.
6. Krack, M. Pseudopotentials for H to Kr optimized for gradient-corrected exchange-correlation functionals. *Theor. Chem. Acc.* **2005**, 114, 145–152.
7. Grimme, S., Antony, J., Ehrlich, S. & Krieg, H. A consistent and accurate *ab initio* parametrization of density functional dispersion correction (DFT-D) for the 94 elements H-Pu. *Chem. Phys.* **2010,** 132, 154104.
8. Perdew, J. P., Burke, K. & Ernzerhof, M. Generalized gradient approximation made simple. *Phys. Rev. Lett.* **1996**, 77, 18.
9. Adamo, C. & Barone, V. Toward reliable density functional methods without adjustable parameters: The PBE0 model. *J. Chem. Phys.* **1999**, 110, 6158–6170.
10. Heyd, J. & Scuseria, G. E. Assessment and validation of a screened Coulomb hybrid density functional. *J. Chem. Phys.* **2004**, 120, 7274–7280.
11. Guidon, M., Hutter, J. & Vande-Vondele, J. Auxiliary density matrix methods for Hartree-Fock exchange calculations. *J. Chem. Theory Comput.* **2010,** 6, 2348–2364.
12. Vicent-Morales, M. *et al.* Semiconductor porous hydrogen-bonded organic frameworks based on tetrathiafulvalene derivatives. *J. Am. Chem. Soc.* **2022,** 144, 9074–9082.
13. Rosokha, S. V. & Kochi, J. K. Molecular and electronic structures of the long-bonded π-dimers of tetrathiafulvalene cation-radical in intermolecular electron transfer and in solid-state Conductivity. *J. Am. Chem. Soc.* **2007**, 129, 828–838.

14. Oliveira, R. *et al.* Electrochemical activation of a tetrathiafulvalene halogen bond donor in solution. *Phys. Chem.* **2016**, 18, 15867–15873.

15. Wang, X. *et al.* Tetrathiafulvalene (TTF) derivatives as catholytes for dual-type redox flow batteries: molecular engineering enables high energy density and cyclability. *J. Mater. Chem. A* **2023**, 11, 19056–19065.

16. Hu, W. *et al.* Tetrathiafulvalene esters with high redox potentials and improved solubilities for non-aqueous redox flow battery applications. *Green Energy Environ.* **2024**, 9, 899–908.

17. Adeel, S. M., Martin, L. L. & Bond, A. M. Redox-induced solid-solid state transformation of tetrathiafulvalene (TTF) microcrystals into mixed-valence and π-dimers in the presence of nitrate anions. *J. Solid State Electr.* **2014**, 18, 3287–3298.

18. Xing, Y., Speiser, E., Singh, D. K., Dittrich, P. S. & Esser, N. Bi-axial growth mode of Au–TTF nanowires induced by tilted molecular column stacking. *J. Phys. Chem. C.* **2017**, 121, 23200–23206.

19. Nie, S. & Yu, N. T. Surface-enhanced near-infrared Fourier transform Raman scattering of tetrathiafulvalene adsorbed on silver powder. *J. Raman Spectrosc.* **2005**, 22, 489–495.

20. McBrayer J.D., Beechem T.E., Perdue B.R., Apblett C.A. & Garzon F.H. Polysulfide speciation in the bulk electrolyte of a lithium sulfur battery. *J. Electrochem. Soc.* **2018**, 165, A876–A881.

21. Hua W., Shang T., Li H., Sun Y., Guo Y., Xia J., Geng C., Hu Z., Peng L., Han Z., Zhang C., Lv W. & Wan Y. Optimizing the *p* charge of S in *p*-block metal sulfides for sulfur reduction electrocatalysis. *Nature Catalysis*, **2023**, 6, 174–184.

22. Hannauer J., Scheers J., Fullenwarth J., Fraisse B., Stievano L. & Johansson P. The quest for polysulfides in lithium–sulfur battery electrolytes: an operando confocal Raman spectroscopy study. *ChemPhysChem* **2015**, 16, 2755–2759.

23. Sadd M., Agostini M., Xiong S. & Matic A. Polysulfide speciation and migration in catholyte lithium–sulfur cells. *ChemPhysChem* **2022**, 23, e202100853.

24. Partovi-Azar P., Kühne T.D. & Kaghazchi P. Evidence for the existence of Li_2S_2 clusters in lithium–sulfur batteries: ab initio Raman spectroscopy simulation. *Phys. Chem. Chem. Phys.* **2015**, 17, 22009.

25. Lang S., Yu S.-H., Feng X., Krumov M.R. & Abruña H.D. Understanding the lithium–sulfur battery redox reactions via operando confocal Raman microscopy. *Nat. Commun.* **2022**, 13, 4811.

26. Liu R., Wei Z., Peng L., Zhang L., Zohar A., Schoeppner R., Wang P., Wan C., Zhu D., Liu H., Wang Z.,

Tolbert S.H., Dunn B., Huang Y., Sautet P. & Duan X. Establishing reaction networks in the 16-electron sulfur reduction reaction. *Nature* **2024**, 626, 1.

27. Liu M., Wu Z., Liu S., Guo T., Chen P., Cao X., Pan S., Zhou T., Pompizii L., Najafov M., Coskun A. & Fu Y. Accelerated reversible conversion of Li2S2 to Li2S by spidroin regulated Li+ flux for high-performance Li-sulfur batteries. *Angew. Chem. Int. Ed.* **2025**, 64, e202417624.

28. Wang S., Lu B., Cheng D., Wu Z., Feng S., Zhang M., Li W., Miao Q., Patel M., Feng J., Hopkins E., Zhou J., Parab S., Bhamwala B., Liaw B., Meng Y. & Liu P. Structural transformation in a sulfurized polymer cathode to enable long-life rechargeable lithium-sulfur batteries. *J. Am. Chem. Soc.* **2023**, 145, 9624–9633.

29. Li G., Liu Y., Schultz T., Exner M., Muydinov R., Wang H., Scheurell K., Huang J., Szymoniak P., Pinna N., Koch N., Adelhelm P. & Bojdys M.J. One-pot synthesis of high-capacity sulfur cathodes via in-situ polymerization of a porous imine-based polymer. *Angew. Chem. Int. Ed.* **2024**, 63, e202400382.

30. Pan H., Han K., Engelhard M.H., Cao R., Chen J., Zhang J.-G., Mueller K.T., Shao Y. & Liu J. Addressing passivation in lithium-sulfur battery under lean electrolyte condition. *Adv. Funct. Mater.* **2018**, 28, 1707234.

31. Liang X., Hart C., Pang Q., Garsuch A., Weiss T. & Nazar L.F. A highly efficient polysulfide mediator for lithium–sulfur batteries. *Nat. Commun.* **2015**, 6, 5682.

32. Wang Z.-Y., Wang L., Liu S., Li G.-R. & Gao X.-P. Conductive CoOOH as carbon-free sulfur immobilizer to fabricate sulfur-based composite for lithium–sulfur battery. *Adv. Funct. Mater.* **2019**, 29, 1901051.

33. Kim J.T., Rao A., Nie H.-Y., Hu Y., Li W., Zhao F., Deng S., Hao X., Fu J., Luo J., Duan H., Wang C., Singh C.V. & Sun X. Manipulating Li2S2/Li2S mixed discharge products of all-solid-state lithium sulfur batteries for improved cycle life. *Nat. Commun.* **2023**, 14, 6404.

34. Li, Z., Zhou, Y., Wang, Y. & Lu, Y. C. Solvent-mediated Li_2S electrodeposition: a critical manipulator in lithium–sulfur batteries. *Adv. Energy Mater.* **2018**, 9, 1802207.

35. Huang, X. *et al.* Cyclic voltammetry in lithium–sulfur batteries—challenges and opportunities. *Energy Technol.* **2019**, 7, 1801001.

36. Liu, X. F., Chen, H., Wang, R., Zang, S. Q. & Mak, T. C. W. Cationic covalent-organic framework as efficient redox motor for high-performance lithium-sulfur batteries. *Small* **2020**, 16, e2002932.

37. Liu, W. *et al.* Conjugated three-dimensional high-connected covalent organic frameworks for lithium-sulfur batteries. *J. Am. Chem. Soc.* **2022**, 144, 17209–17218.

38. Lu, B. Y. *et al.* A covalent organic framework with extended pi-conjugated building units as a highly efficient recipient for lithium-sulfur batteries. *ACS Appl. Mater. Interfaces* **2020**, 12, 34990–34998.

39. Liang, Y. *et al.* Boric acid functionalized triazine-based covalent organic frameworks with dual-function for selective adsorption and lithium-sulfur battery cathode. *Chem. Eng. J.* **2022,** 437, 135314.

40. Duan, H. *et al.* Scalable synthesis of ultrathin polyimide covalent organic framework nanosheets for high-performance lithium-sulfur batteries. *J. Am. Chem. Soc.* **2021, 143**, 19446–19453.

41. Yan, R. *et al.* A thiazole-linked covalent organic framework for lithium-sulphur batteries. *Angew. Chem. Int. Ed. Engl.* **2023**, 62, e202302276.

42. Li, X. *et al.* Constructing ambivalent imidazopyridinium-linked covalent organic frameworks. *Nat. Synth.* **2022**, 1, 382–392.

43. Lv, S. *et al.* Metal-coordinated covalent organic frameworks as advanced bifunctional hosts for both sulfur cathodes and lithium anodes in lithium-sulfur batteries. *J. Am. Chem. Soc.* **2024**, 146, 9385–9394.

44. Ge, Y., Li, J., Meng, Y. & Xiao, D. Tuning the structure characteristic of the flexible covalent organic framework (COF) meets a high performance for lithium-sulfur batteries. *Nano Energy* **2023**, 109.

45. Klein, M. J., Goossens, K., Bielawski, C. W. & Manthiram, A. Elucidating the electrochemical activity of electrolyte-insoluble polysulfide species in lithium-sulfur batteries. *J. Electro. Soc.* **2016**, 163, A2109–A2116.

46. Wang, S. *et al.* Structural transformation in a sulfurized polymer cathode to enable long-life rechargeable lithium-sulfur batteries. *J. Am. Chem. Soc.* **2023**, 145, 9624–9633.

47. Hardwick, L. J., Saint, J. A., Lucas, I. T., Doeff, M. M., & Kosteckia, R. FTIR and Raman study of the $Li_xTi_yMn_{1-y}O_2$ y = 0, 0.11... cathodes in methylpropyl pyrrolidinium bis(fluoro-sulfonyl)imide, LiTFSI electrolyte. *J. Electro. Soc.* **2009**, 156, A120–A127.

48. Bai, H., Wang, X., Zhou, Y. & Zhang, L. Preparation and characterization of poly(vinylidene fluoride) composite membranes blended with nano-crystalline cellulose. *Prog. Nat. Sci.: Mater. Int.* **2012,** 22, 250–257.

49. Zhang, M. *et al.* Unveiling the structural and dynamic characteristics of concentrated $LiNO_3$ aqueous solutions through ultrafast infrared spectroscopy and molecular dynamics simulations. *J. Phys. Chem. Lett.* **2024,** 15, 7610–7619.

50. Gómez-serrano, V., Fernández-gonzález, M. C., Rojas-cervantes, M. L., Alexandre-franco, M. F. & Macías-garcía, A. Carbonization and demineralization of coals: A study by means of FT–IR spectroscopy. *Bull. Mater. Sci.,* **2003**, 26, 721–732.

51. Wiles, D. M., Gingras, B. A. & Suprunchuk, T. The C=S stretching vibration in the infrared spectra of some thiosemicarbazones. *Can. J. Chem.* **1967**, 45, 469–473.
